# Ruthenium-Catalyzed Intramolecular [2+2+2] Cycloaddition and Tandem Cross-Metathesis of Triynes and Enediynes

**DOI:** 10.1002/open.201300002

**Published:** 2013-02-19

**Authors:** Wei Yuan, Yin Wei, Min Shi

**Affiliations:** [a] State Key Laboratory of Organometallic Chemistry, Shanghai Institute of Organic Chemistry, Chinese Academy of Sciences354 Fenglin Lu, Shanghai 200032 (P. R. China) E-mail: Mshi@mail.sioc.ac.cn

**Keywords:** cross-metathesis, cycloaddition, enediynes, first-generation Grubbs catalyst, triynes

## Abstract

[2+2+2] Cycloadditions can be applied to specifically build up derivatives of benzene and cyclohexadiene and, therefore, have attracted much attention. Herein, we present an intramolecular [2+2+2] cycloaddition of triynes catalyzed by the first-generation Grubbs ruthenium complex (**Ru gen-1**), which can efficiently afford benzene derivatives in good yields under mild conditions. Moreover, we also report on a novel tandem cross-metathesis transformation of intramolecular enediynes also catalyzed by **Ru gen-1**, which has not been observed previously in related reports. On the basis of deuterium labeling experiments, a possible reaction mechanism is presented.

## Introduction

Cycloisomerization and cycloaddition reactions of enyne substrates have witnessed significant developments in the past decade due to their convenience and versatility in constructing complicated ring structures and useful intermediates in the synthesis of natural products.^1^ Among these myriad transformations, intramolecular/intermolecular [2+2+2] cycloadditions of triynes and enediynes catalyzed by transition metals have attracted even more attention, since these types of [2+2+2] cycloadditions can be applied to specifically build up the derivatives of benzene and cyclohexadiene.^2^, ^3^ However, there are not many reports on such [2+2+2] additions catalyzed by the Grubbs ruthenium complex when searching through previous literature.^4^–^8^ Herein, we present an intramolecular [2+2+2] cycloaddition of triynes catalyzed by the first-generation Grubbs ruthenium complex (**Ru gen-1**), which can efficiently afford benzene derivatives in good yields under mild conditions. Moreover, we also disclose a novel tandem cross-metathesis transformation of intramolecular enediynes catalyzed by **Ru gen-1** in this paper, which has not been observed previously in related reports.

**Figure 1:**
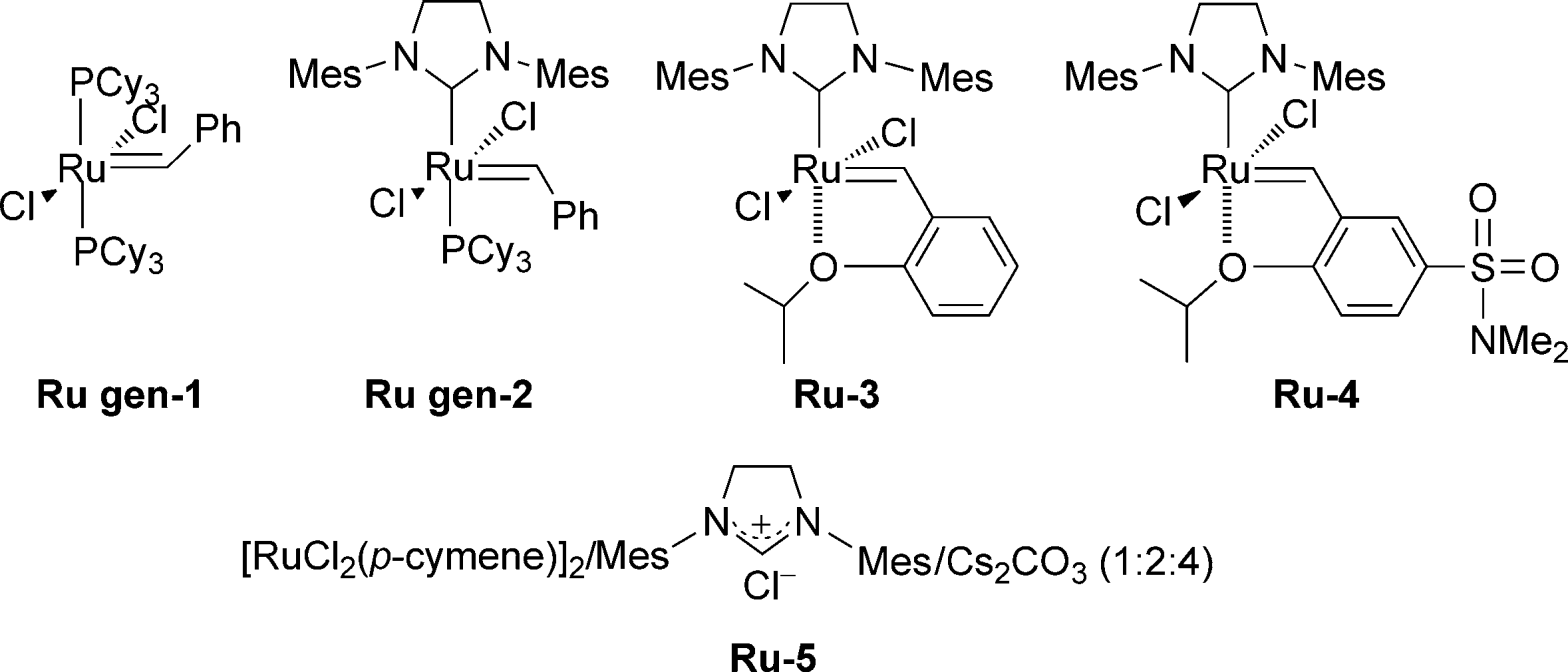
Ruthenium catalysts used in intramolecular cycloaddition and tandem cross-metathesis reactions of triynes and enediynes (Mes: 2,4,6-trimethylphenyl).

Figure 1 shows the ruthenium catalysts that are used in this work of intramolecular cycloaddition and tandem cross-metathesis reactions of triynes and enediynes. **Ru gen-1** and **Ru gen-2** are the first and second generation of Grubbs ruthenium complexes that have been widely used in olefin metathesis. **Ru-3** is the Hoveyda–Grubbs catalyst that was developed in the Hoveyda group.^9^ **Ru-4** is a modified Hoveyda–Grubbs catalyst developed by Zhan.9c Catalyst kits **Ru-5** developed by Dixneuf’s group has also been widely used in enyne metathesis.^10^

Initial examination of the intramolecular [2+2+2] cycloaddition of triynes was performed by using triyne **1 a** (0.1 mmol) as the substrate in the presence of **Ru gen-1** (10 mol %), and we found that the benzene derivative **2 a** was formed in 84 % yield within 12 h in styrene at room temperature (determined using ^1^H NMR with 1,3,5-trimethoxybenzene as an internal standard; Table 1, Entry 1). Using 5 mol % of **Ru gen-1** as the catalyst, afforded **2 a** in 74 % yield (Table 1, Entry 2). The reaction conditions were optimized, and the results are summarized in Table 1. As shown, the examination of solvent effects revealed that dichloromethane is the suitable solvent, giving **2 a** in 88 % ^1^H NMR yield (80 % isolated yield; Table 1, Entries 3–7). Moreover, the yield of **2 a** decreased together with the catalyst loading of **Ru gen-1** from 10 to 5 mol % (Table 1, Entry 8). On the basis of screening other ruthenium and rhodium catalysts, we found that **Ru gen-1** is the most efficient catalyst for this [2+2+2] cycloaddition, although **2 a** could be given in 85 % yield, when Ru(PPh_3_)_2_CpCl (10 mol %) was employed as the catalyst (Table 1, Entries 9–12). Pd(PPh_3_)_2_Cl_2_ or PtCl_2_ did not catalyze this reaction under otherwise identical conditions (Table 1, Entries 12–14). Thus, we identified that using dichloromethane as the solvent and 10 mol % of **Ru gen-1** as the catalyst, **2 a** could be obtained in the best yield (Table 1, Entry 7).

**Table 1:** Optimization of the reaction conditions for the [2+2+2] cycloaddition reactions of intramolecular triynes catalyzed by Ru gen-1.[a]

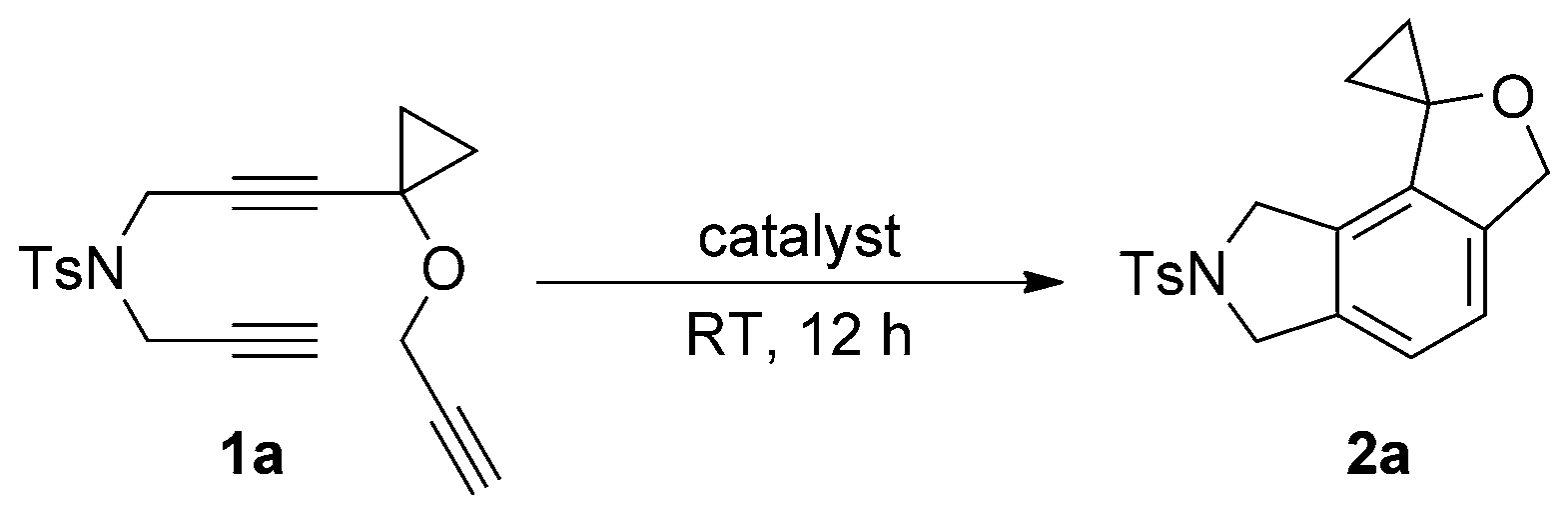				

Entry	Catalyst	Catalyst [mol %]	Solvent	Yield2 a [%][b]
1	**Ru gen-1**	10	styrene	84
2	**Ru gen-1**	5	styrene	74
3	**Ru gen-1**	10	toluene	37[c]
4	**Ru gen-1**	10	DCE	39
5	**Ru gen-1**	10	THF	38
6	**Ru gen-1**	10	CH_3_CN	–
7	**Ru gen-1**	10	CH_2_Cl_2_	88 (80)[c]
8	**Ru gen-1**	5	CH_2_Cl_2_	68
9	Ru(PPh_3_)_2_CpCl	10	CH_2_Cl_2_	85
10	Rh(PPh_3_)_3_Cl	10	CH_2_Cl_2_	71
11	[Rh(CO)_2_Cl]_2_	10	CH_2_Cl_2_	complex
12	[Rh(COD)Cl]_2_	10	CH_2_Cl_2_	76[c]
13	Pd(PPh_3_)_2_Cl_2_	10	CH_2_Cl_2_	–
14	PtCl_2_	5	CH_2_Cl_2_	–

[a] *Reagents and conditions*: triyne substrate **1 a** (0.2 mmol), catalyst, solvent (2 mL), RT, 12 h under argon.

[b] Yields were determined using ^1^H NMR and 1,3,5-trimethoxybenzene as an internal standard.

[c] Isolated yields. DCE: 1,2-dichloroethane, THF: tetrahydrofuran.

Under the optimized reaction conditions, the substrate scope and limitations of the reaction were explored and the results are summarized in Table 2. As for triyne substrates **1 a**–**c** bearing cyclopropane rings, the reactions proceeded smoothly to give the corresponding products **2 a**–**c** in 80–86 % yields (Table 2, Entries 1–3). When triyne substrates **1 d**–**g**, which do not have a cyclopropyl group, were employed as substrates, the corresponding [2+2+2] cycloaddition products **2 d**–**g** could be obtained in 66 %-94 % yields (Table 2, Entries 4–7). Furthermore, using triyne substrates **1 h**–**k** in which R^1^, R^2^ and R^3^ are different substituents (R^1^ or R^2^=*n*Pr or Ph, R^3^=H; R^1^=R^2^=H, R^3^=Me or Ph) as the substrates, the desired products **2 h**–**k** were obtained in moderate to good yields ranging from 55 % to 92 % (Table 2, Entries 8–11). Finally, in the case of triyne substrate **1 l**, in which one carbon chain has been extended to a CH_2_CH_2_ moiety, the corresponding product **2 l** was also formed in 92 % yield (Table 2, Entry 12). Their structures have been assigned by spectroscopic data. Moreover, product **2 g** is a known compound and its spectroscopic data are consistent with those in the literature.^11^

**Table 2:** Substrate scope of the intramolecular [2+2+2] cycloaddition reactions of triynes catalyzed by Ru gen-1.[a]

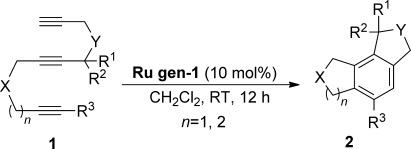												

Entry		Compound 1		Product 2	Yield 2[%][b]		Entry		Compound 1		Product 2	Yield 2[%][b]
1	**1 a**	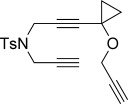	**2 a**	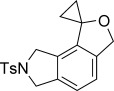	80		7	**1 g**	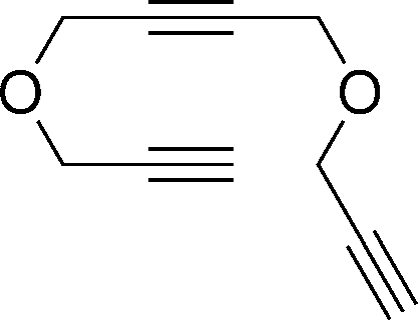	**2 g**	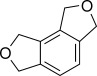	95
2	**1 b**	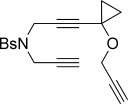	**2 b**	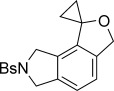	85		8	**1 h**	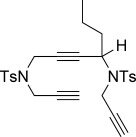	**2 h**	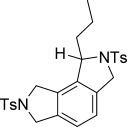	83
3	**1 c**	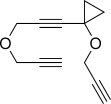	**2 c**	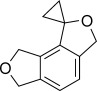	86		9	**1 i**	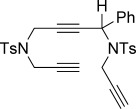	**2 i**	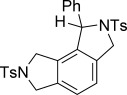	55
4	**1 d**	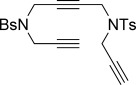	**2 d**	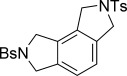	77		10	**1 j**	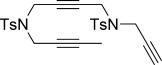	**2 j**	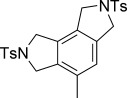	90
5	**1 e**	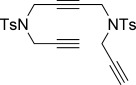	**2 e**	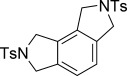	94		11	**1 k**	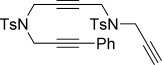	**2 k**	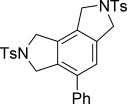	58
6	**1 f**	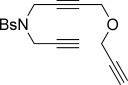	**2 f**	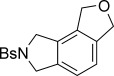	66		12	**1 l**	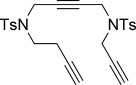	**2 l**	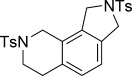	92

[a] *Reagents and conditions:* triyne substrate **1** (0.2 mmol), **Ru gen-1** (10 mol %), CH_2_Cl_2_ (2 mL), RT, 12 h under argon.

[b] Isolated yields. Bs: bromobenzenesulfonyl, Ts: 4-toluenesulfonyl.

Next, we attempted to explore the reaction outcome of enediynes, in which one terminal propargyl group in substrate **1** has been changed to a vinyl group, under standard conditions. Initial examination was performed by using enediyne **3 a** (0.1 mmol) as the substrate in the presence of **Ru gen-1** (10 mol %) in styrene at room temperature. As shown in Table 3, we found that the intramolecular tandem cross-metathesis took place, affording **4 a** in 27 % isolated yield (Table 3, Entry 1). The examination of solvent effects revealed that 1,2-dichloroethane (DCE) is a suitable solvent for this tandem cross-metathesis (Table 3, Entries 2–8). The other ruthenium catalysts, such as **Ru gen-2**, **Ru-3**, **Ru-4** and **Ru-5**, did not produce the desired product under similar conditions (Table 3, Entries 9–12). Moreover, the additive effects such as styrene, Ti(O*i*Pr)_4_, and hydroquinone have also been examined under the tentatively optimized conditions, but no significant improvement could be observed (for detailed results, see Table SI-1 in the Supporting Information). Eventually, we identified that using DCE as the solvent with 10 mol % of catalyst loading (**Ru gen-1**), **4 a** could be obtained in 52 % isolated yield at 70 °C within 12 h, which served as the best reaction conditions for this reaction (Table 3, Entry 13).

**Table 3:** Optimization of the reaction conditions for the intramolecular tandem cross-metathesis reactions of enediynes.[a]

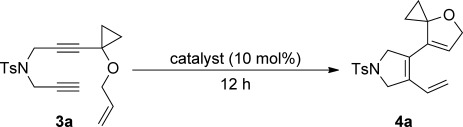				

Entry	Catalyst	Solvent	*T* [° C]	Yield4 a [%][b]
1	**Ru gen-1**	styrene	RT	27[c]
2	**Ru gen-1**	CH_2_Cl_2_	RT	15
3	**Ru gen-1**	THF	RT	10
4	**Ru gen-1**	DMF	RT	–
5	**Ru gen-1**	CH_3_CN	RT	–
6	**Ru gen-1**	DCE	RT	37
7	**Ru gen-1**	toluene	RT	16
8	**Ru gen-1**	1,4-dioxane	RT	complex
9	**Ru gen-2**	CH_2_Cl_2_	RT	–
10	**Ru-3**	DCE	70	complex
11	**Ru-4**	CH_2_Cl_2_	RT	–
12	**Ru-5**	CH_2_Cl_2_	RT	–
13	**Ru gen-1**	DCE	70	63 (52)[c]

[a] *Reagents and conditions*: enyne substrate **3 a** (0.1 mmol), catalyst (10 mol %), solvent (1.0 mL), 12 h under argon.

[b] The yield was determined using ^1^H NMR and 1,3,5-trimethoxybenzene as an internal standard.

[c] Isolated yields. Ts: 4-toluenesulfonyl, THF: tetrahydrofuran, DMF: *N*,*N*-dimethylformamide, DCE: 1,2-dichloroethane.

Under the optimized reaction conditions, the substrate scope and limitations of the reaction were also explored, and the results are summarized in Table 4. As for substrates **3 a** and **3 b** bearing cyclopropane rings, the reaction proceeded smoothly to give the corresponding products **4 a** and **4 b** in 52 % and 54 % yields, respectively (Table 4, Entries 1 and 2). When enediyne substrates **3 c**–**h** (R^1^=R^2^=R^3^=H; X=TsN, BsN, O or C; Y=O or TsN) were employed as substrates, the corresponding products **4 c**–**h** could be obtained in 55 %–68 % yields, respectively (Table 4, Entries 3–8). However, using enediyne substrate **3 i** or **3 j**, in which the terminal C atom of the propargyl group carries a methyl or phenyl group, the reaction gave complex product mixtures under the standard conditions (Table 4, Entries 9 and 10). In the case of triyne substrates **3 k** and **3 l**, in which one carbon chain has been extended as a CH_2_CH_2_ moiety, the corresponding hexatriene derivatives **4 k** and **4 l** were afforded in 67 % or 75 % yields, respectively, rather than the cross-metathesis reaction products (Table 4, Entries 11 and 12). On the basis of previous literature, it could be rationalized that the products **4 k** and **4 l** were derived from the energetically favored 6π-electrocyclization of the corresponding tandem cross-metathesis products.^12^ Finally, using enediyne substrate **3 m**, in which the terminal C atom of the allyl group is attached to a phenyl group, no reaction occurred under the standard conditions (Table 4, Entry 13).

**Table 4:** Substrate scope of tandem intramolecular cross-metathesis reactions of enediynes catalyzed by Ru gen-1.[a]

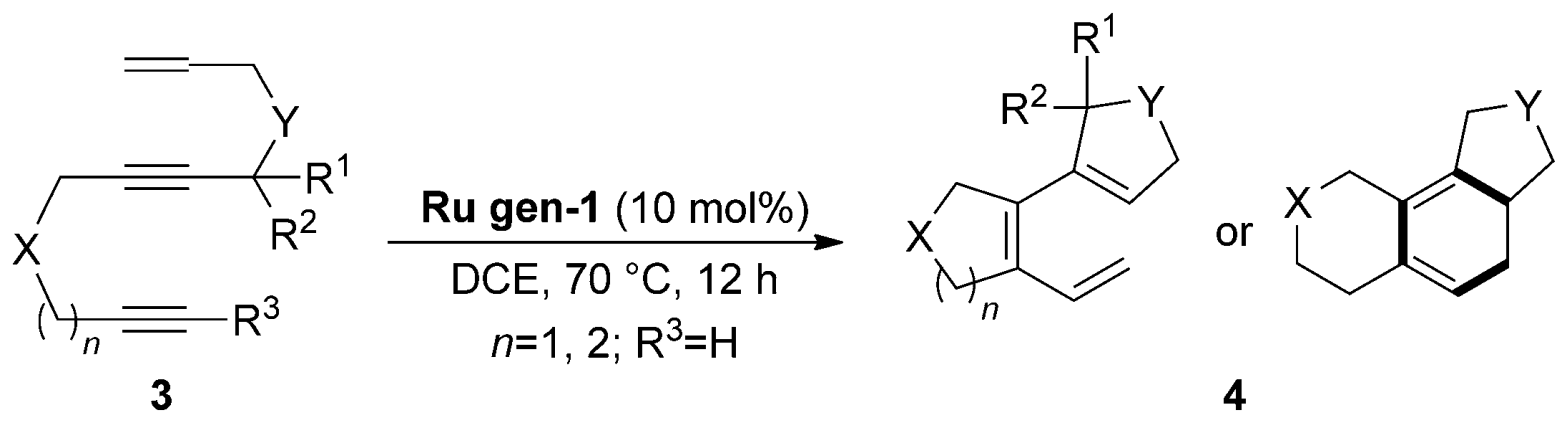											

Entry		Compound 3		Product 4	Yield 4[%][b]	Entry		Compound 3		Product 4	Yield 4[%][b]
1	**3 a**	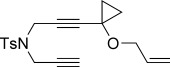	**4 a**	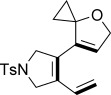	55	7	**3 g**	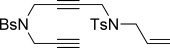	**4 g**	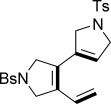	61
2	**3 b**	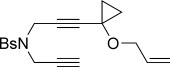	**4 b**	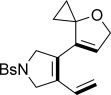	54	8	**3 h**	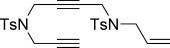	**4 h**	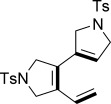	55
3	**3 c**	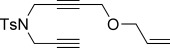	**4 c**	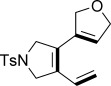	68	9	**3 i**	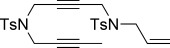	–	–	complex
4	**3 d**	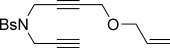	**4 d**	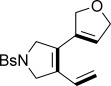	68	10	**3 j**	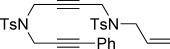	–	–	complex
5	**3 e**	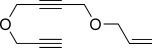	**4 e**	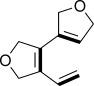	64	11[c]	**3 k**	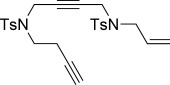	**4 k**	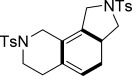	67
6	**3 f**	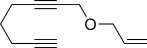	**4 f**	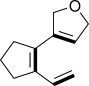	68	12[c]	**3 l**	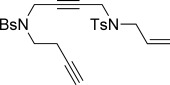	**4 l**	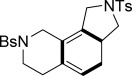	75
						13	**3 m**	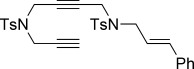	**3 m**	–	N.R.^[d]^

[a] *Reagents and conditions*: substrate **3** (0.2 mmol), **Ru gen-1** (10 mol %), DCE (2.0 mL), 70 °C, 12 h under argon.

[b] Isolated yields.

[c] Derived from a 6π-electrocyclization of the corresponding tandem cross-metathesis products.

[d] No reaction. Bs: bromobenzenesulfonyl, Ts: 4-toluenesulfonyl.

**Scheme 1:**
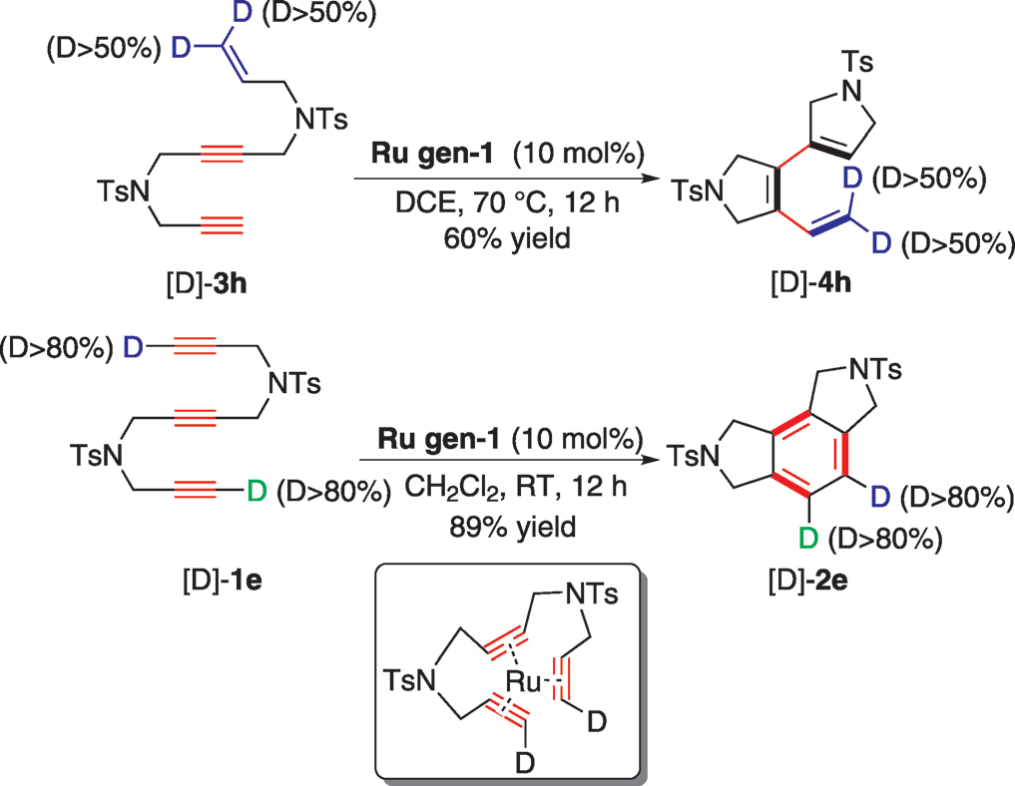
Isotope labeling experiments (Ts: 4-toluenesulfonyl).

It seems to us that the corresponding products **4 a**–**h** were produced via a tandem cross-metathesis process, since Grubbs ruthenium complex (**Ru gen-1**) is also an effective catalyst in enyne metathesis.^[4^ ^h, 4i]^ In order to gain more mechanistic insights into the reaction, we conducted an isotope labeling experiment to examine the reaction outcome by using dideuterated [D]-**3 h** (deuterium content=54 %) as the reactant, and the reaction was carried out under the standard conditions (Scheme 1; for details, see the Supporting Information). It was found that product [D]-**4 h** could be obtained in 60 % yield along with 54 % deuterium content, clearly suggesting a cross-metathesis process.

On the other hand, using dideuterated substrate [D]-**1 e** (deuterium content>80 %) in the reaction afforded the corresponding product [D]-**2 e** in 89 % yield along with 80 % deuterium content under the standard conditions (Scheme 1; for details, see the Supporting Information), suggesting a specific intramolecular [2+2+2] cycloaddition process.

**Scheme 2:**
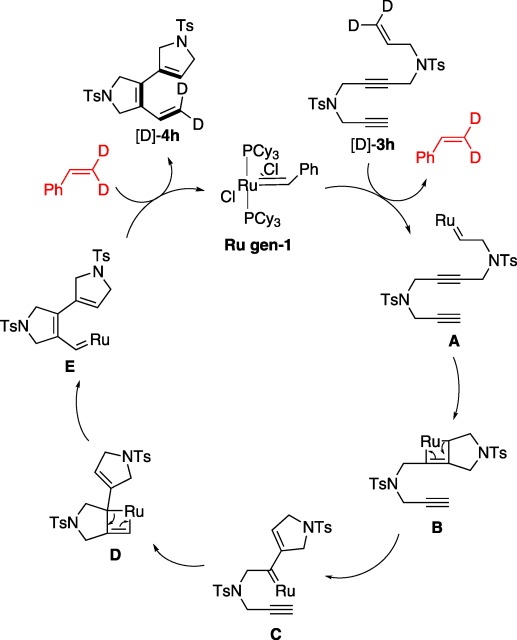
A possible reaction mechanism for the formation of [D]-**4 h** (Ts: 4-toluenesulfonyl).

On the basis of the above results, the deuterium labeling experiments and the previous literature,^8^, ^13^ the mechanism for the formation of **4** is outlined in Scheme 2 by using [D]-**3 h** as a reaction model. Initial reaction of **Ru gen-1** with the olefin moiety of [D]-**3 h** generates methylene ruthenium intermediate **A** along with the release of dideuterated styrene. The intramolecular [2+2] cycloaddition of carbene intermediate **A** with the adjacent alkyne moiety produces ruthenacyclobutene **B**, which undergoes a ring-opening process to give internal vinyl carbene intermediate **C**. Then, vinyl carbene intermediate **C** undergoes intramolecular [2+2] cycloaddition with the second alkyne moiety to give another ruthenacyclobutene **D**, which again undergoes a ring opening process to give carbene intermediate **E**. The reaction of intermediate **E** with the released dideuterated styrene gives the desired product [D]-**4 h** as well as the catalyst engaging in the next catalytic cycle (Scheme 2). It should be noted that this intramolecular tandem cross-metathesis of enediynes could also be initiated from the terminal alkyne side (see Scheme SI-1 in the Supporting Information). However, because none of the desired products were formed in the cases of **3 i**, **3 l** and **3 m**, at the present stage, we assumed that the mechanism shown in Scheme 2 might be more reasonable.

In conclusion, we reported on intramolecular [2+2+2] cycloaddition and tandem cross-metathesis reactions of triynes and enediynes, respectively, catalyzed by **Ru gen-1** that can specifically produce the corresponding benzene derivatives **2** as well as the conjugated triene derivatives **4** in moderate to good yields. The real catalytic species is Ru-gen 1 rather than others. A plausible reaction mechanism for the formation of **4** has also been proposed on the basis of deuterium labeling experiments and the previous literature. Further investigations on the mechanistic details as well as the substrate scope of the reaction are in progress.

## Experimental Section

Detailed descriptions of the experimental procedures as well as the spectroscopic data of the compounds shown in Tables 1–4 and the 2D spectra of **4 h** and **4 l** (COSY, NOESY, HMQC, HMBC and DEPT) are shown in the Supporting Information.

**Ruthenium-catalyzed [2+2+2] intramolecular cycloaddition of triynes:** Substrate **1** (0.2 mmol), first-generation Grubbs catalyst (10 mol %) and CH_2_Cl_2_ (2.0 mL) was added to a flame-dried Schlenk tube, and the resulting solution was stirred at RT for 12 h. The reaction mixture was concentrated in vacuo, and the residue was purified by flash silica gel column chromatography (pentane/EtOAc, 10:1–4:1).

**Compound 2 a:** White solid (57 mg, 80 %,): mp: 217–219 °C; ^1^H NMR (CDCl_3_, 300 MHz, TMS): *δ*=1.02 (dd, *J*_1_=8.4 Hz, *J*_2_=6.0 Hz, 2 H, CH_2_), 1.23 (dd, *J*_1_=8.4 Hz, *J*_2_=6.0 Hz, 2 H, CH_2_), 2.41 (s, 3 H, CH_3_), 4.40 (s, 2 H, CH_2_), 4.56 (s, 2 H, CH_2_), 5.11 (s, 2 H, CH_2_), 7.03 (d, *J*=7.8 Hz, 1 H, Ar), 7.09 (d, *J*=7.8 Hz, 1 H, Ar), 7.32 (d, *J*=8.1 Hz, 2 H, Ar), 7.75 ppm (d, *J*=8.1 Hz, 2 H, Ar); ^13^C NMR (CDCl_3_, 75 MHz, TMS): *δ*=11.2, 21.5, 50.5, 52.9, 68.3, 71.6, 120.6, 120.9, 126.8, 127.5, 129.9, 133.4, 136.3, 136.5, 139.3, 143.8 ppm; IR (CH_2_Cl_2_) 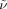
=2956, 2923, 2855, 1597, 1493, 1465, 1345, 1163, 1098, 680 cm^−1^; MS (ESI): *m*/*z*: 342.1 [*M*+H]^+^; HRMS (ESI): *m*/*z* [*M*+H]^+^ calcd for C_19_H_19_NO_3_S: 341.1086, found: 341.1083.

**Ruthenium-catalyzed intramolecular cross-metathesis of diynes:** Substrate **3** (0.2 mmol), first-generation Grubbs catalyst (10 mol %) and 1,2-dichloroethane (2.0 mL) was added to a flame-dried Schlenk tube, and the resulting solution was stirred at 70 °C for 12 h. The reaction mixture was concentrated in vacuo, and the residue was purified by flash silica gel column chromatography (pentane/EtOAc, 10:1–4:1).

**Compound 4 a:** Colorless oil (54 mg, 68 %): ^1^H NMR (CDCl_3_, 400 MHz, TMS): *δ*=0.51 (dd, *J*_1_=8.0 Hz, *J*_2_=6.4 Hz, 2 H, CH_2_), 0.93 (dd, *J*_1_=8.0 Hz, *J*_2_=6.4 Hz, 2 H, CH_2_), 2.44 (s, 3 H, CH_3_), 4.06 (t, *J*=4.0 Hz, 2 H, CH_2_), 4.26 (t, *J*=4.0 Hz, 2 H, CH_2_), 4.75 (d, *J*=2.0 Hz, 2 H, CH_2_), 5.08 (d, *J*=18.0 Hz, 1 H, =CH_2_), 5.21 (d, *J*=10.8 Hz, 1 H, =CH_2_), 5.76 (t, *J*=2.0 Hz, 1 H, =CH), 6.48 (dd, *J*_1_=18.0 Hz, *J*_2_=10.8 Hz, 1 H, =CH), 7.34 (d, *J*=8.0 Hz, 2 H, Ar), 7.72 ppm (d, *J*=8.0 Hz, 2 H, Ar); ^13^C NMR (CDCl_3_, 100 MHz, TMS): *δ*=10.3, 21.5, 54.4, 57.4, 71.9, 73.4, 117.6, 126.87, 126.94, 127.4, 128.4, 129.9, 133.8, 134.9, 143.8 ppm; IR (CH_2_Cl_2_): 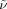 =2927, 2858, 1597, 1454, 1345, 1163, 1095, 817 cm^−1^; MS (ESI): *m*/*z* 344.1 [*M*+H]^+^; HRMS (ESI): *m*/*z* [*M*+H]^+^ calcd for C_19_H_21_NO_3_S: 343.1242, found: 343.1248.

## Supplementary material

As a service to our authors and readers, this journal provides supporting information supplied by the authors. Such materials are peer reviewed and may be re-organized for online delivery, but are not copy-edited or typeset. Technical support issues arising from supporting information (other than missing files) should be addressed to the authors.
