# Supplementary material for: Ruthenium-Catalyzed Intramolecular [2+2+2] Cycloaddition and Tandem Cross-Metathesis of Triynes and Enediynes
Source: ChemistryOpen. 2013 Feb 19;2(2):63–8. doi: 10.1002/open.201300002 (PMC3646433; doi:10.1002/open.201300002)
Supplement: Supplementary file 1 [file open0002-0063-SD1.pdf]

ChemistryOPEN

Supporting Information

© 2013 The Authors. Published by Wiley-VCH Verlag GmbH & Co. KGaA, Weinheim

Ruthenium-Catalyzed Intramolecular [2+2+2] Cycloaddition and Tandem Cross-Metathesis of Triynes and Enediynes

Wei Yuan, Yin Wei, and Min Shi*[a]

open_201300002_sm_miscellaneous_information.pdf

CONTENTS

1. General Remarks…………………………….…………………………………....S2

2. Figure SI-1………………………………………………………………........S2

3. Deuterium labeling experiment…………………………..……………………....S3-S6

4. Representative procedure for the preparation of substrates **1a-1c**, **3a** and **3b**….S6-S14

5. Representative procedure for the preparation of substrates **1h** and **1i**………….S14-S19

6. Representative procedure for the preparation of other substrates………………S19-S48

7. Representative procedure for the preparation of substrates [D]-**1e** and [D]-**3h**...S49-S50

8. Representative procedure for the ruthenium-catalyzed intramolecular [2+2+2] cycloaddition of triynes…..……………………………………………………………………..S51-S64

9. Table SI-1………………………………………………………………..…S65

10. Representative procedure for the ruthenium-catalyzed intramolecular cross-metathesis of diynes…………………………………..……………………………….........S65-S82

11. Scheme SI-1…………………………………………………………………S83

12. References……………………………………………………………………..S84

General Remarks. ^{1}H and ^{13}C NMR spectra were recorded at the 300 and 75 MHz or the 400 and 100 MHz, respectively. Mass and HRMS spectra were recorded by EI or ESI method. Organic solvents used were dried by standard methods when necessary. Satisfactory CHN microanalyses were obtained with an analyzer. Commercially obtained reagents were used without further purification. All these reactions were monitored by TLC with silica gel coated plates. Flash column chromatography was carried out using silica gel at increased pressure. Compounds **SI-5a**,[1] **SI-5c**[1] and **1g**[2] were synthesized following a slightly modified literature procedure.[1]

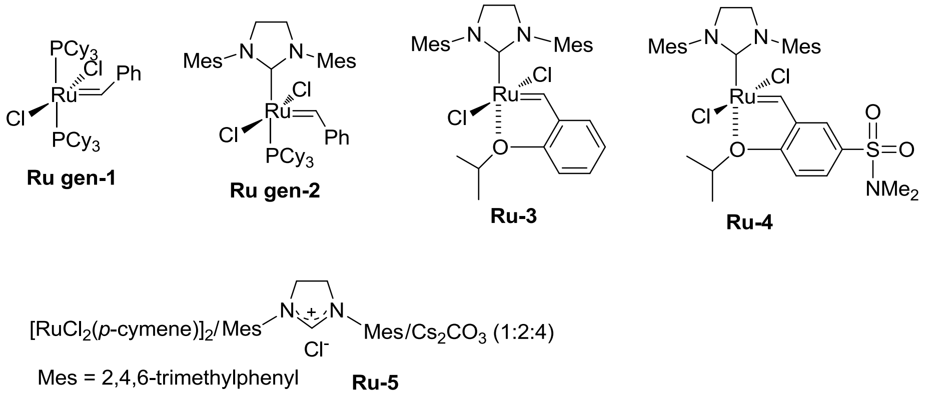

Figure SI-1. Catalysts used in intramolecular cycloaddition and tandem cross-metathesis reactions of triynes and enediynes.

Deuterium Labeling Experiment:

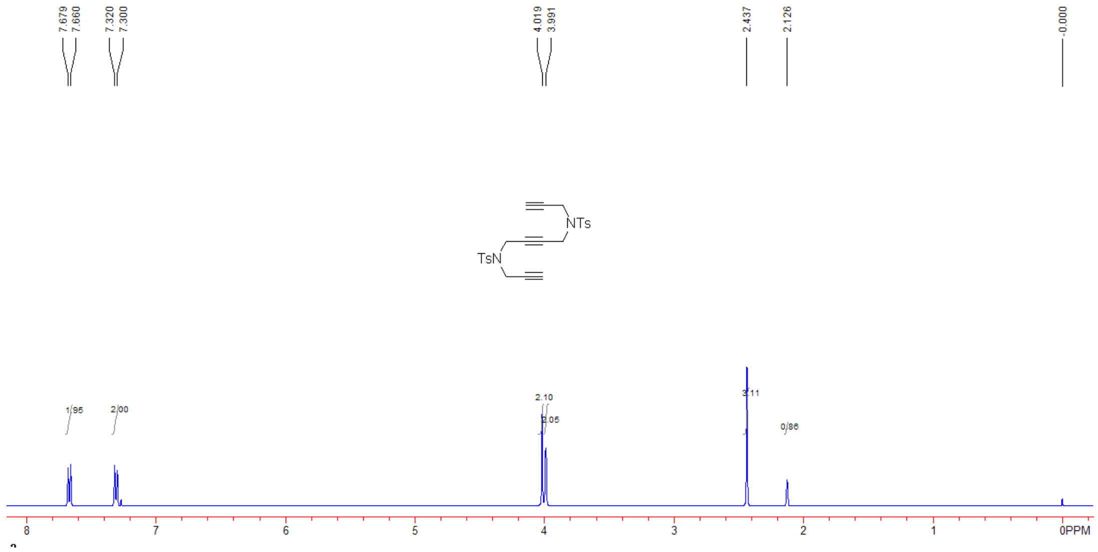

Figure SI-2. ^{1}H NMR spectrum of **1e**

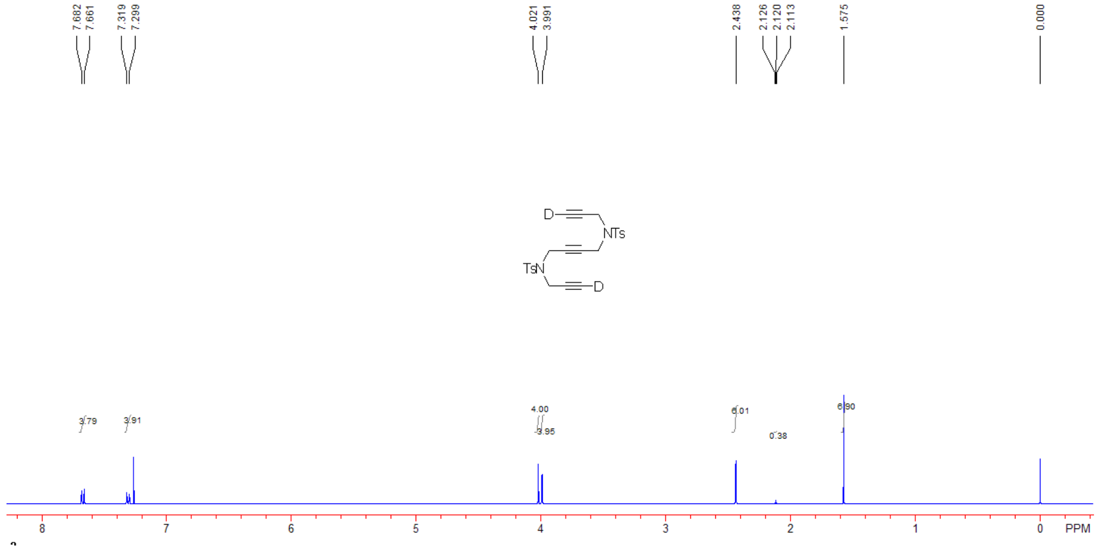

Figure SI-3. ^{1}H NMR spectrum of [D]-**1e**

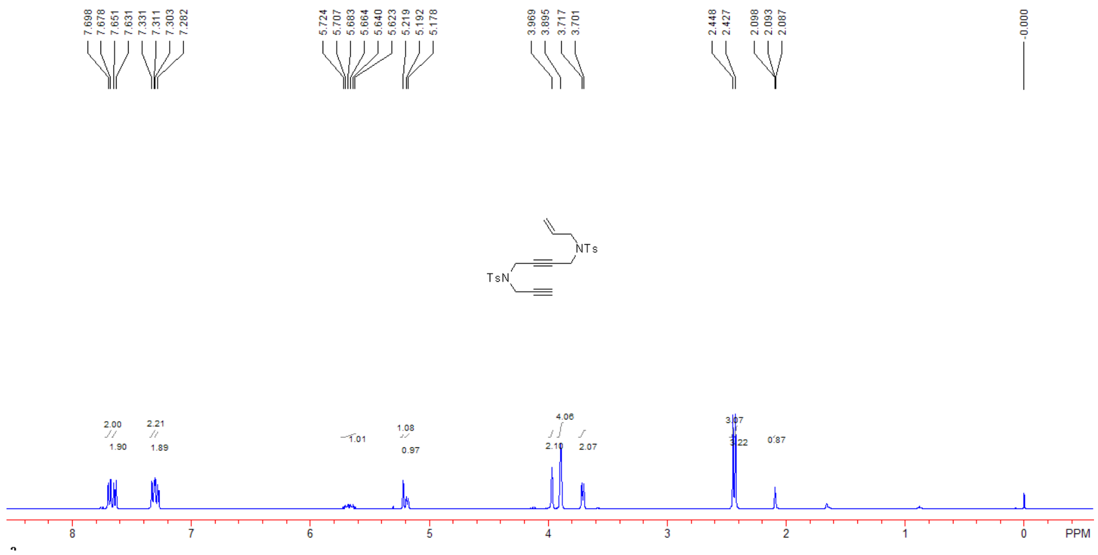

Figure SI-4. ^{1}H NMR spectrum of **3h**

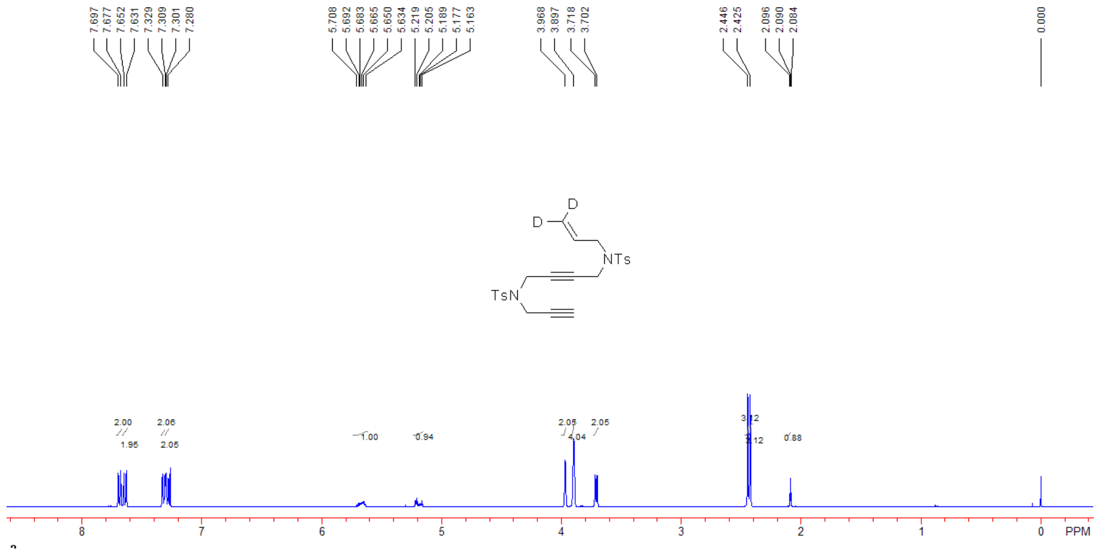

Figure SI-5. ^{1}H NMR spectrum of [D]-**3h**

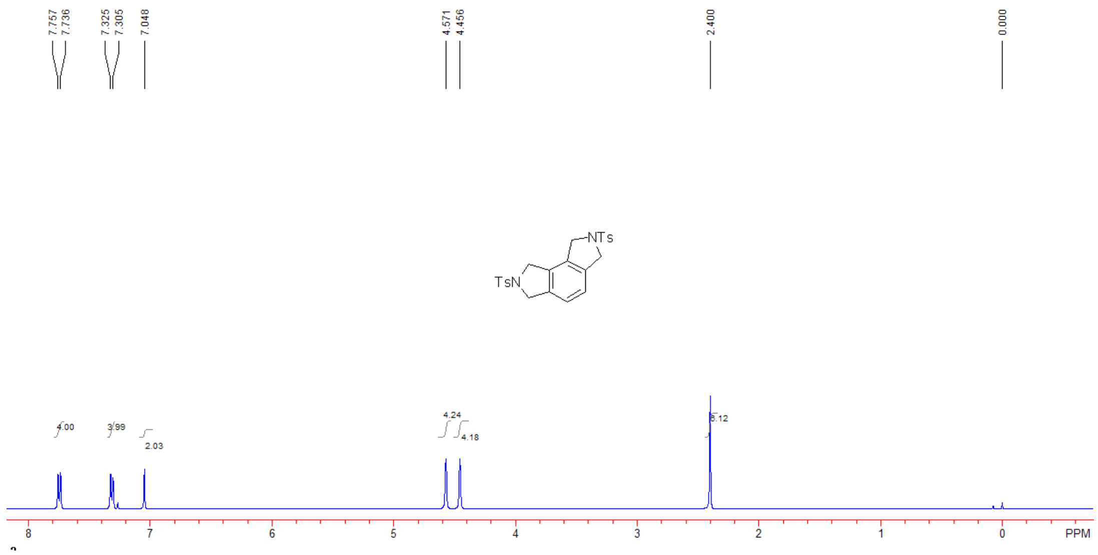

Figure SI-6. ^{1}H NMR spectrum of **2e**

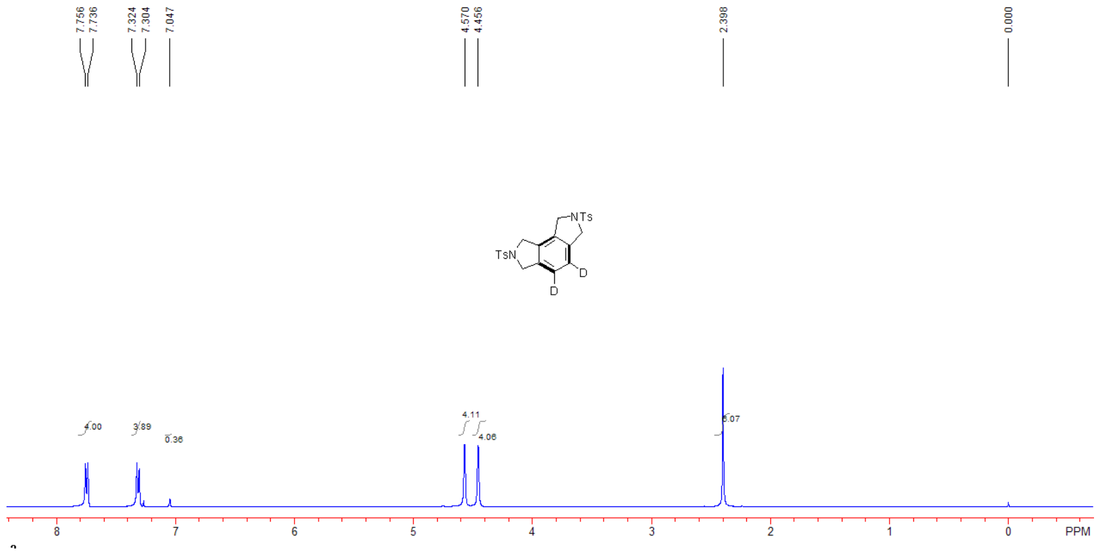

Figure SI-7. ^{1}H NMR spectrum of [D]-**2e**

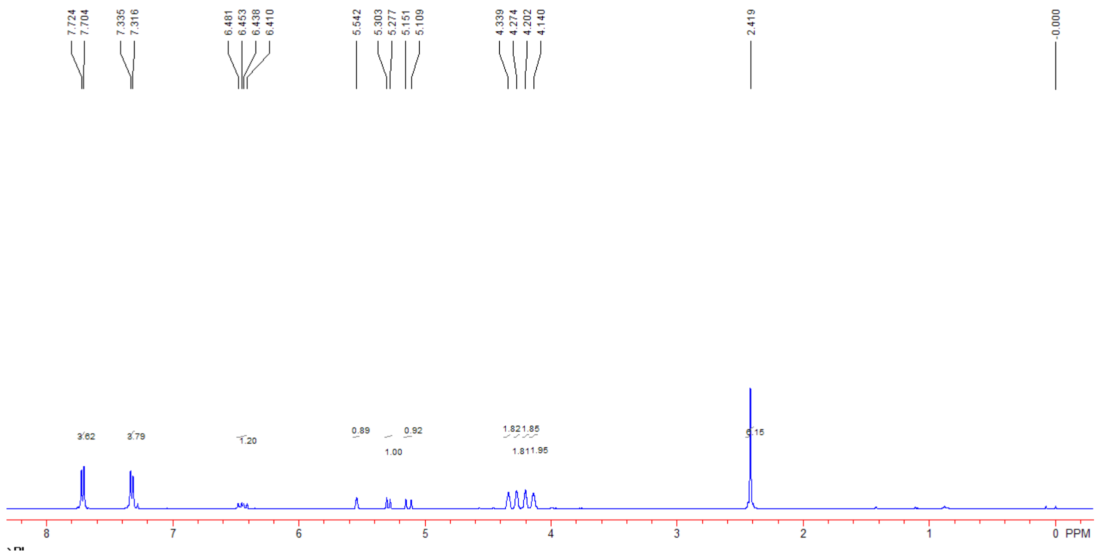

Figure SI-8. ^{1}H NMR spectrum of **4h**

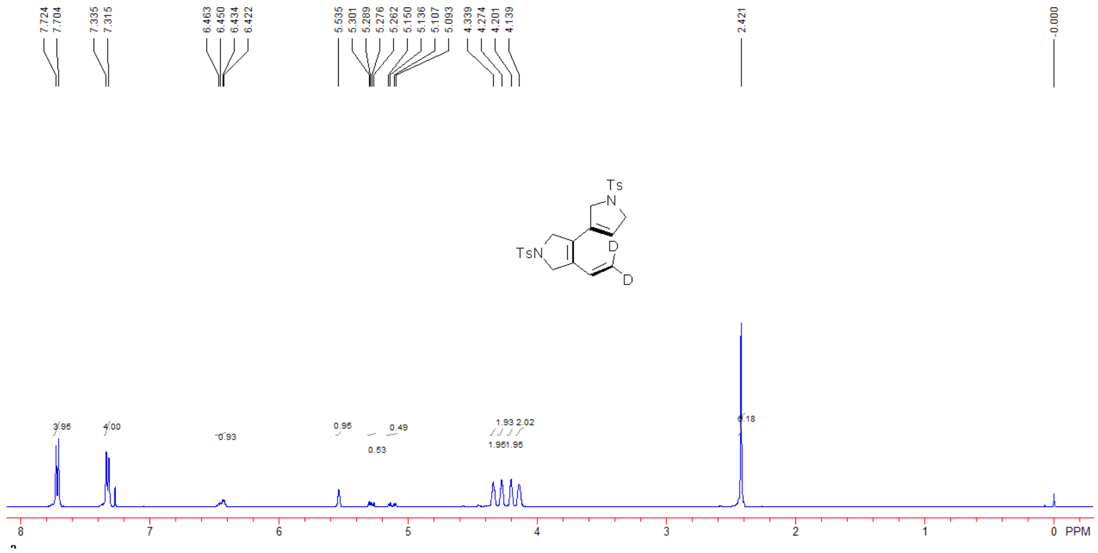

Figure SI-9. ^{1}H NMR spectrum of [D]-**4h**

Representative procedure for the preparation of substrates 1a, 1c, 1g, 3a, 3c:

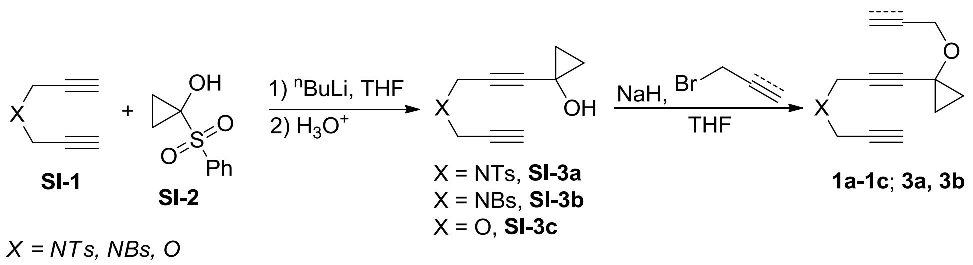

Synthesis of SI-3:

The substrates were synthesized following a slightly modified literature procedure.[3a] To the solution of di(prop-2-ynyl) derivative **SI-1** (20 mmol) in THF (30 mL) was added ^{n}BuLi (22 mmol, 2.5 M in THF) within 20 min at -78 ^{o}C under argon. The resulting solution was allowed to stir at -78 ^{o}C for 2 h before a solution of **SI-2**[3b] (10 mmol) in THF (10 mL) was added into the above mixture. Consequently, the reaction mixture was allowed to warm up to room temperature and was stirred for 4 h. Then, saturated NH_4Cl solution was added to quench the reaction. Extracted with ethyl ether, dried over anhydrous Na_2SO_4, filtered, the organic phase was purified by flash column chromatography on silica gel to give the desired products (PE/EA: 4:1~2:1).

Synthesis of 1a-1c, 3a and 3b:

To the solution of NaH (3.0 mmol) in THF (20 mL) was added a solution of **SI-3** (2.0 mmol) in THF (5.0 mL) at 0 ^{o}C. The resulting solution was allowed to stir at 0 ^{o}C for 0.5 h before propargyl bromide (3.0 mmol) or allyl bromide (3.0 mmol) was added dropwise into the above mixture. Consequently, the reaction mixture was allowed to warm up to room temperature and was stirred for 12 h. Then, saturated NH_4Cl solution was added to quench the reaction. Extracted with ethyl ether, dried over anhydrous Na_2SO_4, filtered, the organic phase was purified by flash column chromatography on silica gel to give the desired products (PE/EA: 20:1).

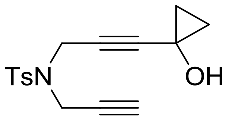

Compound SI-3a. 2.303 g, yield: 76%, light yellow oil. ^{1}H NMR ($CDCl_3$, 400 MHz, TMS) δ 0.73 (dd, J_1 = 8.0 Hz, J_2 = 4.8 Hz, 2H, CH_2), 0.97 (dd, J_1 = 8.0 Hz, J_2 = 4.8 Hz, 2H, CH_2), 2.17 (t, J = 2.4 Hz, 1H, ≡CH), 2.43 (s, 3H, CH_3), 2.66 (brs, 1H, OH), 4.13 (d, J = 2.4 Hz, 2H, CH_2), 4.19 (s, 2H, CH_2), 7.31 (d, J = 8.0 Hz, 2H, Ar), 7.71 (d, J = 8.0 Hz, 2H, Ar). ^{13}C NMR ($CDCl_3$, 100 MHz, TMS) δ 17.0, 21.5, 36.3, 36.6, 45.1, 74.0, 74.4, 76.3, 87.7, 127.8, 129.5, 135.2, 143.9. IR (CH_2Cl_2) ν 3297, 2963, 2342, 2360, 1574, 1472, 1351, 1161, 1192, 1009, 941, 801 cm^{-1}. MS (ESI) m/e 321.1 (M^++NH_4). HRMS (ESI) calcd. for $C_{16}H_{17}NO_3S$: 303.0929,

Found: 303.0920.

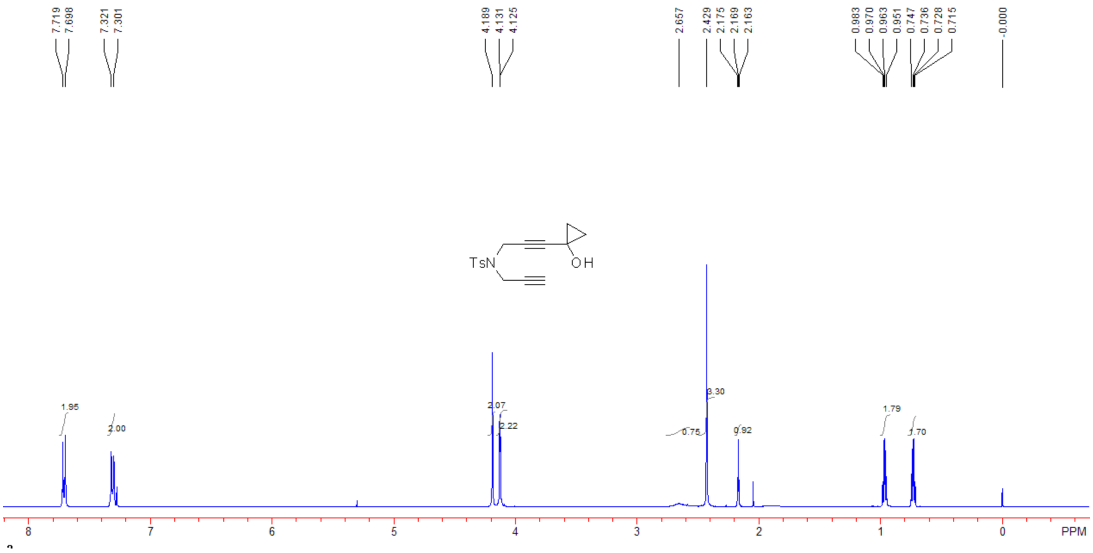

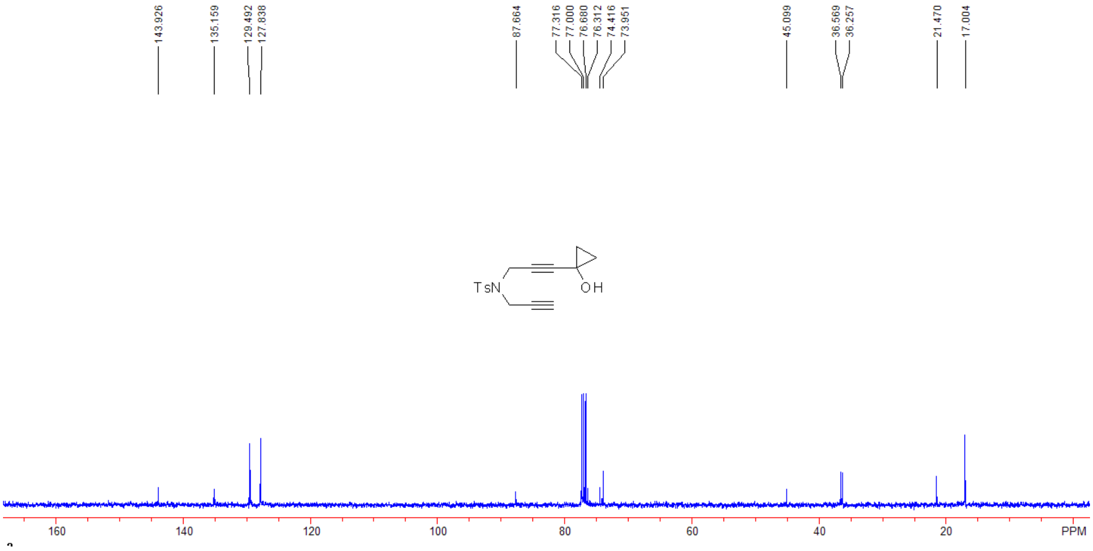

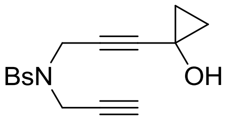

Compound SI-3b. 2.275 g, yield: 62%, light yellow oil. ^{1}H NMR ($CDCl_3$, 400 MHz, TMS) δ 0.77 (s, 2H, CH_2), 1.01 (s, 2H, CH_2), 2.72 (brs, 1H, OH), 4.14 (s, 2H, CH_2), 4.19 (s, 2H, CH_2), 7.67 (d, J = 8.8 Hz, 2H, Ar), 7.71 (d, J = 8.8 Hz, 2H, Ar). ^{13}C NMR ($CDCl_3$, 100 MHz, TMS) δ 17.2, 36.4, 36.7, 45.1, 74.1, 74.3, 75.9, 88.0, 128.1, 129.3, 132.2, 137.2. IR (CH_2Cl_2) ν 3488, 3292, 3089, 1574, 1472, 1390, 1350, 1232, 1161, 1092, 893, 761 cm^{-1}. MS (ESI) m/e 390.0 (M^++Na). HRMS (ESI) calcd. for $C_{15}H_{14}BrNNaO_3S$: 389.9770, Found: 389.9768.

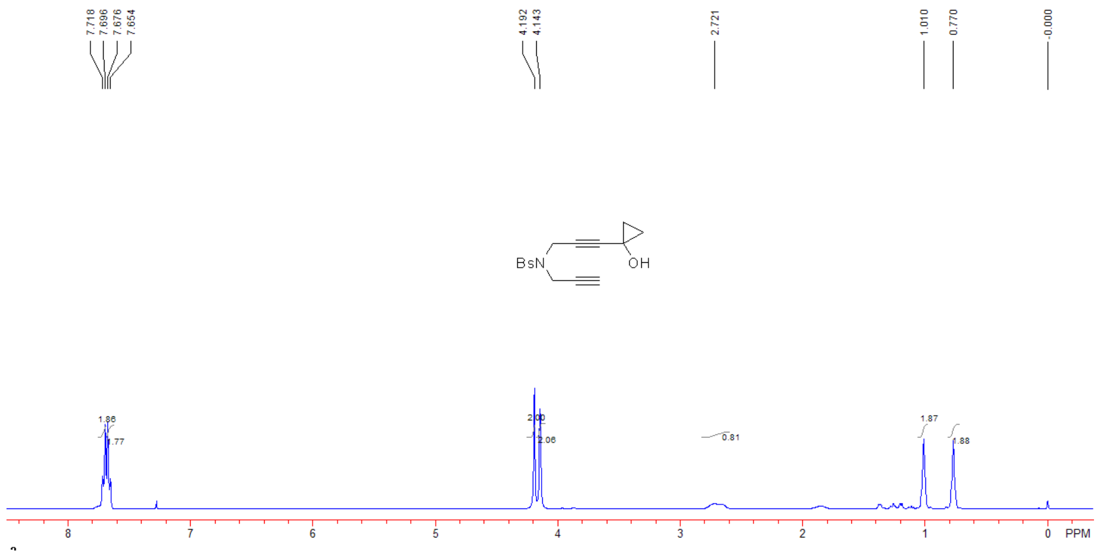

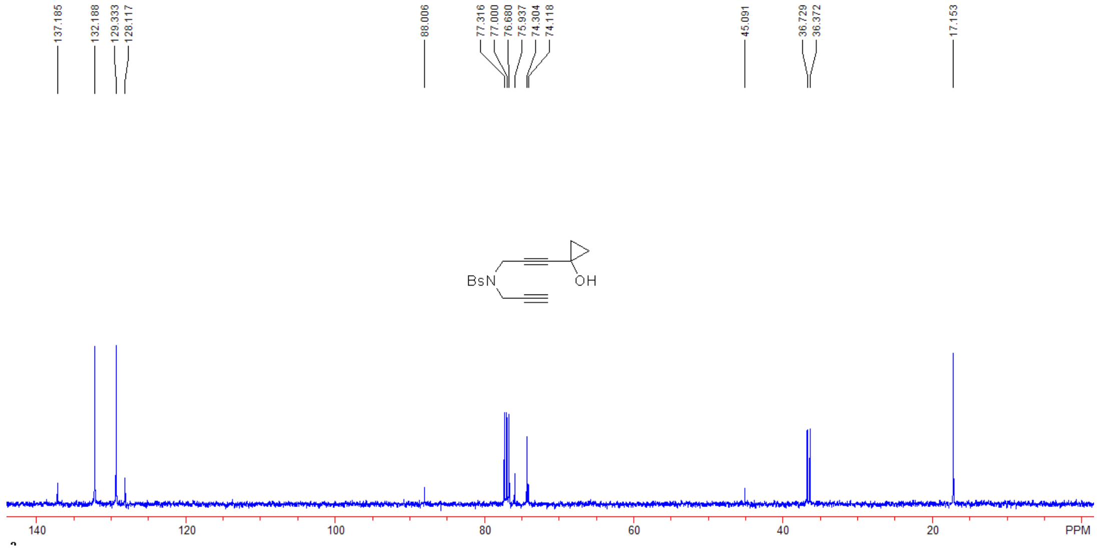

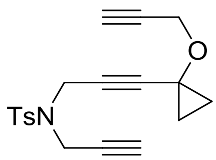

Compound 1a. 0.382 g, yield: 56%, light yellow oil. ^{1}H NMR ($CDCl_3$, 400 MHz, TMS) δ 0.73 (dd, J_1 = 8.0 Hz, J_2 = 4.2 Hz, 2H, CH_2), 1.08 (dd, J_1 = 8.0 Hz, J_2 = 4.2 Hz, 2H, CH_2), 2.18 (t, J = 2.4 Hz, 1H, ≡CH), 2.43 (t, J = 2.4 Hz, 1H, ≡CH), 2.44 (s, 3H, CH_3), 4.09 (d, J = 2.4 Hz, 2H, CH_2), 4.14 (d, J = 2.4 Hz, 2H, CH_2), 4.23 (s, 2H, CH_2), 7.32 (d, J = 8.0 Hz, 2H, Ar), 7.71 (d, J = 8.0 Hz, 2H, Ar). ^{13}C NMR ($CDCl_3$, 100 MHz, TMS) δ 15.6, 21.5, 36.4, 36.6, 51.9, 55.9, 74.0, 74.4, 76.3, 76.7, 79.6, 84.7, 127.8, 129.6, 135.2, 144.0. IR (CH_2Cl_2) ν 3286, 1597, 1440, 1349, 1329, 1229, 1159, 1093, 1047, 893, 662 cm^{-1}. MS (ESI) m/e 359.1

(M^++NH_4). HRMS (ESI) calcd. for $C_{19}H_{19}NO_3S$: 341.1086, Found: 341.1095.

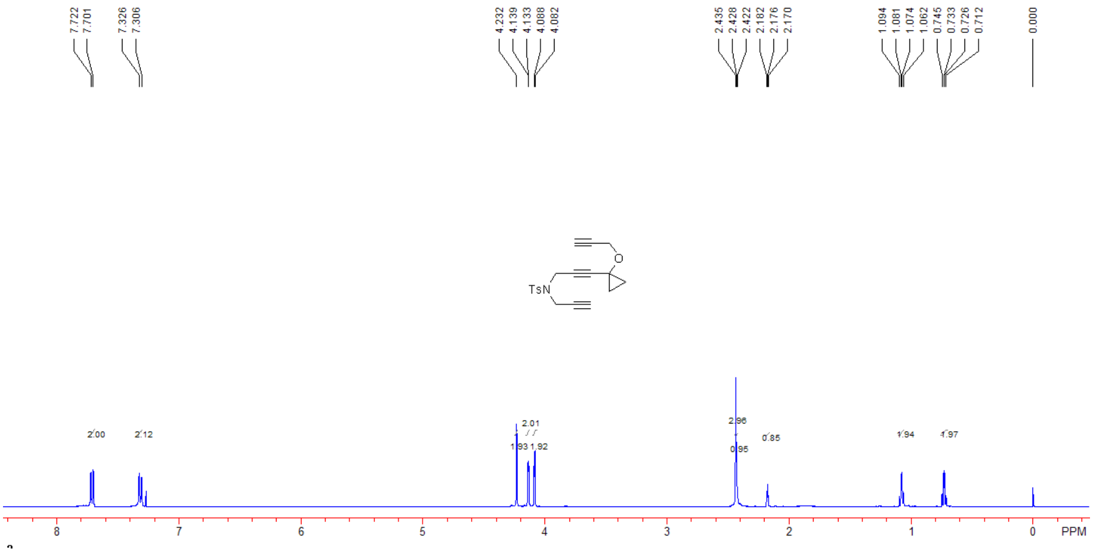

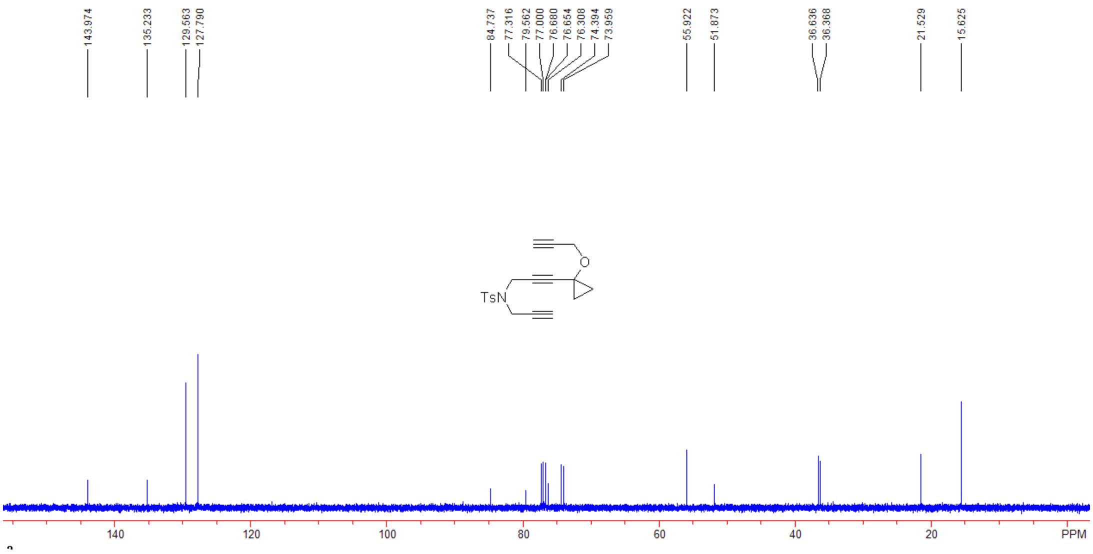

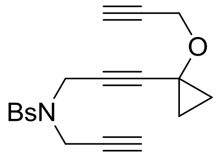

Compound 1b. 0.211 g, yield: 26%, light yellow oil. ^{1}H NMR ($CDCl_3$, 400 MHz, TMS) δ 0.74-0.77 (m, 2H, CH_2), 1.10-1.13 (m, 2H, CH_2), 2.21 (t, *J* = 2.4 Hz, 1H, ≡CH), 2.46 (t, *J* = 2.4 Hz, 1H, ≡CH), 4.12 (d, *J* = 2.4 Hz, 2H, CH_2), 4.15 (d, *J* = 2.4 Hz, 2H, CH_2), 4.23 (s, 2H, CH_2), 7.66-7.72 (m, 4H, Ar). ^{13}C NMR ($CDCl_3$, 100 MHz, TMS) δ 15.6, 36.5, 36.7, 51.7, 55.9, 74.3, 74.5, 75.9, 76.3, 79.4, 85.0, 128.1, 129.2, 132.2, 137.2. IR (CH_2Cl_2) ν 3291, 1575, 1472, 1390, 1353, 1229, 1165, 1093, 1010, 894, 763 cm^{-1}. MS (ESI) m/e 423.0 (M^++NH_4). HRMS

(ESI) calcd. for $C_{18}H_{16}BrNO_3S$: 405.0034, Found: 405.0034.

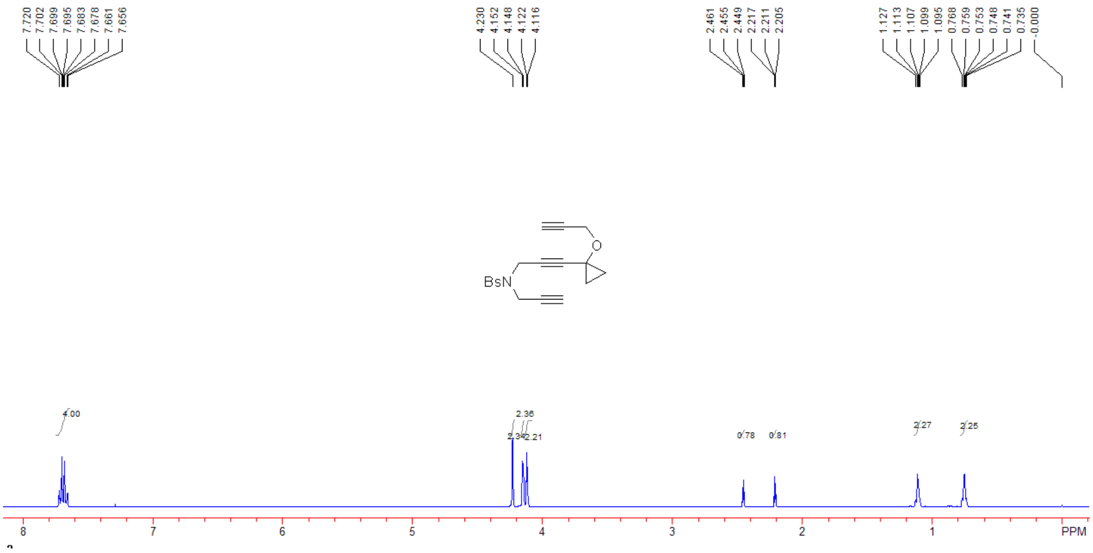

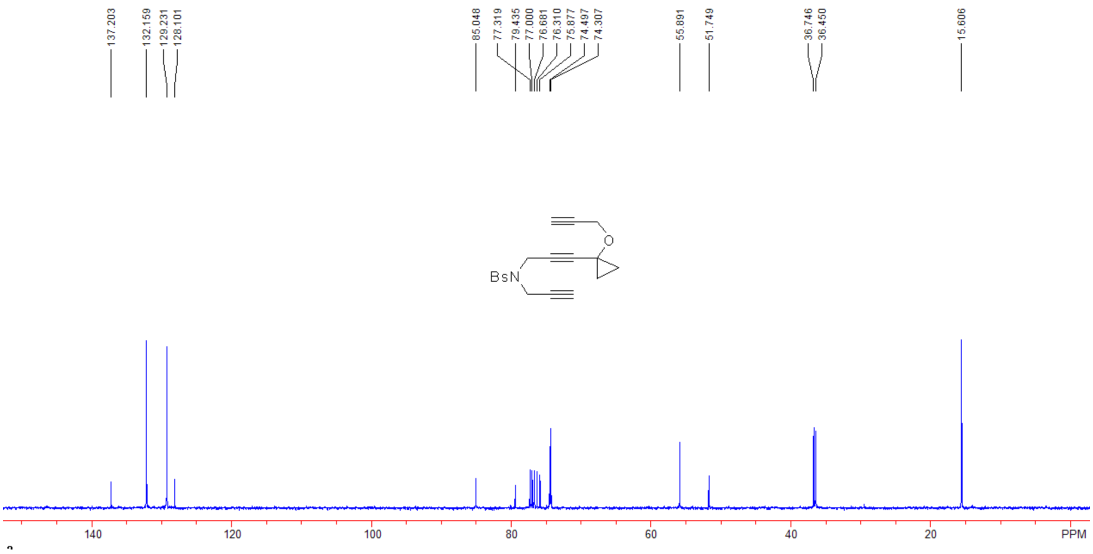

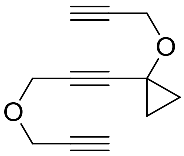

Compound 1c. 0.226 g, yield: 60%, light yellow oil. ^{1}H NMR ($CDCl_3$, 400 MHz, TMS) δ 0.99 (dd, J_1 = 8.0 Hz, J_2 = 4.2 Hz, 2H, CH_2), 1.20 (dd, J_1 = 8.0 Hz, J_2 = 4.2 Hz, 2H, CH_2), 2.44 (t, J = 2.4 Hz, 1H, ≡CH), 2.46 (t, J = 2.4 Hz, 1H, ≡CH), 4.25 (d, J = 2.4 Hz, 2H, CH_2), 4.29 (d, J = 2.4 Hz, 2H, CH_2), 4.31 (s, 2H, CH_2). ^{13}C NMR ($CDCl_3$, 100 MHz, TMS) δ 15.9, 52.1, 56.0, 56.4, 56.8, 74.3, 75.0, 78.8, 79.5, 79.6, 85.9. IR (CH_2Cl_2) ν 3292, 2859, 1442, 1413, 1347, 1228, 1082, 1047, 938 cm^{-1}. MS (ESI) m/e 206.1 (M^{+}+NH_4). HRMS (ESI) calcd. for

$C_{12}H_{12}O_2$: 188.0837, Found: 188.0846.

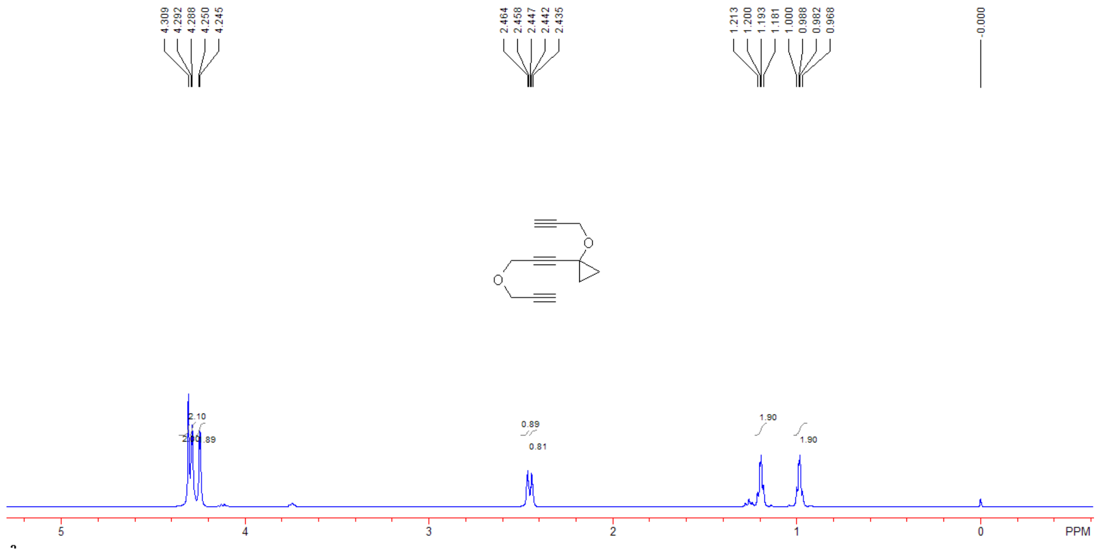

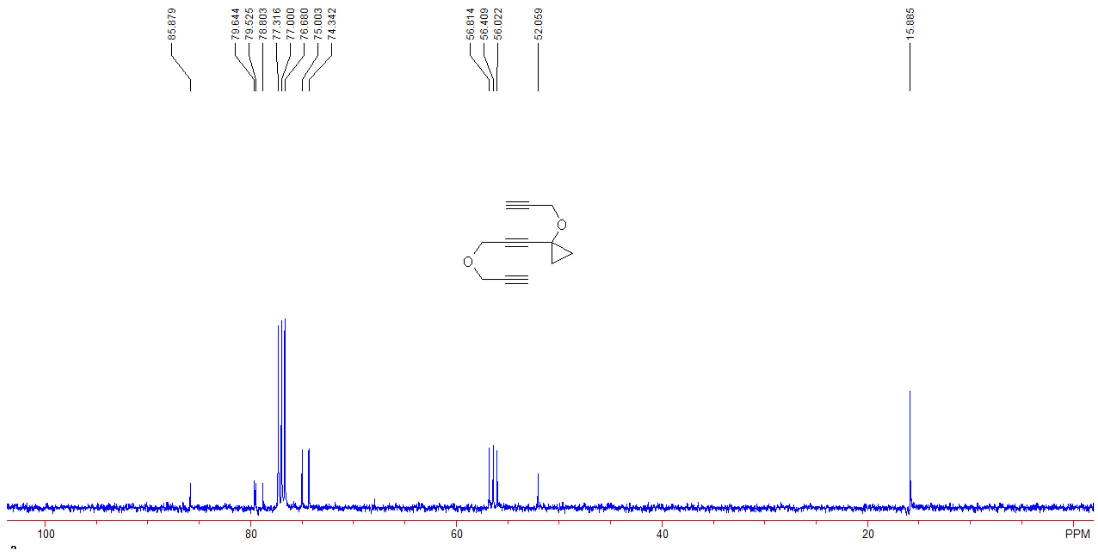

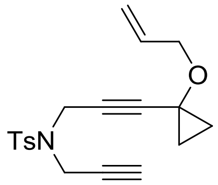

Compound 3a. 0.302 g, yield: 44%, light yellow oil. ^{1}H NMR ($CDCl_3$, 400 MHz, TMS) δ 0.70 (dd, J_1 = 8.0 Hz, J_2 = 1.2 Hz, 2H, CH_2), 0.97 (dd, J_1 = 8.0 Hz, J_2 = 1.2 Hz, 2H, CH_2), 2.16 (t, J = 2.4 Hz, 1H, ≡CH), 2.42 (s, 3H, CH_3), 3.96 (td, J_1 = 5.6 Hz, J_2 = 1.2 Hz, 2H, CH_2), 4.13 (d, J = 2.4 Hz, 2H, CH_2), 4.23 (s, 2H, CH_2), 5.12-5.15 (m, 1H, =CH_2), 5.20-5.25 (m, 1H, =CH_2), 5.80-5.90 (m, 1H, =CH), 7.30 (d, J = 8.0 Hz, 2H, Ar), 7.71 (d, J = 8.0 Hz, 2H, Ar). ^{13}C NMR ($CDCl_3$, 100 MHz, TMS) δ 15.7, 21.5, 36.2, 36.6, 51.3, 69.4, 73.9, 75.5, 76.3, 85.8,

117.0, 127.8, 129.5, 134.2, 135.2, 143.9. IR (CH_2Cl_2) ν 3287, 1597, 1495, 1350, 1330, 1234, 1160, 1093, 1045, 892, 659 cm^{-1}. MS (ESI) m/e 361.2 (M^++NH_4). HRMS (ESI) calcd. for $C_{19}H_{21}NO_3S$: 343.1242, Found: 343.1252.

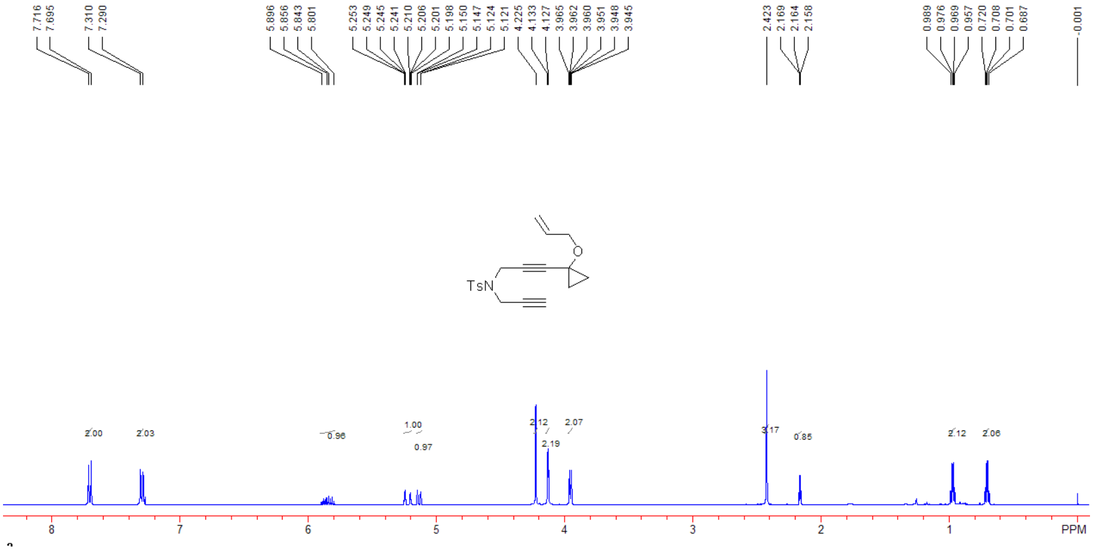

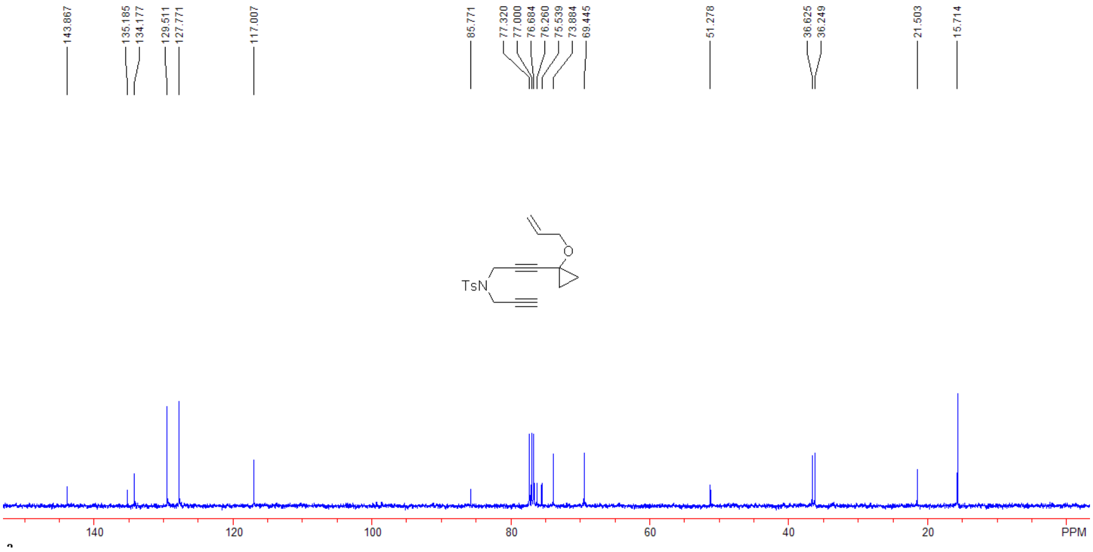

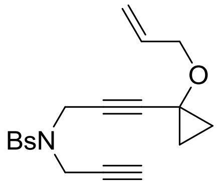

Compound 3b. 0.171 g, yield: 21%, light yellow oil. ^{1}H NMR ($CDCl_3$, 400 MHz, TMS) δ 0.73 (dd, J_1 = 8.0 Hz, J_2 = 1.2 Hz, 2H, CH_2), 1.01 (dd, J_1 = 8.0 Hz, J_2 = 1.2 Hz, 2H, CH_2), 2.22 (s, 1H, ≡CH), 3.95-3.97 (m, 2H, CH_2), 4.14 (d, J = 2.0 Hz, 2H, CH_2), 4.23 (s, 2H, CH_2), 5.13-5.16 (m, 1H, =CH_2), 5.21-5.26 (m, 1H, =CH_2), 5.81-5.91 (m, 1H, =CH), 7.66 (d, J = 8.4

Hz, 2H, Ar), 7.71 (d, *J* = 8.4 Hz, 2H, Ar). ^{13}C NMR ($CDCl_3$, 100 MHz, TMS) δ 15.7, 36.3, 36.7, 51.2, 69.3, 74.2, 75.1, 75.8, 86.0, 116.9, 128.0, 129.2, 132.1, 134.0, 137.1. IR (CH_2Cl_2) ν 3285, 3092, 2926, 1575, 1472, 1390, 1354, 1165, 1069, 792 cm^{-1}. MS (ESI) m/e 425.1 (M^++NH_4). HRMS (ESI) calcd. for $C_{18}H_{18}BrNO_3S$: 407.0191, Found: 407.0195.

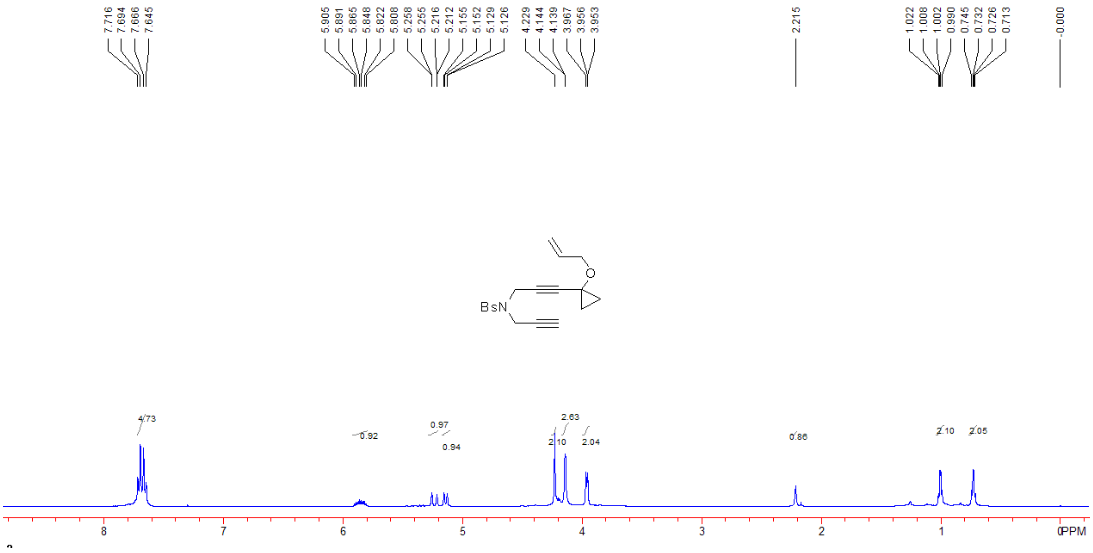

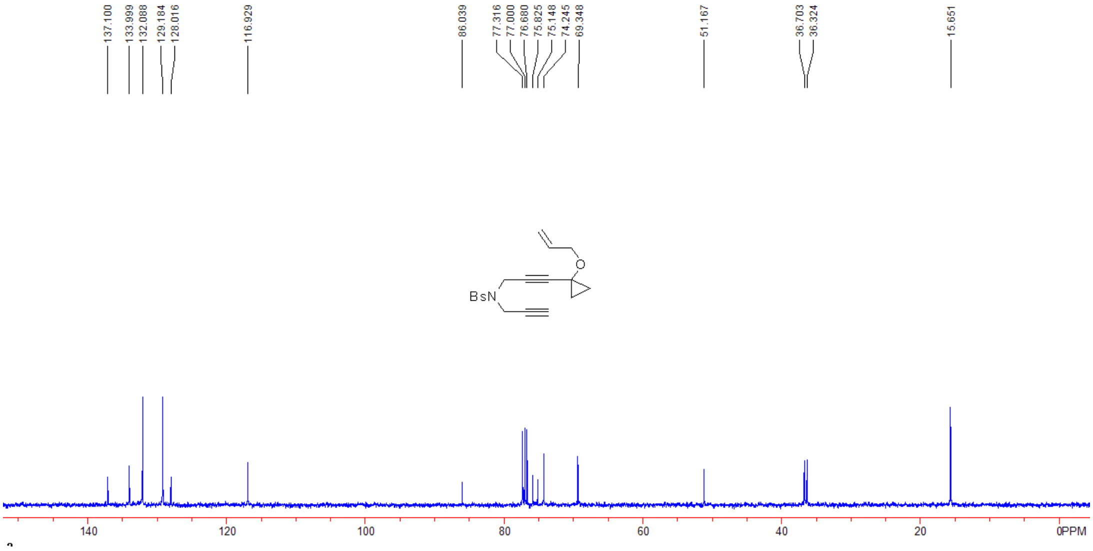

Representative procedure for the preparation of substrates 1h and 1i:

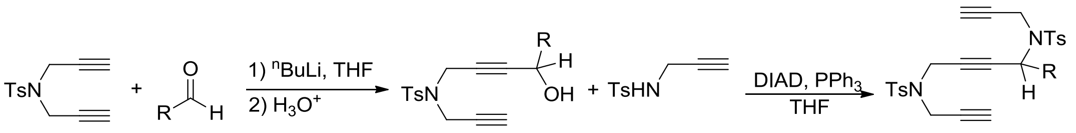

SI-1a *R = Ph, n-Propyl* R = Ph, **SI-4a** R = *n*-Propyl, **SI-4b** R = *n*-Propyl, **1h** R = Ph, **1i**

Synthesis of SI-4:

To the solution of 4-methyl-N,N-di(prop-2-ynyl)benzene sulfonamide **SI-1a** (20 mmol) in THF (30 mL) was added ^{n}BuLi (22 mmol, 2.5 M in THF) within 20 min at -78 ^{o}C under argon. The resulting solution was allowed to stir at -78 ^{o}C for 2 h before aldehyde (22 mmol) was added into the above mixture through disposable syringe. Consequently, the reaction mixture was allowed to warm up to room temperature and was stirred for 4 h. Then, saturated NH_4Cl solution was added to quench the reaction. Extracted with EtOAc, dried over anhydrous Na_2SO_4, filtered, the organic phase was purified by flash column chromatography on silica gel to give the desired products (PE/EA: 4:1~2:1).

Synthesis of 1h and 1i:

To the solution of **SI-4** (10 mmol), 4-methyl-N-(prop-2-ynyl)benzenesulfonamide (12 mmol) and PPh_3 (12 mmol) in THF (20 mL) was added DIAD (12 mmol) at 0 ^{o}C. The resulting solution was allowed to warm up to room temperature and was stirred for 12 h. Then, the organic phase was purified by flash column chromatography on silica gel to give the desired products (PE/EA: 10:1~4:1).

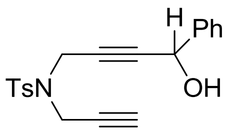

Compound SI-4a. 1.906 g, yield: 27%, light yellow oil. ^{1}H NMR ($CDCl_3$, 400 MHz, TMS) δ 2.15 (t, J = 2.4 Hz, 1H, ≡CH), 2.35 (s, 3H, CH_3), 2.58 (brs, 1H, OH), 4.11 (d, J = 2.4 Hz, 2H, CH_2), 4.25 (s, 2H, CH_2), 5.25 (s, 1H, CH), 7.21 (d, J = 8.0 Hz, 2H, Ar), 7.32-7.34 (m, 5H, Ar), 7.68 (d, J = 8.0 Hz, 2H, Ar). ^{13}C NMR ($CDCl_3$, 100 MHz, TMS) δ 21.4, 36.4, 36.5, 64.1, 74.1, 76.2, 78.8, 85.6, 126.4, 127.8, 128.3, 128.5, 129.5, 134.9, 140.0, 144.0. IR (CH_2Cl_2) ν 3499, 3286, 1597, 1493, 1451, 1347, 1328, 1158, 1092, 896, 660 cm^{-1}. MS (ESI) m/e 371.1 (M^{+}+NH_4). HRMS (ESI) calcd. for $C_{20}H_{19}NO_3S$: 353.1086, Found: 353.1093.

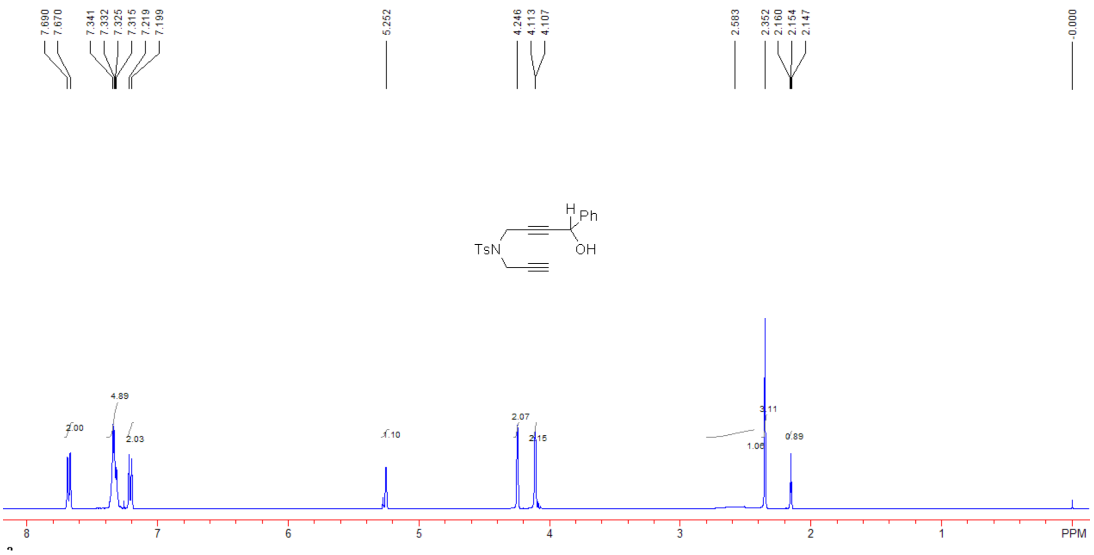

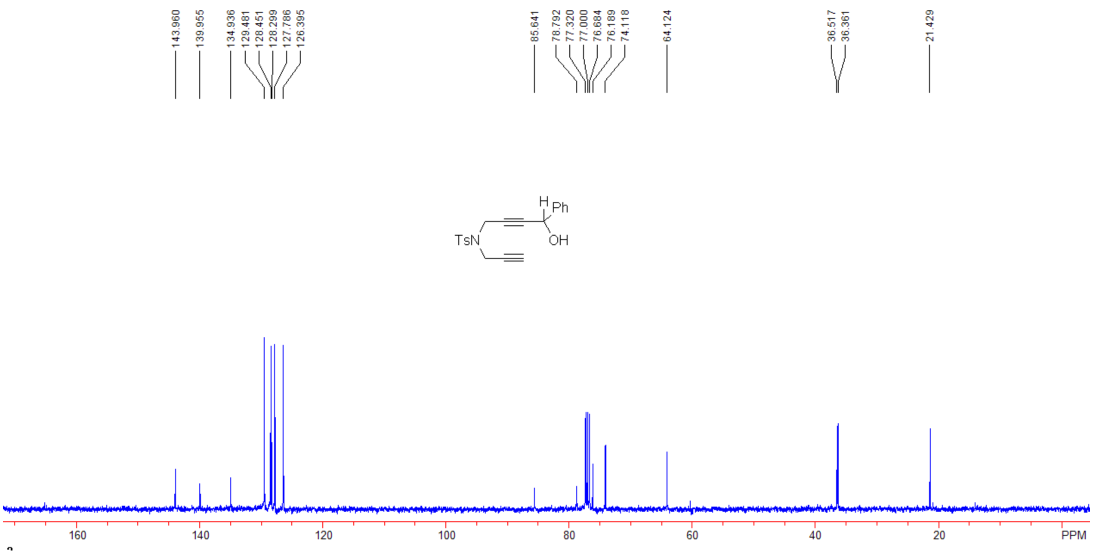

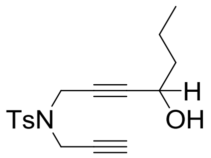

Compound SI-4b. 3.929 g, yield: 59%, light yellow oil. ^{1}H NMR ($CDCl_3$, 300 MHz, TMS) δ 0.88 (t, J = 7.2 Hz, 3H, CH_3), 1.26-1.36 (m, 2H, CH_2), 1.46-1.52 (m, 2H, CH_2), 2.19 (t, J = 1.8 Hz, 1H, ≡CH), 2.42 (s, 3H, CH_3), 4.13 (d, J = 1.8 Hz, 2H, CH_2), 4.19 (s, 3H, CH_2 and CH), 7.31 (d, J = 8.1 Hz, 2H, Ar), 7.71 (d, J = 8.1 Hz, 2H, Ar). ^{13}C NMR ($CDCl_3$, 75 MHz, TMS) δ 13.5, 18.1, 21.3, 36.1, 36.4, 39.3, 61.6, 73.9, 76.2, 76.6, 87.1, 127.7, 129.4, 135.0, 143.9. IR (CH_2Cl_2) ν 3288, 2959, 2927, 2873, 1597, 1494, 1435, 1348, 1328, 1159, 1093, 893, 658 cm^{-1}. MS (ESI) m/e 337.2 (M^++NH_4). HRMS (ESI) calcd. for $C_{17}H_{21}NO_3S$: 319.1242, Found:

319.1246.

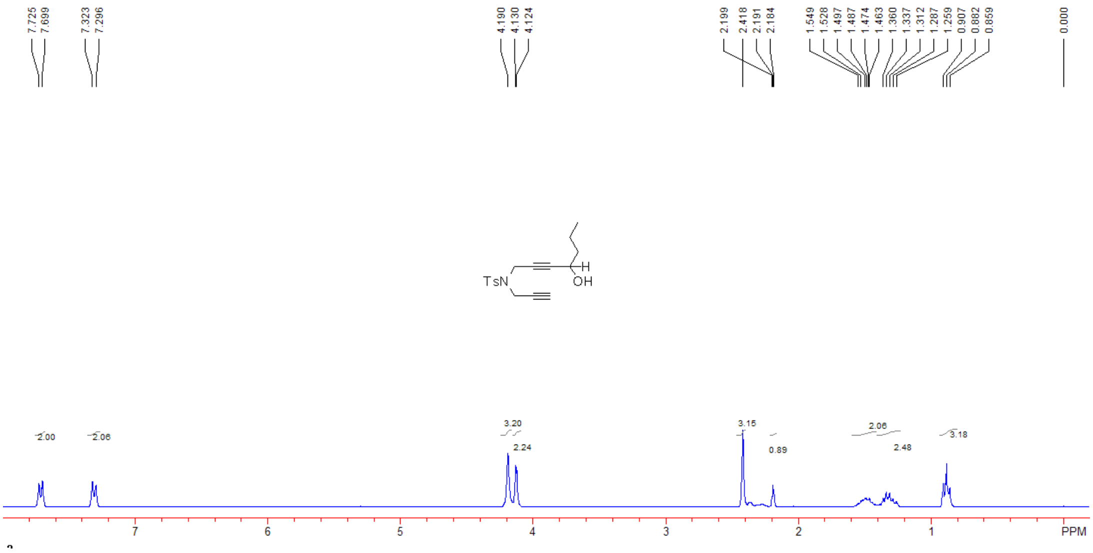

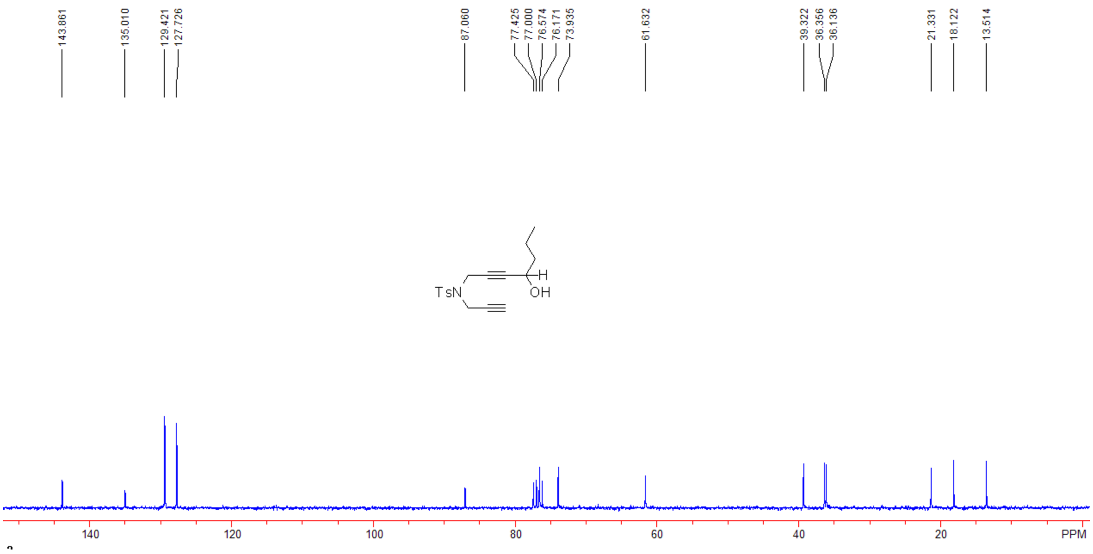

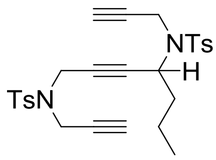

Compound 1h. 0.419 g, yield: 8%, light yellow oil. ^{1}H NMR ($CDCl_3$, 400 MHz, TMS) δ 0.89 (t, J = 7.2 Hz, 3H, CH_3), 1.32-1.42 (m, 3H, CH_2), 1.52-1.59 (m, 1H, CH_2), 1.64-1.73 (m, 2H, CH_2), 2.11 (t, J = 2.4 Hz, 1H, ≡CH), 2.20 (t, J = 2.4 Hz, 1H, ≡CH), 2.44 (s, 6H, CH_3), 3.74 (dd, J_1 = 18.4 Hz, J_2 = 2.4 Hz, 1H, CH_2), 3.85 (dd, J_1 = 12.8 Hz, J_2 = 2.0 Hz, 2H, CH_2), 3.978 (dd, J_1 = 18.4 Hz, J_2 = 2.4 Hz, 1H, CH_2), 3.980 (s, 2H, CH_2), 4.56 (t, J = 8.0 Hz, 1H, CH), 7.297 (d, J = 8.0 Hz, 2H, Ar), 7.303 (d, J = 8.0 Hz, 2H, Ar), 7.65 (d, J = 8.0 Hz, 2H, Ar), 7.70 (d, J =

8.0 Hz, 2H, Ar). ^{13}C NMR ($CDCl_3$, 100 MHz, TMS) δ 19.17, 19.19, 21.5, 21.6, 33.4, 36.0, 36.2, 36.8, 36.9, 50.3, 72.2, 73.9, 76.1, 78.2, 79.3, 82.3, 127.6, 127.7, 129.5, 129.6, 135.2, 136.1, 143.8, 144.1. IR (CH_2Cl_2) ν 2962, 2926, 2850, 1260, 1092, 1018, 798, 749 cm^{-1}. MS (ESI) m/e 528.2 (M^++NH_4). HRMS (ESI) calcd. for $C_{27}H_{30}N_2O_4S_2$: 510.1647, Found: 510.1637.

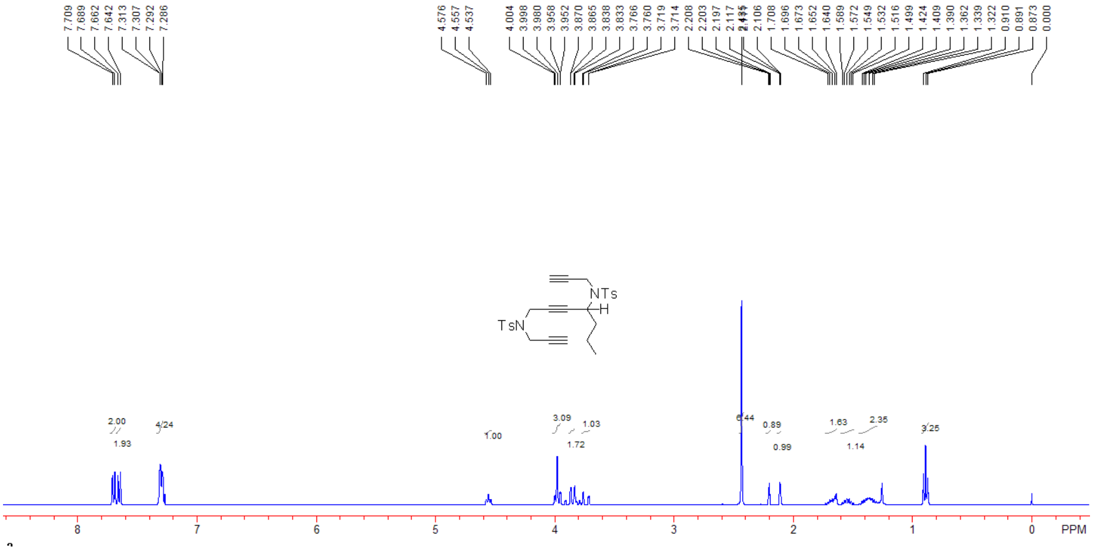

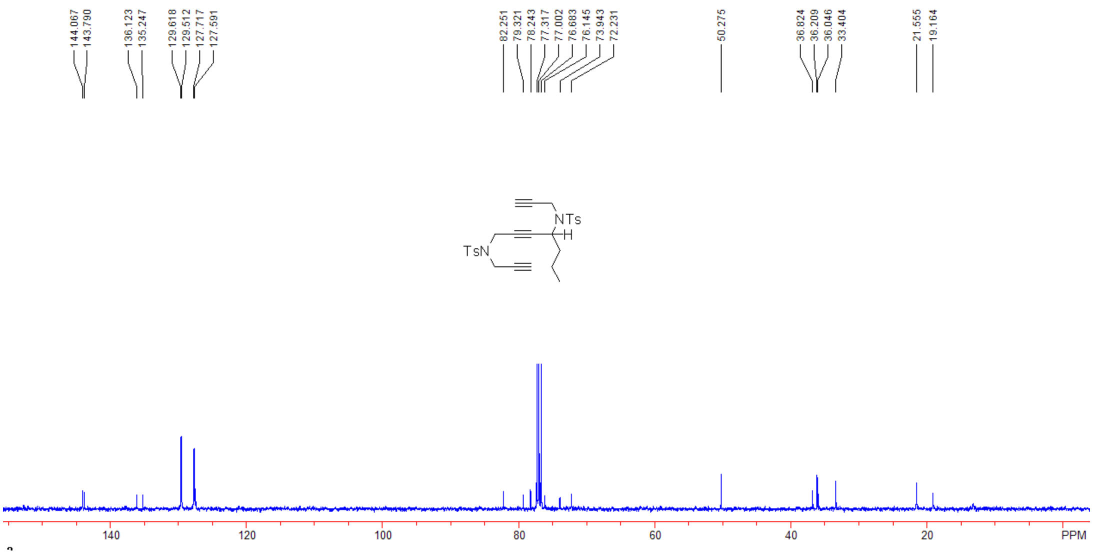

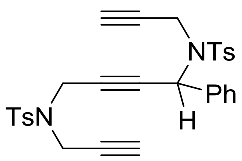

Compound 1i. 1.142 g, yield: 21%, white solid, Mp: 107-109 °C. ^{1}H NMR ($CDCl_3$, 400 MHz, TMS) δ 1.99 (s, 1H, ≡CH), 2.11 (s, 1H, ≡CH), 2.35 (s, 3H, CH_3), 2.47 (s, 3H, CH_3), 3.59 (dd, J_1 = 18.4 Hz, J_2 = 1.6 Hz, 1H, CH_2), 3.82-3.86 (m, 1H, CH_2), 3.93-3.99 (m, 2H, CH_2), 4.06 (s,

2H, CH_2), 5.93 (s, 1H, CH), 7.24 (d, *J* = 8.0 Hz, 2H, Ar), 7.33-7.34 (m, 5H, Ar), 7.42-7.44 (m, 2H, Ar), 7.67 (d, *J* = 8.0 Hz, 2H, Ar), 7.83 (d, *J* = 8.0 Hz, 2H, Ar). ^{13}C NMR ($CDCl_3$, 100 MHz, TMS) δ 21.5, 21.6, 33.6, 36.2, 36.3, 52.8, 72.9, 74.1, 76.2, 78.1, 80.0, 81.1, 127.7, 127.95, 128.00, 128.5, 128.6, 129.4, 129.6, 135.0, 135.2, 136.2, 144.0, 144.1. IR (CH_2Cl_2) ν 3281, 1596, 1493, 1425, 1322, 1154, 1092, 1068, 811, 659 cm^{-1}. MS (ESI) m/e 562.2 (M^++NH_4). HRMS (ESI) calcd. for $C_{30}H_{28}N_2O_4S_2$: 544.1490, Found: 544.1496.

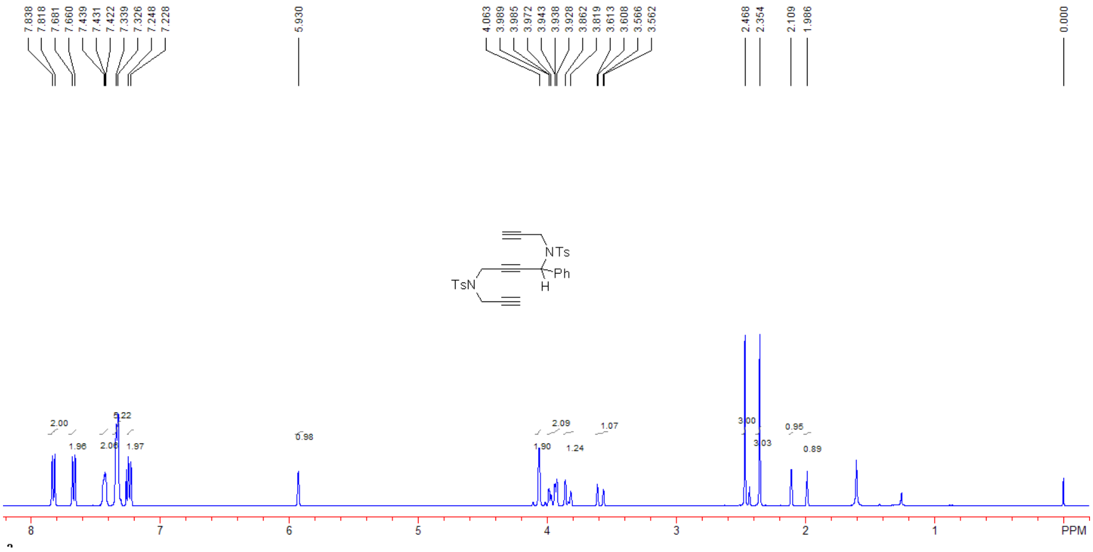

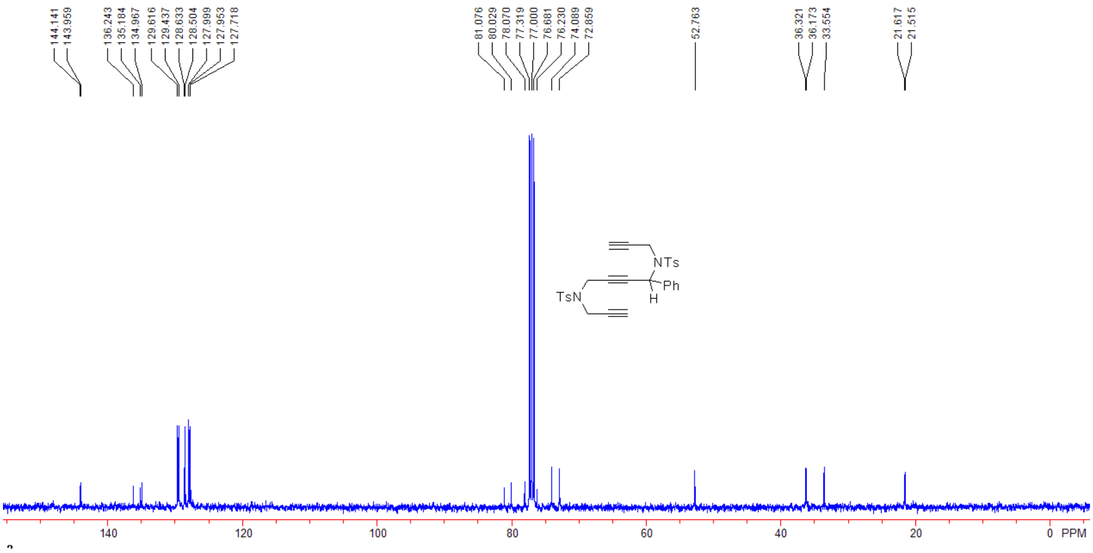

Representative procedure for the preparation of other substrates:

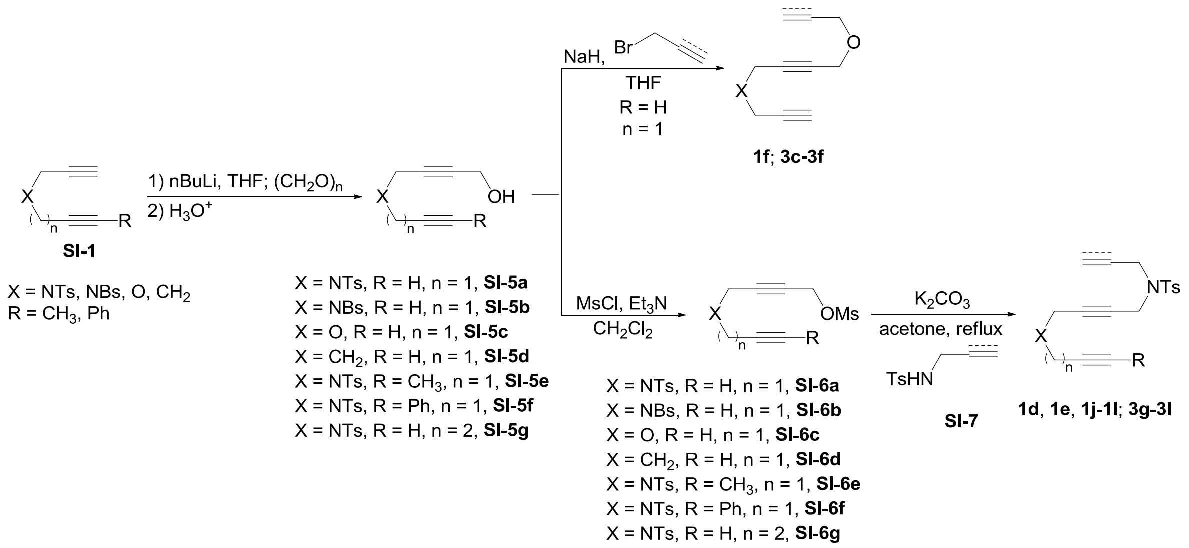

Synthesis of SI-5:

To the solution of **SI-1** (20 mmol) in THF (30 mL) was added ^{n}BuLi (22 mmol, 2.5 M in THF) within 20 min at -78 ^{o}C under argon. The resulting solution was allowed to stir at -78 ^{o}C for 2 h before paraformaldehyde solid (22 mmol) was added into the above mixture directly under argon. Consequently, the reaction mixture was allowed to warm up to room temperature and was stirred for 4 h. Then, saturated NH_4Cl solution was added to quench the reaction. Extracted with EtOAc, dried over anhydrous Na_2SO_4, filtered, the organic phase was purified by flash column chromatography on silica gel to give the desired products **SI-5** (PE/EA: 4:1~2:1).

Synthesis of 1f, 3c-3f:

To the solution of NaH (3.0 mmol) in THF (20 mL) was added a solution of the corresponding **SI-5** (2.0 mmol) in THF (5.0 mL) at 0 ^{o}C. The resulting solution was allowed to stir at 0 ^{o}C for 0.5 h before propargyl bromide (3.0 mmol) or allyl bromide (3.0 mmol) was added dropwise into the above mixture. Consequently, the reaction mixture was allowed to warm up to room temperature and was stirred for 12 h. Then, saturated NH_4Cl solution was added to quench the reaction. Extracted with ethyl ether, dried over Na_2SO_4, filtered, the organic phase was purified by flash column chromatography on silica gel to give the desired products (PE/EA: 20:1).

Synthesis of SI-6:

To the solution of the corresponding **SI-5** (10 mmol) and Et_3N (20 mmol) in CH_2Cl_2 (40 mL) was added MsCl (15 mmol) dropwise at 0 ^oC under argon. The resulting solution was allowed to stir at 0 ^oC for 0.5 h. Then, water was added to quench the reaction. Extracted with CH_2Cl_2, dried over anhydrous Na_2SO_4, filtered, the organic phase was purified by flash column chromatography on silica gel to give the desired products **SI-6** (PE/EA: 4:1~2:1).

Synthesis of 1d, **1e**, **1j-1l**, **3g-3l**:

The corresponding **SI-4** (5 mmol), **SI-7** (5 mmol), K_2CO_3 (7.5 mmol) and the solvent acetone (20 mL) were added into a 50 mL flask. Then, the flask was heated to reflux for 8 h. Finally, the suspension was filtered, the organic phase was purified by flash column chromatography on silica gel to give the desired products (PE/EA: 10:1~4:1).

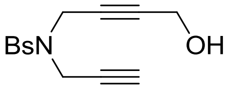

Compound SI-5b. 5.183 g, yield: 76%, light yellow oil. ^{1}H NMR ($CDCl_3$, 400 MHz, TMS) δ 2.10 (brs, 1H, OH), 2.19 (t, *J* = 2.4 Hz, 1H, ≡CH), 4.13 (s, 2H, CH_2), 4.17 (d, *J* = 2.4 Hz, 2H, CH_2), 4.19 (s, 2H, CH_2), 7.66-7.73 (m, 4H, Ar). ^{13}C NMR ($CDCl_3$, 100 MHz, TMS) δ 36.4, 36.6, 50.6, 74.4, 75.8, 77.6, 84.3, 128.1, 129.3, 132.2, 137.0. IR (CH_2Cl_2) ν 3292, 1574, 1472, 1390, 1350, 1330, 1162, 1068, 1009, 895, 760 cm^{-1}. MS (ESI) m/e 360.0 (M^++NH_4). HRMS (ESI) calcd. for $C_{13}H_{12}BrNO_3S$: 340.9721, Found: 340.9724.

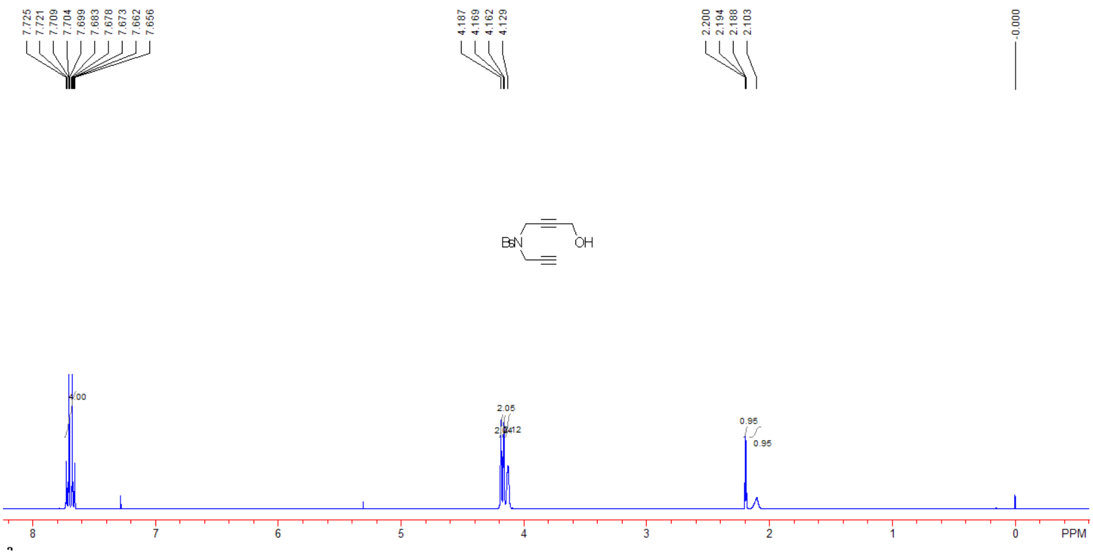

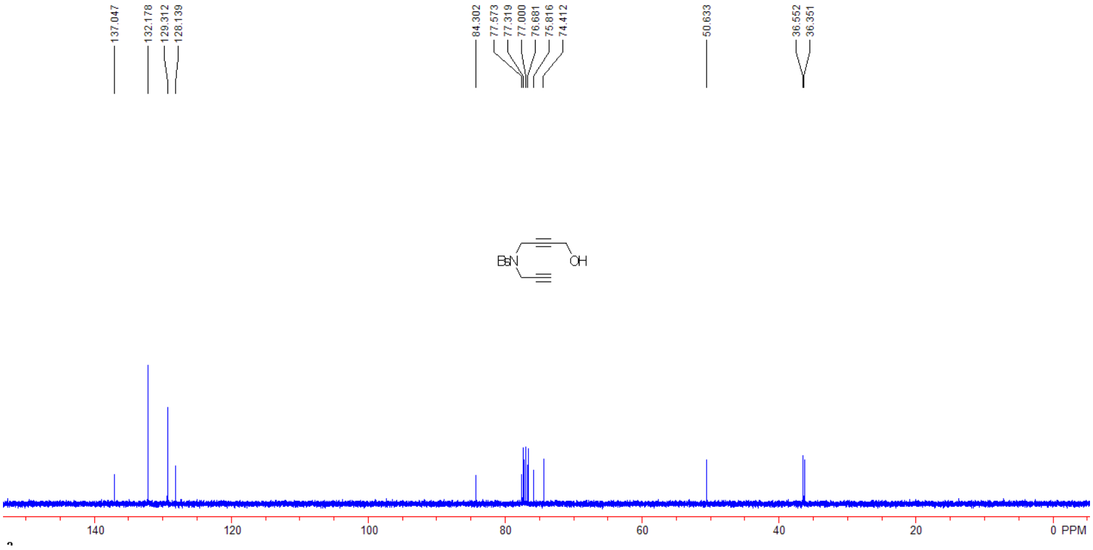

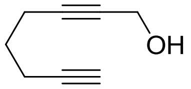

Compound SI-5d. 1.098 g, yield: 45%, colorless oil. ^{1}H NMR ($CDCl_3$, 300 MHz, TMS) δ 1.69-1.78 (m, 2H, CH_2), 1.98 (t, J = 2.4 Hz, 1H, ≡CH), 2.13 (brs, 1H, OH), 2.29-2.39 (m, 4H, CH_2), 4.25 (brs, 2H, CH_2). ^{13}C NMR ($CDCl_3$, 75 MHz, TMS) δ 17.4, 17.7, 27.3, 51.1, 68.9, 79.0, 83.4, 85.0. IR (CH_2Cl_2) ν 3292, 2935, 2905, 2868, 1458, 1432, 1346, 1133, 1009, 981 cm^{-1}. MS (ESI) m/e 123.0 (M^{+}+1). HRMS (ESI) calcd. for $C_8H_{10}NaO$: 145.0624, Found: 145.0624.

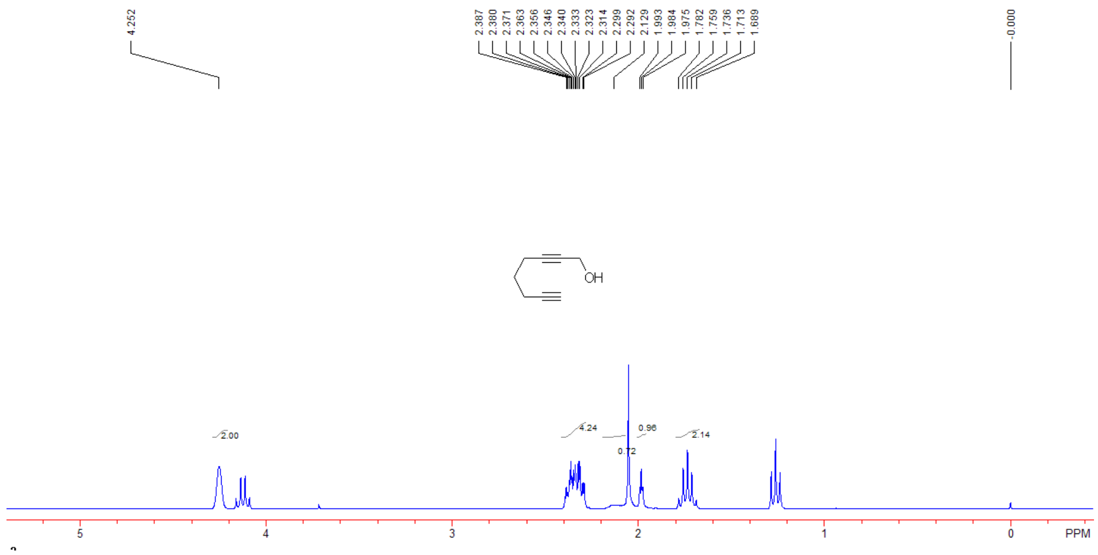

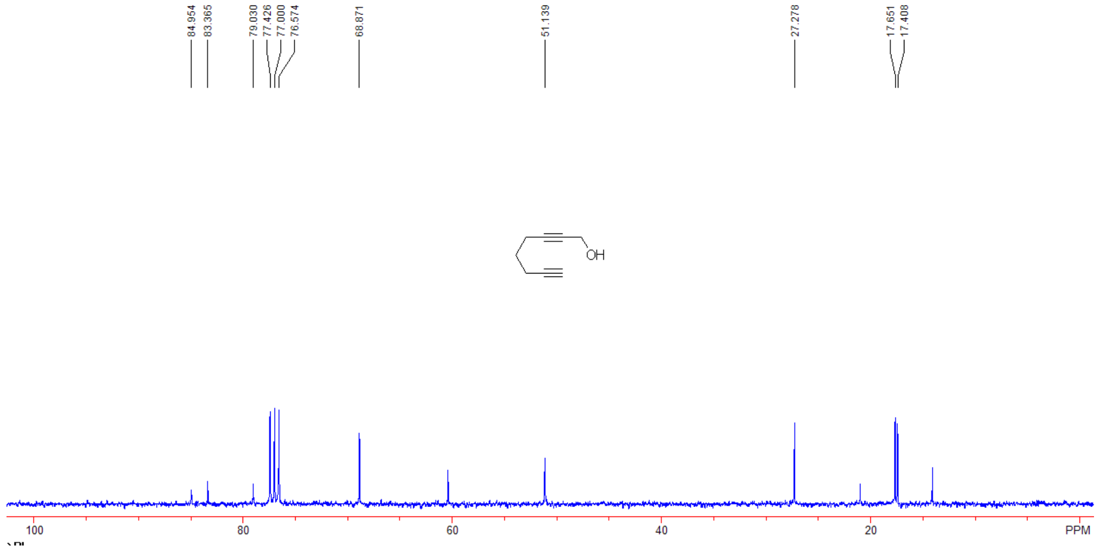

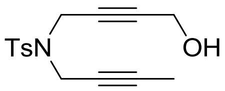

Compound SI-5e. 2.503 g, yield: 43%, light yellow oil. ^{1}H NMR ($CDCl_3$, 400 MHz, TMS) δ 1.65 (t, J = 2.4 Hz, 3H, CH_3), 2.05 (brs, 1H, OH), 2.43 (s, 3H, CH_3), 4.08 (q, J = 2.4 Hz, 2H, CH_2), 4.09 (t, J = 1.6 Hz, 2H, CH_2), 4.15 (t, J = 1.6 Hz, 2H, CH_2), 7.31 (d, J = 8.0 Hz, 2H, Ar), 7.72 (d, J = 8.0 Hz, 2H, Ar). ^{13}C NMR ($CDCl_3$, 100 MHz, TMS) δ 21.4, 36.4, 36.8, 50.7, 71.3, 78.3, 82.0, 83.8, 127.9, 129.3, 135.2, 143.7. IR (CH_2Cl_2) ν 3522, 2921, 1597, 1494, 1436, 1346, 1328, 1157, 1092, 900, 743 cm^{-1}. MS (ESI) m/e 309.1 (M^{+}+NH_4). HRMS (ESI) calcd. for $C_{15}H_{17}NO_3S$: 291.0929, Found: 291.0932.

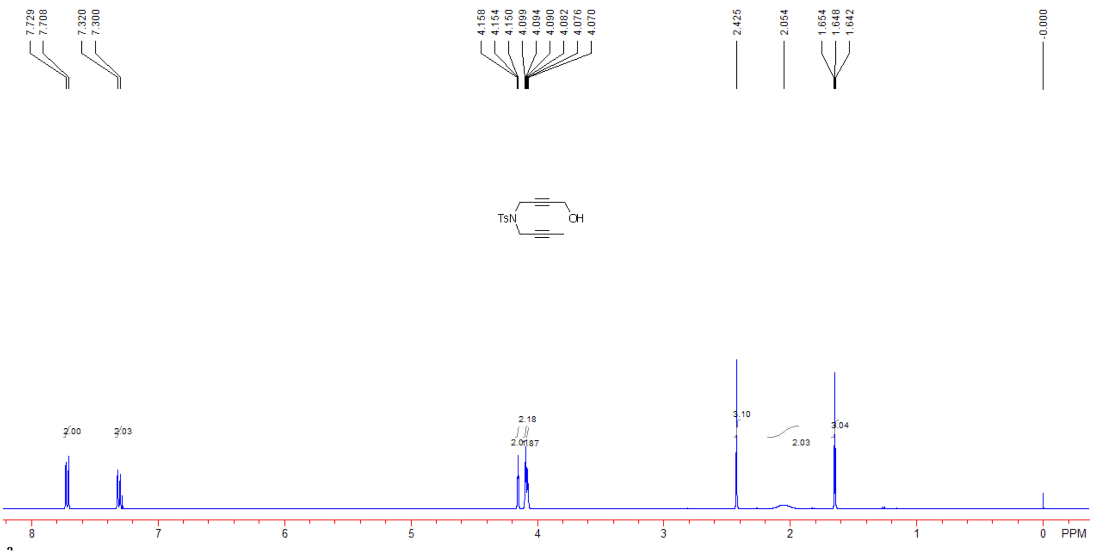

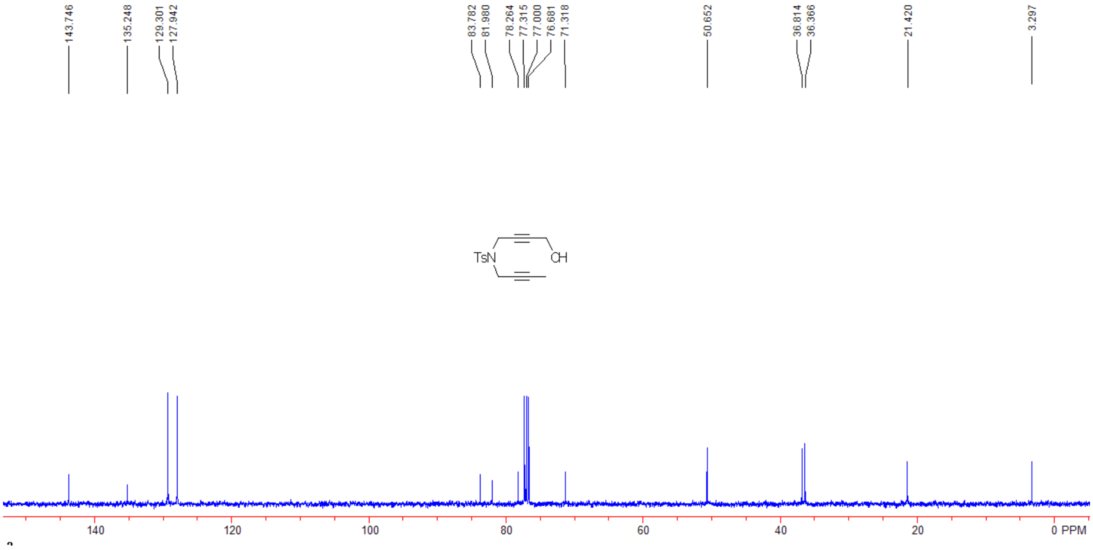

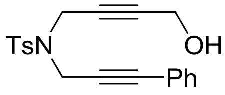

Compound SI-5f. 1.200 g, yield: 17%, light yellow oil. ^{1}H NMR ($CDCl_3$, 400 MHz, TMS) δ 1.76 (brs, 1H, OH), 2.36 (s, 3H, CH_3), 4.13 (t, *J* = 1.6 Hz, 2H, CH_2), 4.23 (s, 2H, CH_2), 4.39 (s, 2H, CH_2), 7.17 (d, *J* = 8.0 Hz, 2H, Ar), 7.25-7.30 (m, 5H, Ar), 7.76 (d, *J* = 8.0 Hz, 2H, Ar). ^{13}C NMR ($CDCl_3$, 100 MHz, TMS) δ 21.4, 36.8, 37.3, 50.8, 78.4, 81.4, 84.0, 85.8, 122.0, 128.0, 128.2, 128.5, 129.5, 131.6 135.3, 143.9. IR (CH_2Cl_2) ν 3522, 1597, 1490, 1442, 1348, 1328, 1159, 1093, 898, 757 cm^{-1}. MS (ESI) m/e 354.1 (M^++1). HRMS (ESI) calcd. for $C_{20}H_{19}NO_3S$: 353.1086, Found: 353.1082.

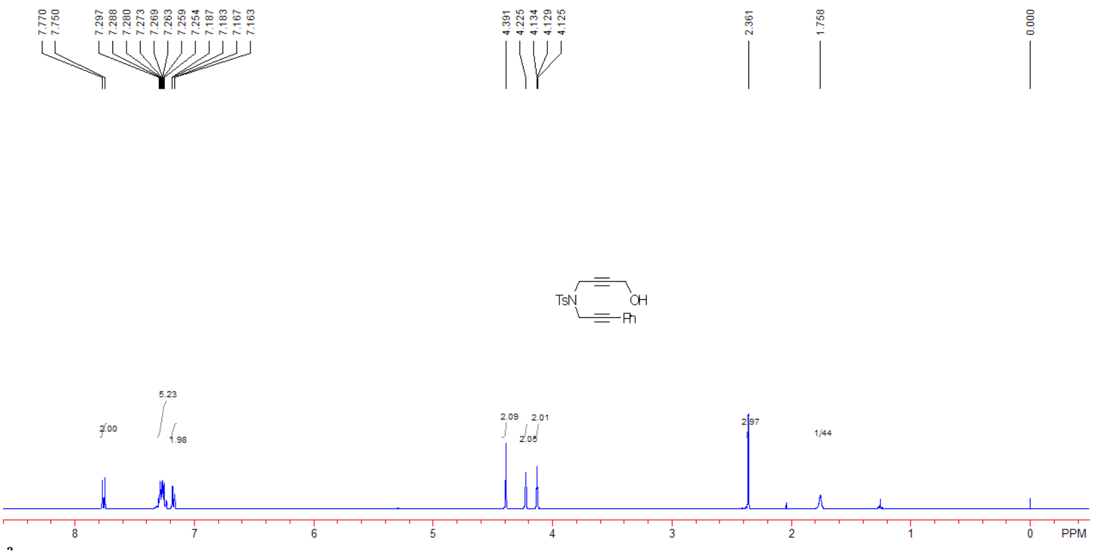

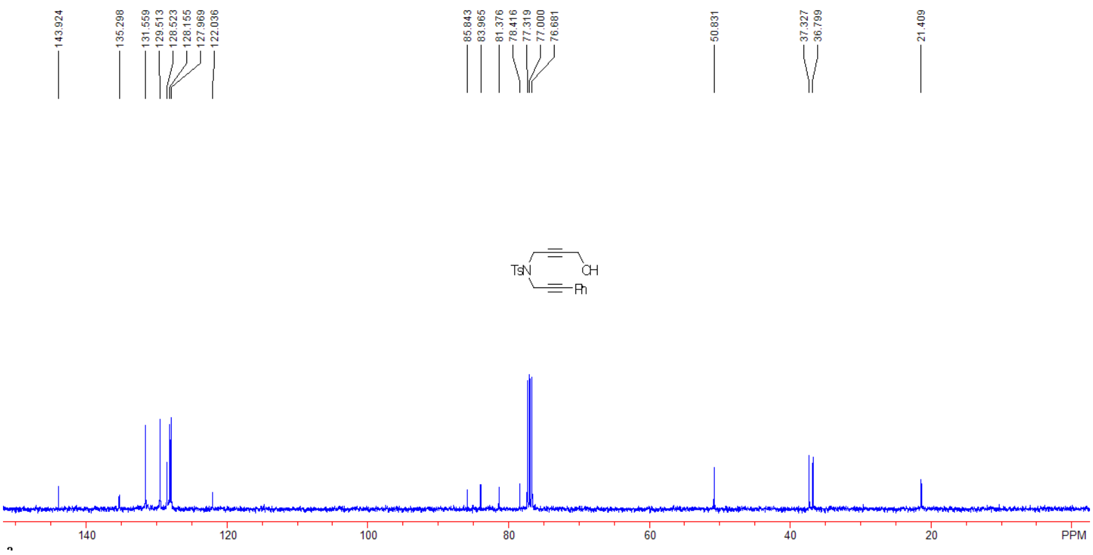

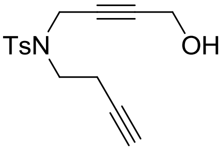

Compound SI-5g. 2.444 g, yield: 42%, light yellow oil. ^{1}H NMR ($CDCl_3$, 400 MHz, TMS) δ 1.81 (brs, 1H, OH), 2.02 (t, J = 2.4 Hz, 1H, ≡CH), 2.43 (s, 3H, CH_3), 2.51 (dt, J_1 = 7.2 Hz, J_2 = 2.4 Hz, 2H, CH_2), 3.37 (t, J = 7.2 Hz, 2H, CH_2), 4.02 (d, J = 4.2 Hz, 2H, CH_2), 4.21 (t, J = 1.6 Hz, 2H, CH_2), 7.32 (d, J = 8.4 Hz, 2H, Ar), 7.74 (d, J = 8.4 Hz, 2H, Ar). ^{13}C NMR ($CDCl_3$, 100 MHz, TMS) δ 18.9, 21.4, 37.6, 45.4, 50.6, 70.4, 78.5, 80.7, 83.9, 127.7, 129.4, 135.7, 143.8. IR (CH_2Cl_2) ν 3509, 3288, 2923, 1597, 1495, 1450, 1342, 1155, 1119, 1095, 657 cm^{-1}. MS (ESI) m/e 309.1 (M^++NH_4). HRMS (ESI) calcd. for $C_{15}H_{17}NO_3S$: 291.0929, Found:

291.0934.

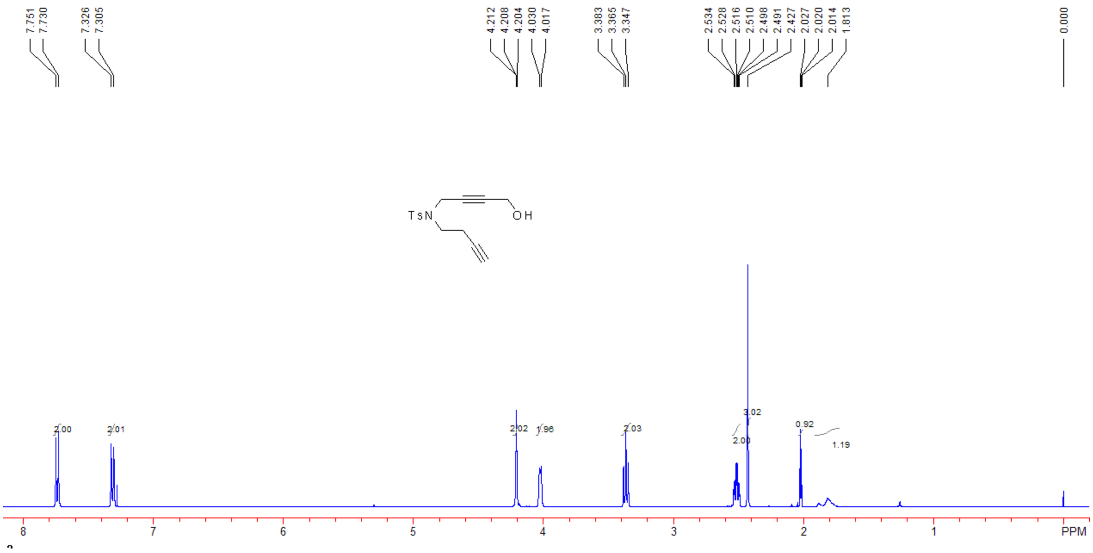

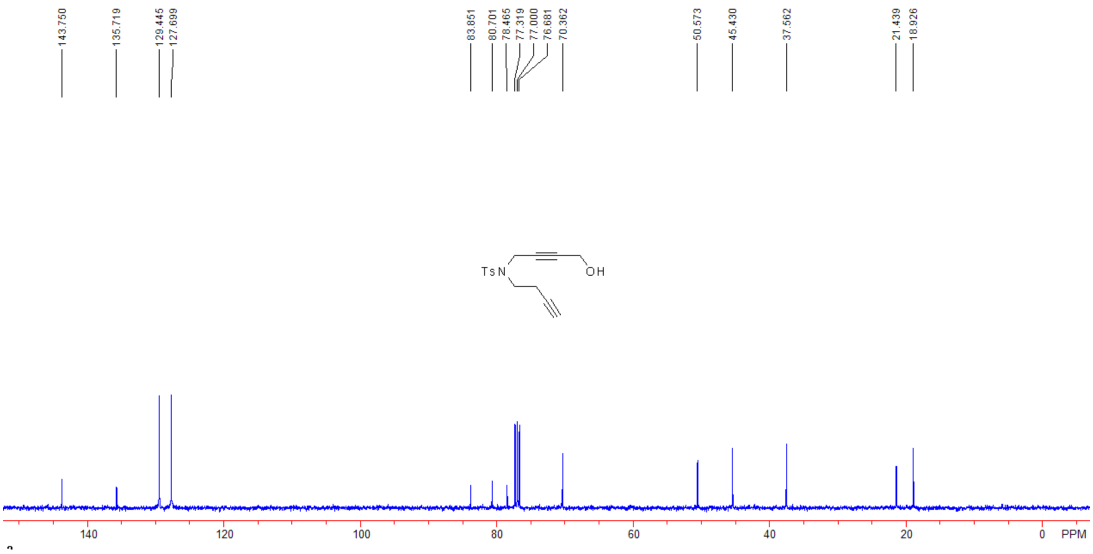

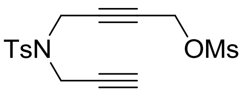

Compound SI-6a. 950 mg, yield: 75%, light yellow oil. ^{1}H NMR ($CDCl_3$, 400 MHz, TMS) δ 2.20 (s, 1H, ≡CH), 2.44 (s, 3H, CH_3), 3.06 (s, 3H, CH_3), 4.14 (s, 2H, CH_2), 4.23 (s, 2H, CH_2), 4.69 (s, 2H, CH_2), 7.33 (d, J = 8.0 Hz, 2H, Ar), 7.71 (d, J = 8.0 Hz, 2H, Ar). ^{13}C NMR ($CDCl_3$, 100 MHz, TMS) δ 21.4, 36.2, 36.4, 38.7, 57.1, 74.4, 75.9, 77.7, 82.3, 127.7, 129.6, 134.7, 144.2. IR (CH_2Cl_2) ν 3285, 1597, 1494, 1437, 1348, 1159, 1093, 942, 659 cm^{-1}. MS (ESI) m/e 373.1 (M^{+}+NH_4). HRMS (ESI) calcd. for $C_{15}H_{17}NO_5S_2$: 355.0548, Found: 355.0555.

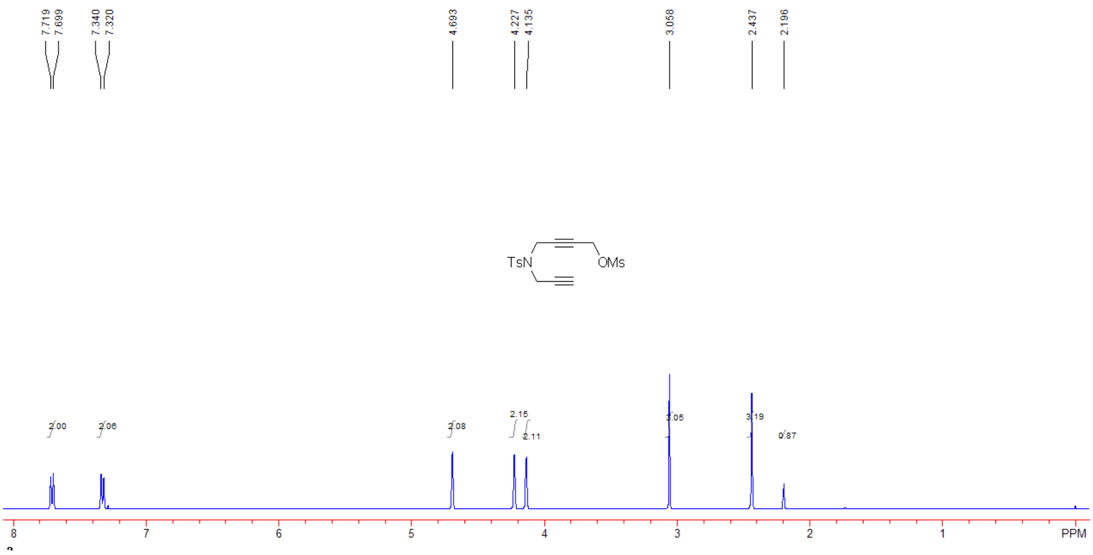

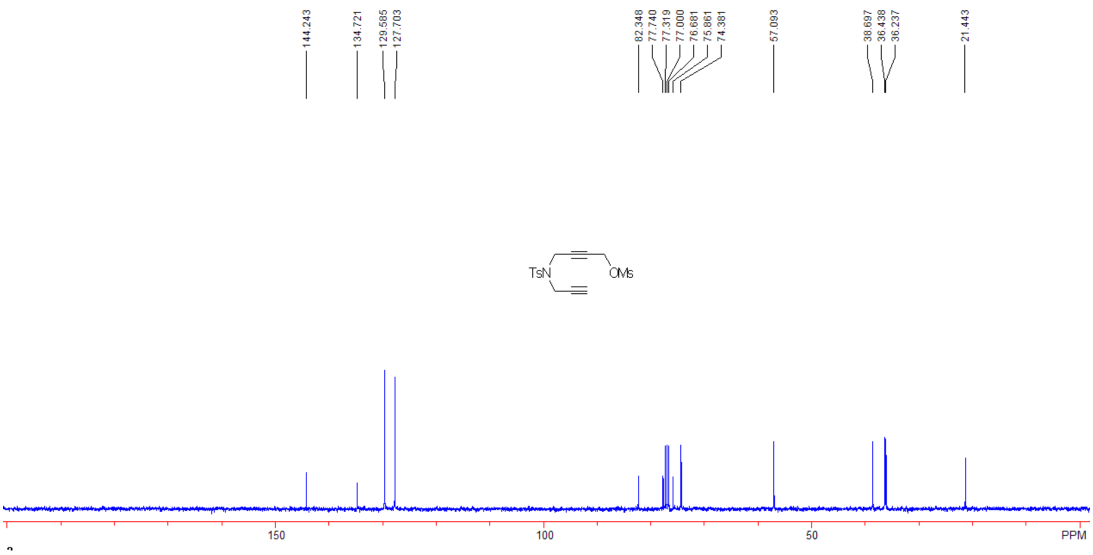

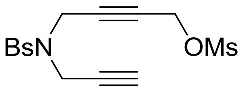

Compound SI-6b. 672 mg, yield: 80%, light yellow oil. ^{1}H NMR ($CDCl_3$, 400 MHz, TMS) δ 2.21 (t, J = 2.4 Hz, 1H, ≡CH), 3.08 (s, 3H, CH_3), 4.17 (d, J = 2.4 Hz, 2H, CH_2), 4.227-4.231 (m, 2H, CH_2), 4.73 (t, J = 1.6 Hz, 2H, CH_2), 7.67-7.72 (m, 4H, Ar). ^{13}C NMR ($CDCl_3$, 100 MHz, TMS) δ 36.4, 36.6, 38.8, 56.9, 74.7, 75.6, 78.1, 82.1, 128.3, 129.3, 132.3, 137.0. IR (CH_2Cl_2) ν 3284, 2934, 1574, 1472, 1390, 1349, 1162, 1092, 1069, 943, 761 cm^{-1}. MS (ESI) m/e 437.0 (M^{+}+NH_4). HRMS (ESI) calcd. for $C_{14}H_{14}BrNO_5S_2$: 418.9497, Found: 418.9499.

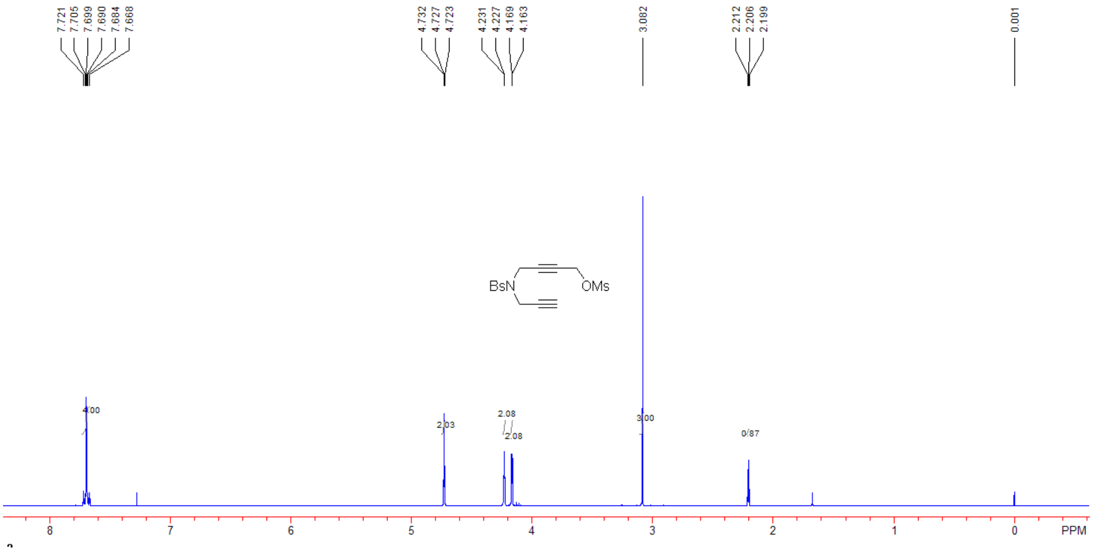

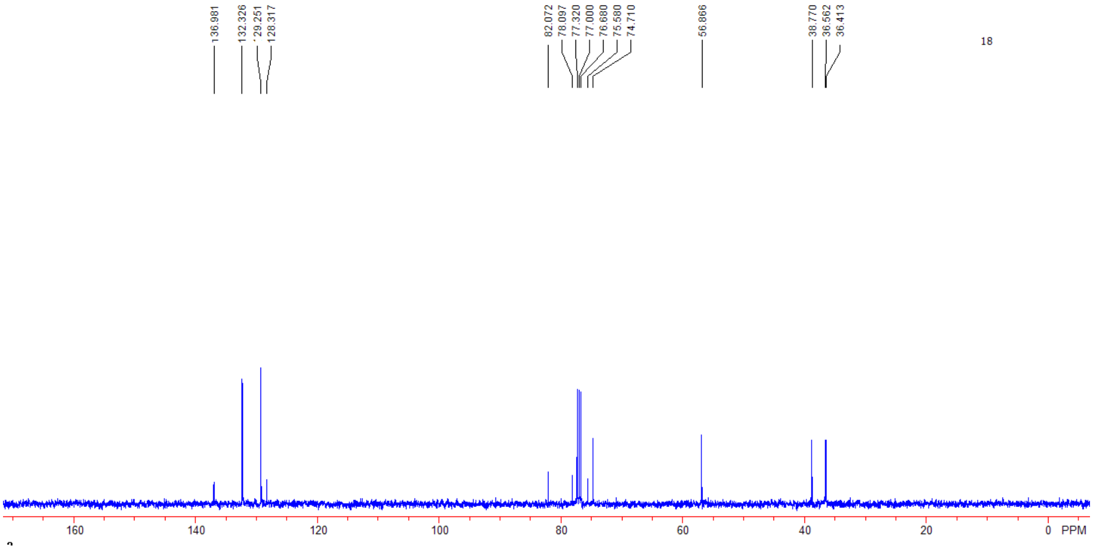

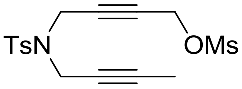

Compound SI-6e. 926 mg, yield: 84%, light yellow oil. ^{1}H NMR ($CDCl_3$, 400 MHz, TMS) δ 1.65 (d, J = 2.4 Hz, 3H, CH_3), 2.43 (s, 3H, CH_3), 3.06 (s, 3H, CH_3), 4.07 (d, J = 2.4 Hz, 2H, CH_2), 4.20 (s, 2H, CH_2), 4.70 (s, 2H, CH_2), 7.32 (d, J = 8.0 Hz, 2H, Ar), 7.71 (d, J = 8.0 Hz, 2H, Ar). ^{13}C NMR ($CDCl_3$, 100 MHz, TMS) δ 3.2, 21.4, 36.2, 37.0, 38.7, 57.2, 71.0, 77.4, 82.3, 82.8, 127.8, 129.4, 135.0, 144.0. IR (CH_2Cl_2) ν 3035, 2923, 1597, 1494, 1438, 1347, 1327, 1158, 1092, 942, 658 cm^{-1}. MS (ESI) m/e 387.1 (M^{+}+NH_4). HRMS (ESI) calcd. for $C_{16}H_{19}NO_5S_2$: 369.0705, Found: 369.0714.

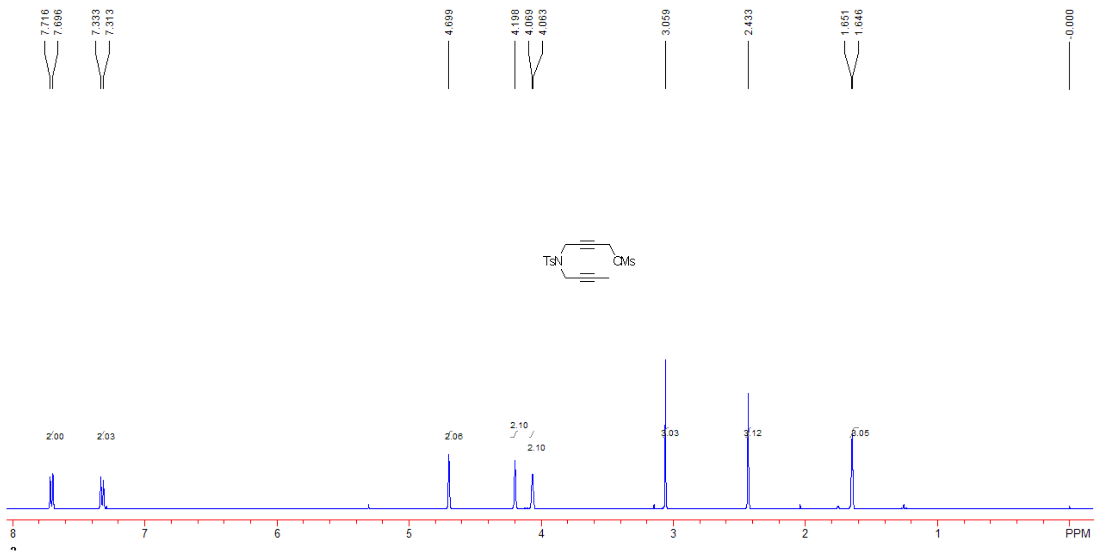

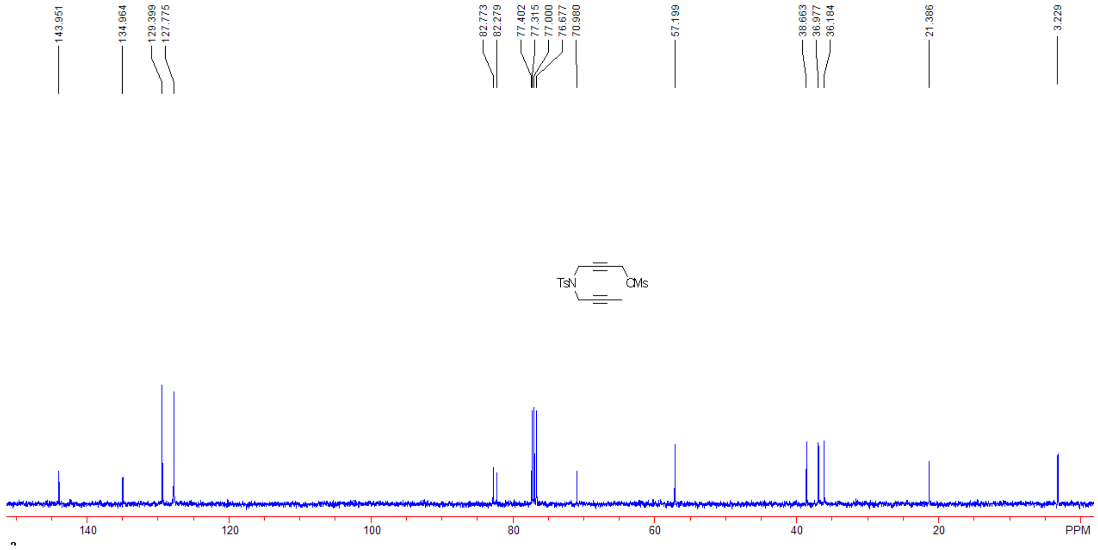

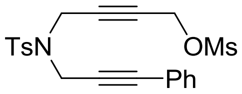

Compound SI-6f. 0.603 g, yield: 17%, light yellow oil. ^{1}H NMR ($CDCl_3$, 400 MHz, TMS) δ 2.37 (s, 3H, CH_3), 3.05 (s, 3H, CH_3), 4.27 (s, 2H, CH_2), 4.38 (s, 2H, CH_2), 4.73 (t, J = 2.0 Hz, 2H, CH_2), 7.15-7.17 (m, 2H, Ar), 7.26-7.31 (m, 5H, Ar), 7.75 (d, J = 8.4 Hz, 2H, Ar). ^{13}C NMR ($CDCl_3$, 100 MHz, TMS) δ 21.4, 36.6, 37.5, 38.8, 57.2, 77.7, 80.9, 82.8, 86.1, 121.8, 127.8, 128.2, 128.6, 129.6, 131.5, 134.9, 144.2. IR (CH_2Cl_2) ν 1597, 1490, 1442, 1348, 1159, 1092, 973, 943, 659 cm^{-1}. MS (ESI) m/e 449.1 (M^{+}+NH_4). HRMS (ESI) calcd. for $C_{21}H_{21}NO_5S_2$: 431.0861, Found: 431.0868.

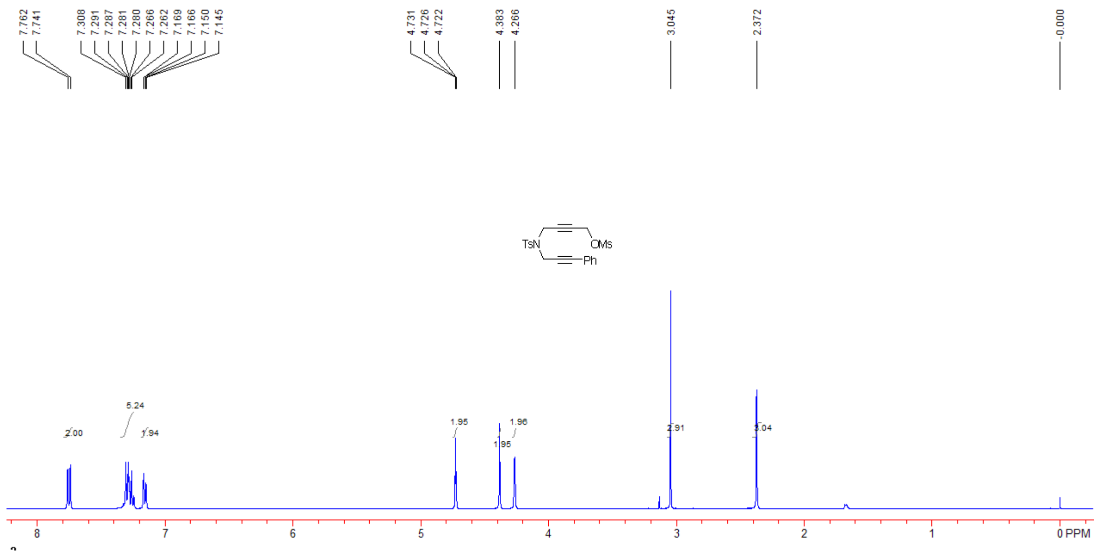

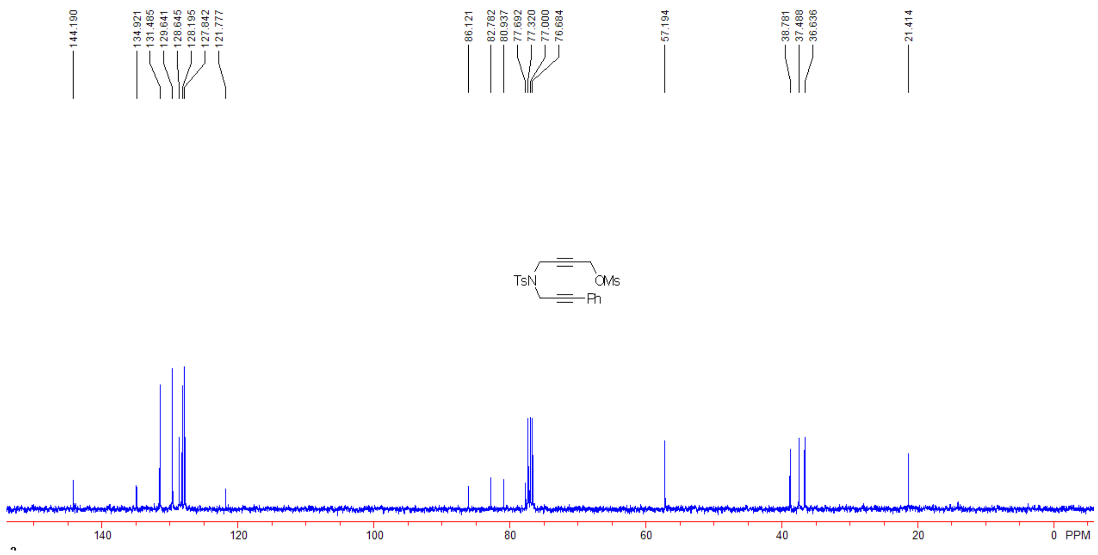

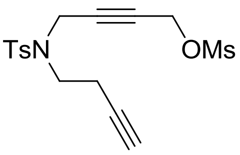

Compound SI-6g. 0.832 g, yield: 75%, light yellow oil. ^{1}H NMR ($CDCl_3$, 400 MHz, TMS) δ 2.03 (t, J = 2.4 Hz, 1H, ≡CH), 2.44 (s, 3H, CH_3), 2.51 (dt, J_1 = 7.2 Hz, J_2 = 2.4 Hz, 2H, CH_2), 3.02 (s, 3H, CH_3), 3.35 (t, J = 7.2 Hz, 2H, CH_2), 4.27 (t, J = 1.6 Hz, 2H, CH_2), 4.63 (t, J = 1.6 Hz, 2H, CH_2), 7.33 (d, J = 8.0 Hz, 2H, Ar), 7.73 (d, J = 8.0 Hz, 2H, Ar). ^{13}C NMR ($CDCl_3$, 100 MHz, TMS) δ 19.0, 21.4, 37.5, 38.6, 45.6, 56.9, 70.5, 77.5, 80.6, 82.9, 127.5, 129.6, 135.5, 144.0. IR (CH_2Cl_2) ν 3287, 2939, 1597, 1494, 1450, 1344, 1174, 1156, 1095, 938, 658 cm^{-1}. MS (ESI) m/e 387.1 (M^++NH_4). HRMS (ESI) calcd. for $C_{16}H_{19}NO_5S_2$: 369.0705, Found:

369.0711.

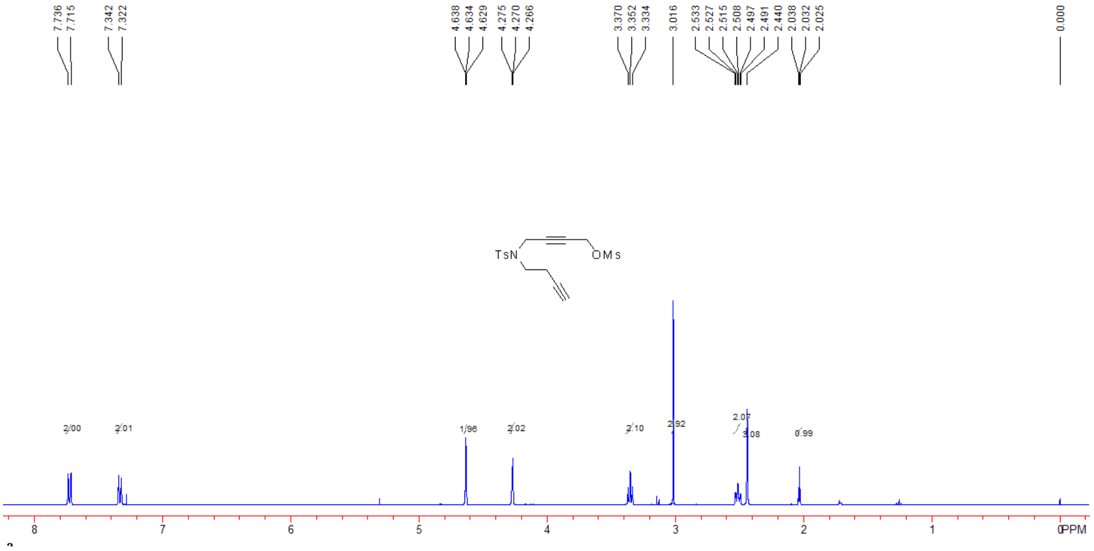

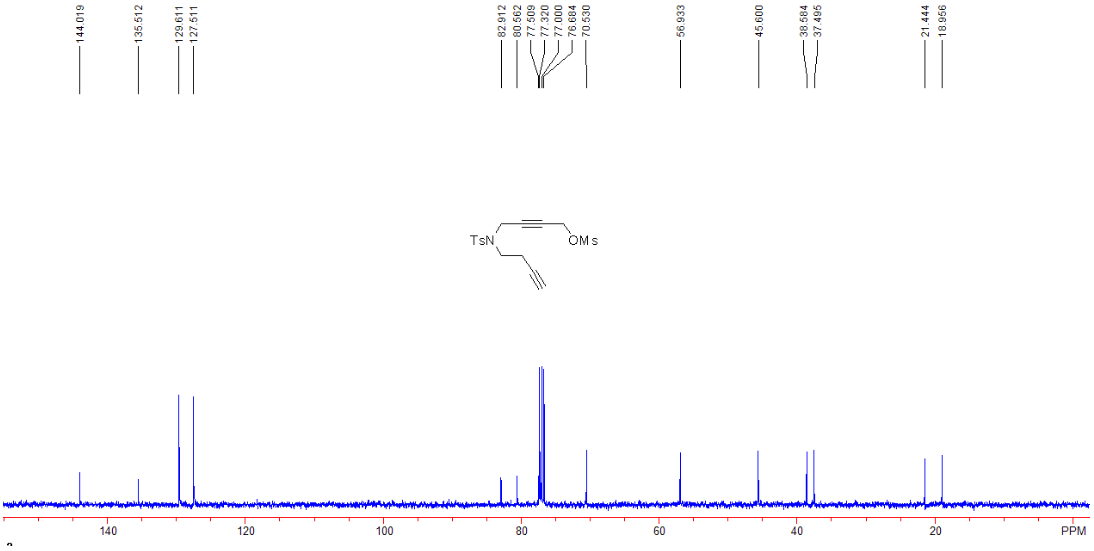

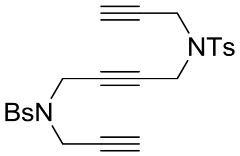

Compound 1d. 0.253 g, yield: 95%, white solid, Mp: 167-169 ^{o}C. ^{1}H NMR ($CDCl_3$, 400 MHz, TMS) δ 2.14-2.15 (m, 2H, ≡CH), 2.44 (s, 3H, CH_3), 4.015 (s, 3H, CH_3), 4.021 (s, 2H, CH_2), 4.026 (s, 2H, CH_2), 4.034 (s, 2H, CH_2), 7.32 (d, J = 8.0 Hz, 2H, Ar), 7.66 (s, 4H, Ar), 7.69 (d, J = 8.0 Hz, 2H, Ar). ^{13}C NMR ($CDCl_3$, 100 MHz, TMS) δ 21.5, 36.2, 36.27, 36.29, 36.4, 74.2, 74.4, 75.7, 76.0, 77.9, 78.7, 127.8, 128.2, 129.2, 129.6, 132.3, 135.0, 137.1, 144.1. IR (CH_2Cl_2) ν 3285, 2952, 2924, 2857, 1597, 1574, 1435, 1349, 1160, 1091, 1009, 660 cm^{-1}.

MS (ESI) m/e 533.0 (M^{+}+1). HRMS (ESI) calcd. for $C_{23}H_{21}BrN_2NaO_4S_2$: 555.0023, Found: 555.0018.

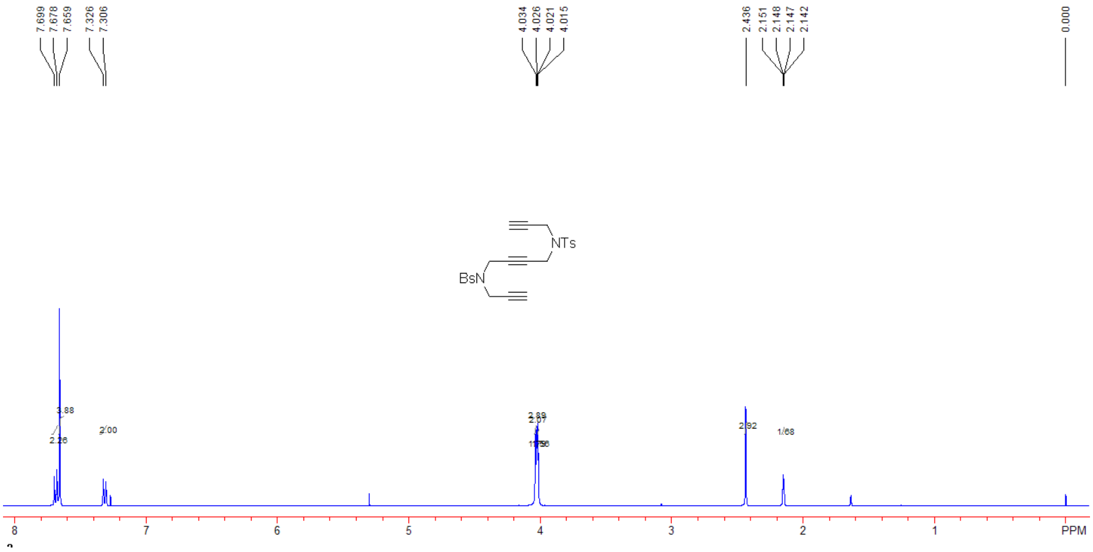

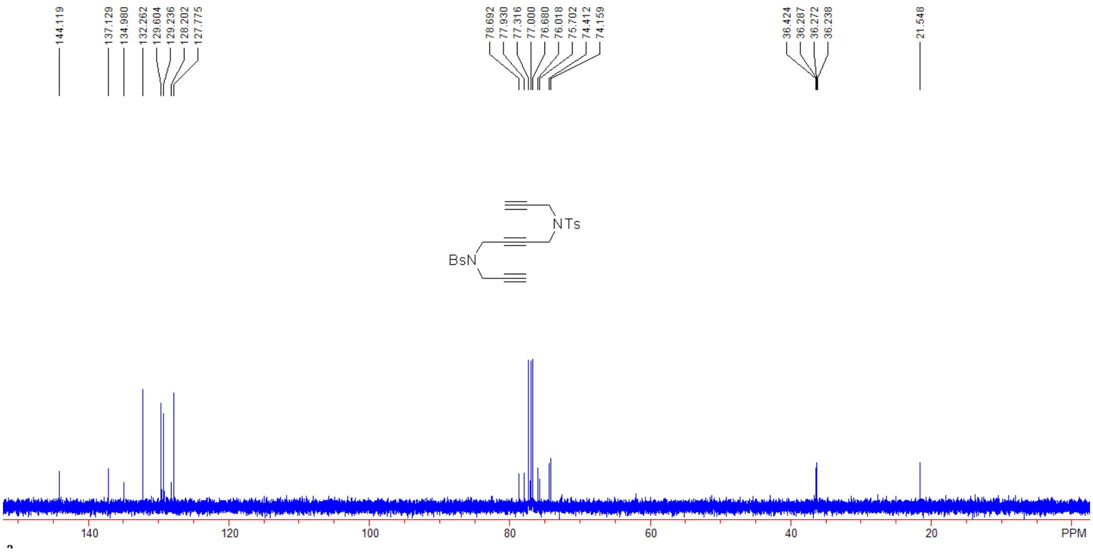

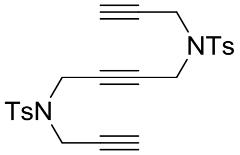

Compound 1e. 0.258 g, yield: 55%, white solid, Mp: 167-169 °C. ^{1}H NMR ($CDCl_3$, 400 MHz, TMS) δ 2.13 (s, 1H, ≡CH), 2.44 (s, 3H, CH_3), 3.99 (s, 2H, CH_2), 4.02 (s, 2H, CH_2), 7.31 (d, *J* = 8.0 Hz, 2H, Ar), 7.67 (d, *J* = 8.0 Hz, 2H, Ar). ^{13}C NMR ($CDCl_3$, 100 MHz, TMS) δ 21.5, 36.2, 36.3, 74.0, 76.1, 78.3, 127.8, 129.6, 135.0, 144.1. IR (CH_2Cl_2) ν 3264, 2925, 1596, 1493, 1438, 1339, 1322, 1156, 1091, 896, 747 cm^{-1}. MS (ESI) m/e 486.2 (M^{+}+NH_4). HRMS (ESI)

calcd. for $C_{24}H_{24}N_2O_4S_2$: 468.1177, Found: 468.1184.

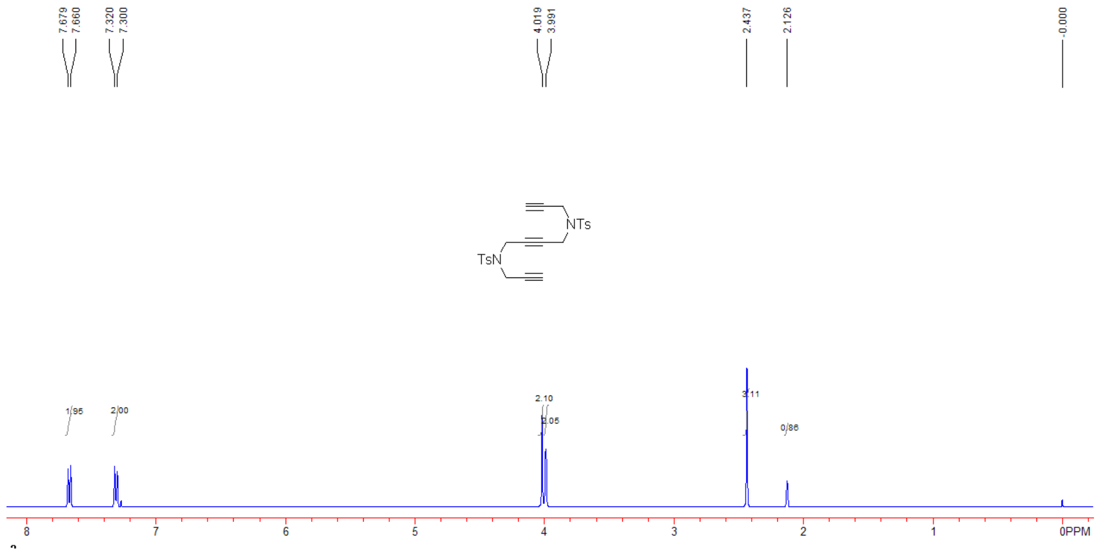

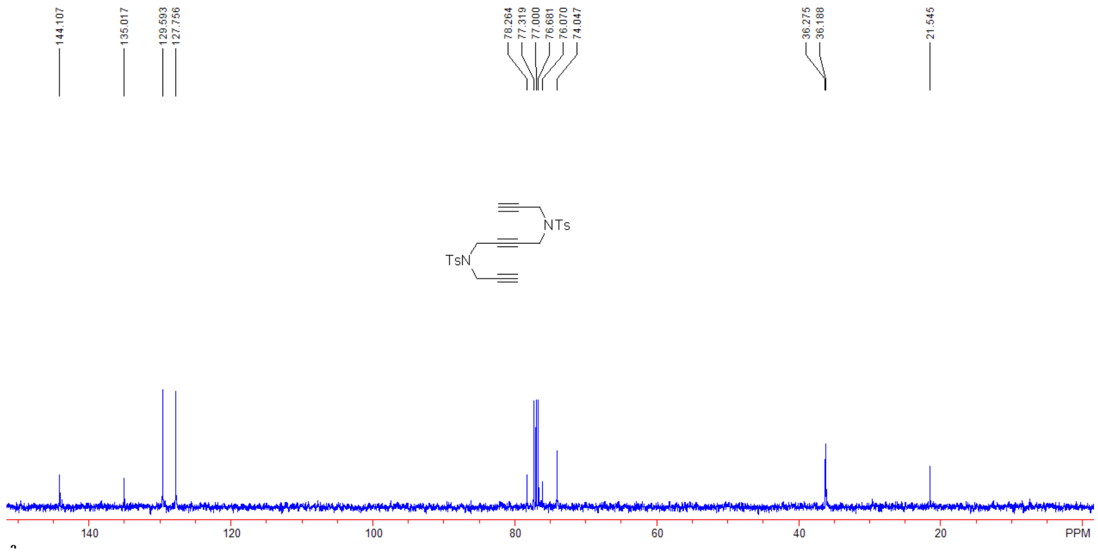

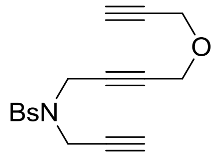

Compound 1f. 0.418 g, yield: 55%, light yellow oil. ^{1}H NMR ($CDCl_3$, 400 MHz, TMS) δ 2.19 (t, J = 2.4 Hz, 1H, ≡CH), 2.47 (t, J = 2.4 Hz, 1H, ≡CH), 4.12 (s, 2H, CH_2), 4.13 (d, J = 2.4 Hz, 2H, CH_2), 4.16 (d, J = 2.4 Hz, 2H, CH_2), 4.22 (s, 2H, CH_2), 7.67 (d, J = 8.8 Hz, 2H, Ar), 7.71 (d, J = 8.8 Hz, 2H, Ar). ^{13}C NMR ($CDCl_3$, 100 MHz, TMS) δ 36.5, 36.6, 56.4, 56.5, 74.4, 75.3, 75.9, 78.5, 79.0, 81.3, 128.2, 129.3, 132.2, 137.2. IR (CH_2Cl_2) ν 3289, 1574, 1472, 1390, 1352, 1164, 1069, 1010, 762 cm^{-1}. MS (ESI) m/e 397.0 (M^++NH_4). HRMS (ESI) calcd.

for $C_{16}H_{14}BrNO_3S$: 378.9878, Found: 378.9886.

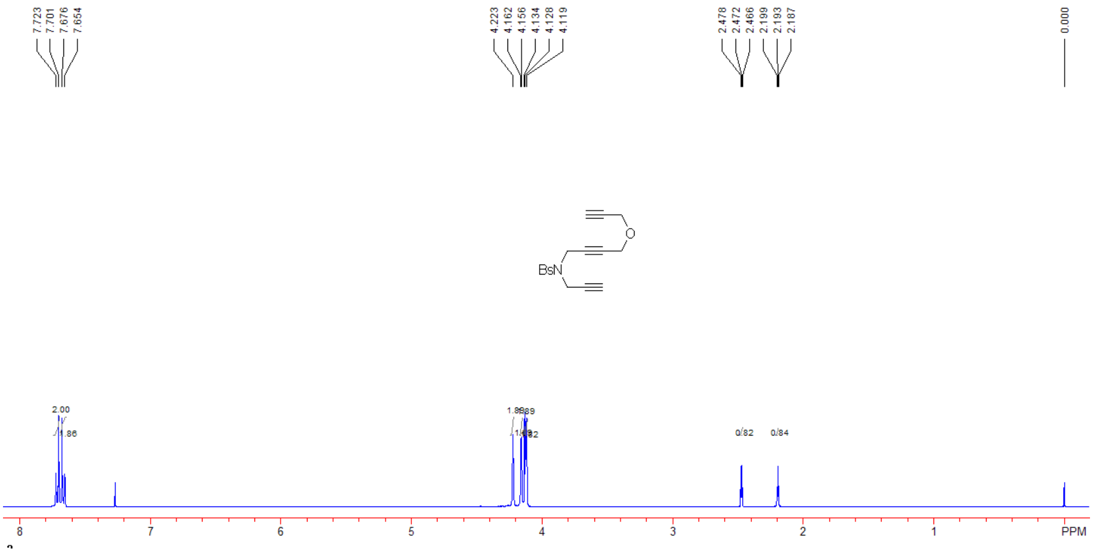

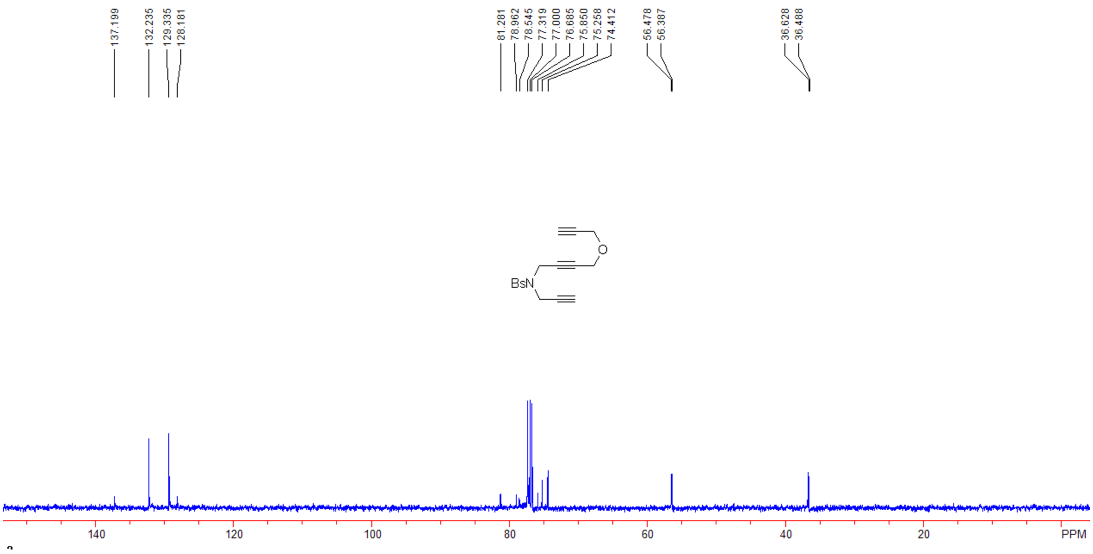

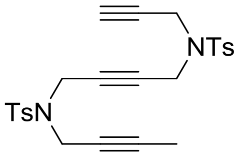

Compound 1j. 0.458 g, yield: 95%, white solid, Mp: 126-128 °C. ^{1}H NMR ($CDCl_3$, 400 MHz, TMS) δ 1.63 (t, J = 2.4 Hz, 3H, CH_3), 2.13 (t, J = 2.0 Hz, 1H, ≡CH), 2.43 (s, 3H, CH_3), 2.44 (s, 3H, CH_3), 3.92 (q, J = 2.4 Hz, 2H, CH_2), 3.99-4.00 (m, 4H, CH_2), 4.02 (s, 2H, CH_2), 7.30 (d, J = 8.0 Hz, 2H, Ar), 7.31 (d, J = 8.0 Hz, 2H, Ar), 7.66 (d, J = 8.0 Hz, 2H, Ar), 7.67 (d, J = 8.0 Hz, 2H, Ar). ^{13}C NMR ($CDCl_3$, 100 MHz, TMS) δ 3.3, 21.47, 21.49, 36.1, 36.2, 36.3, 36.7, 71.1, 74.0, 76.0, 77.8, 78.6, 81.9, 127.7, 127.8, 129.4, 129.5, 134.9, 135.1, 143.8, 144.1. IR

(CH_2Cl_2) ν 3279, 2922, 1597, 1494, 1435, 1348, 1330, 1159, 1092, 897, 659 cm^{-1}. MS (ESI) m/e 500.2 (M^++NH_4). HRMS (ESI) calcd. for $C_{25}H_{26}N_2O_4S_2$: 482.1334, Found: 482.1335.

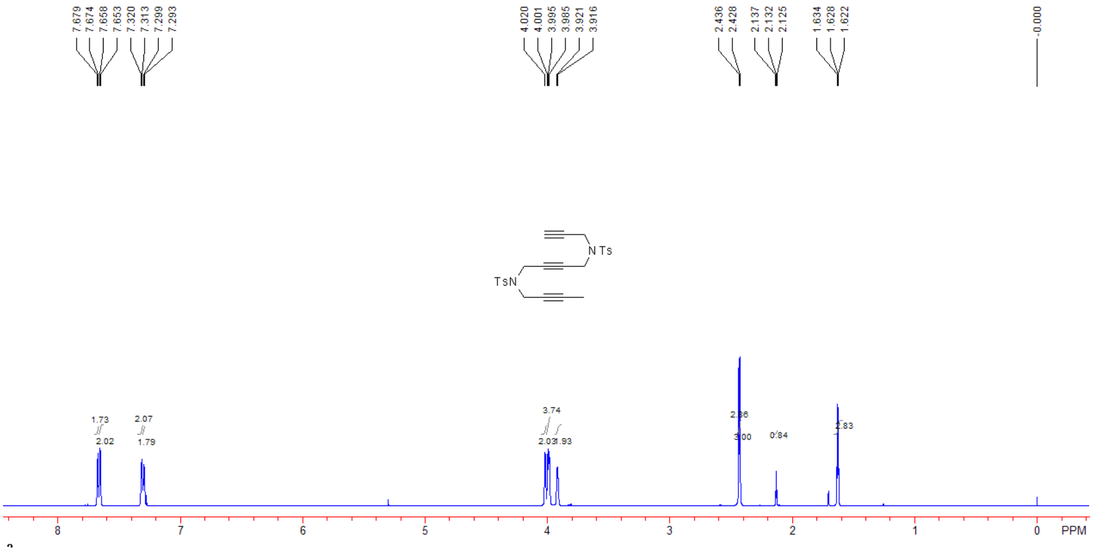

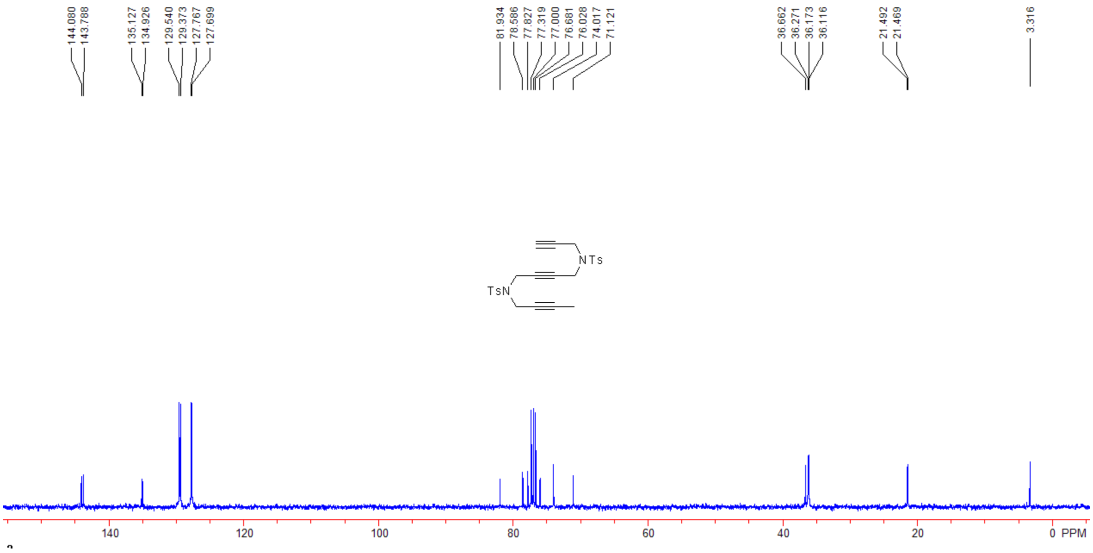

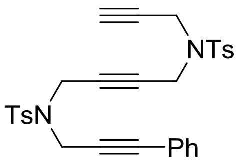

Compound 1k. 0.245 g, yield: 90%, white solid, Mp: 110-112 °C. ^{1}H NMR ($CDCl_3$, 400 MHz, TMS) δ 2.13 (t, J = 2.4 Hz, 3H, CH_3), 2.37 (s, 3H, CH_3), 2.41 (s, 3H, CH_3), 4.03 (d, J = 2.4 Hz, 2H, CH_2), 4.04 (s, 2H, CH_2), 4.07 (s, 2H, CH_2), 4.22 (s, 2H, CH_2), 7.13-7.16 (m, 2H, Ar), 7.24-7.31 (m, 7H, Ar), 7.67 (d, J = 8.0 Hz, 2H, Ar), 7.71 (d, J = 8.0 Hz, 2H, Ar). ^{13}C NMR ($CDCl_3$, 100 MHz, TMS) δ 21.4, 21.5, 36.3, 36.4, 36.5, 37.1, 74.1, 76.1, 78.1, 78.6, 81.1, 85.9,

121.9, 127.76, 127.80, 128.2, 128.6, 129.59, 129.61, 131.5, 134.97, 135.03, 144.0, 144.1. IR (CH_2Cl_2) ν 3286, 1597, 1491, 1442, 1349, 1330, 1161, 1092, 896, 660 cm^{-1}. MS (ESI) m/e 562.2 (M^++NH_4). HRMS (ESI) calcd. for $C_{30}H_{28}N_2O_4S_2$: 544.1490, Found: 544.1480.

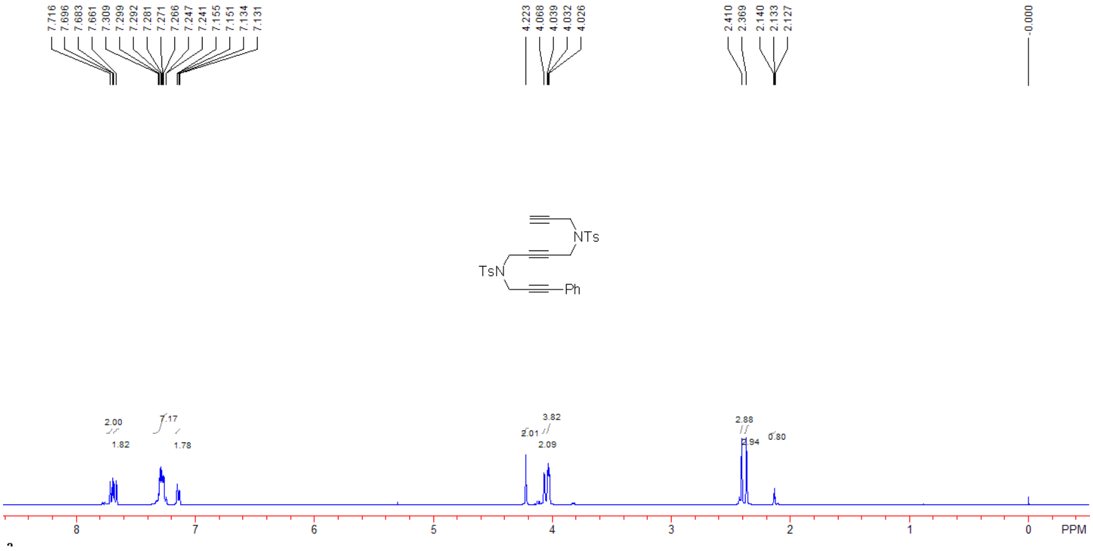

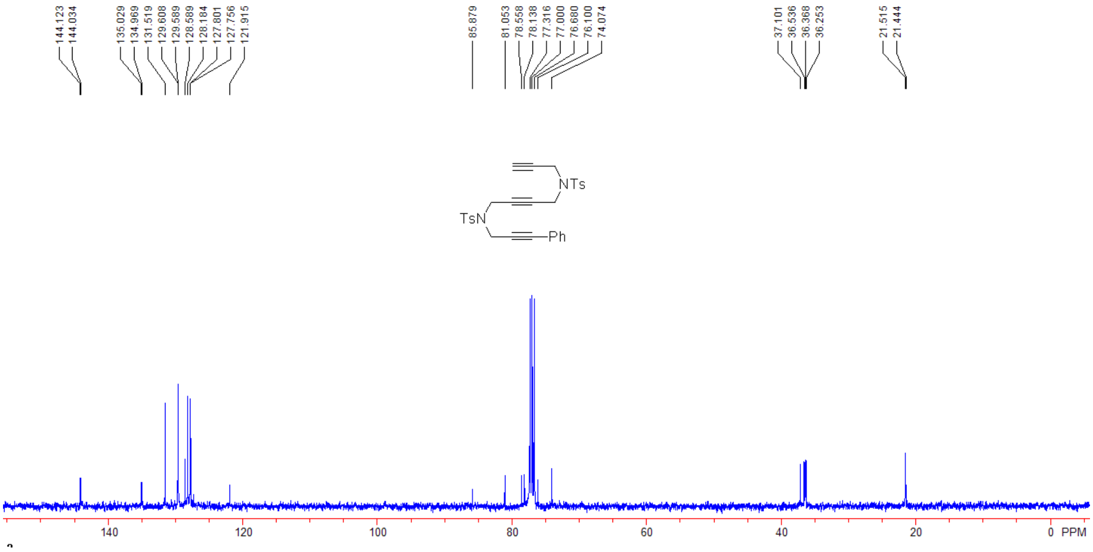

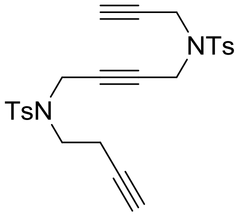

Compound 1l. 0.389 g, yield: 81%, white solid, Mp: 114-116 °C. ^{1}H NMR ($CDCl_3$, 400 MHz, TMS) δ 2.02 (t, J = 2.4 Hz, 1H, ≡CH), 2.13 (t, J = 2.4 Hz, 1H, ≡CH), 2.41-2.43 (m, 8H, CH_2 and CH_3), 3.24 (t, J = 3.2 Hz, 2H, CH_3), 3.90 (d, J = 2.4 Hz, 2H, CH_2), 3.94 (s, 2H, CH_2), 4.07

(s, 2H, CH_2), 7.30 (d, J = 8.0 Hz, 2H, Ar), 7.32 (d, J = 8.0 Hz, 2H, Ar), 7.65 (d, J = 8.0 Hz, 2H, Ar), 7.68 (d, J = 8.0 Hz, 2H, Ar). ^{13}C NMR ($CDCl_3$, 100 MHz, TMS) δ 18.8, 21.5, 36.0, 36.1, 37.3, 45.3, 70.4, 74.1, 75.9, 78.0, 78.6, 80.6, 127.3, 127.4, 127.7, 129.5, 129.6, 134.9, 135.5, 143.8, 144.0. IR (CH_2Cl_2) ν 3285, 2924, 1597, 1494, 1432, 1329, 1156, 1092, 896, 657 cm^{-1}. MS (ESI) m/e 500.2 (M^++NH_4). HRMS (ESI) calcd. for $C_{25}H_{26}N_2O_4S_2$: 482.1334, Found: 482.1337.

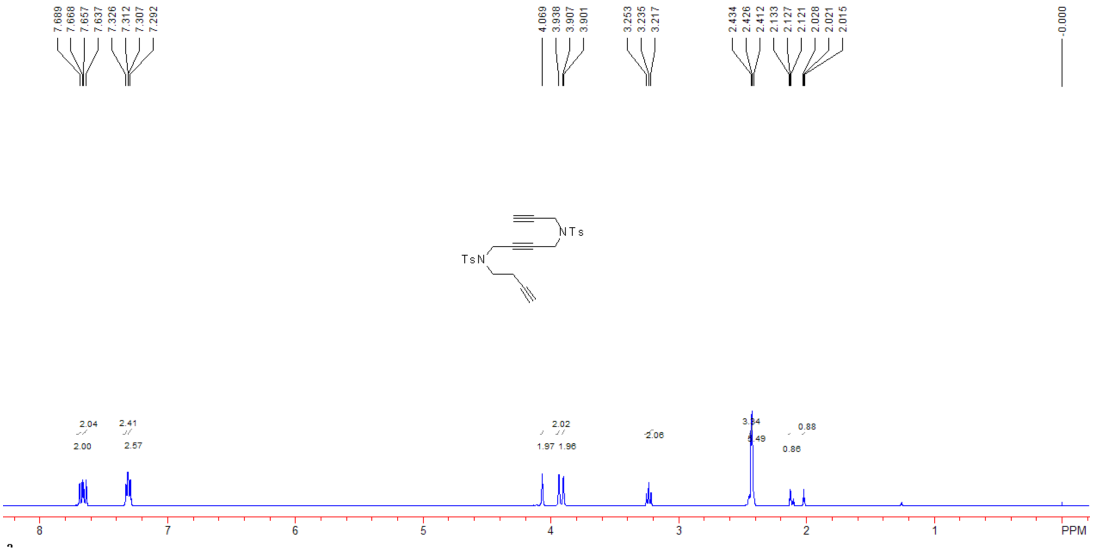

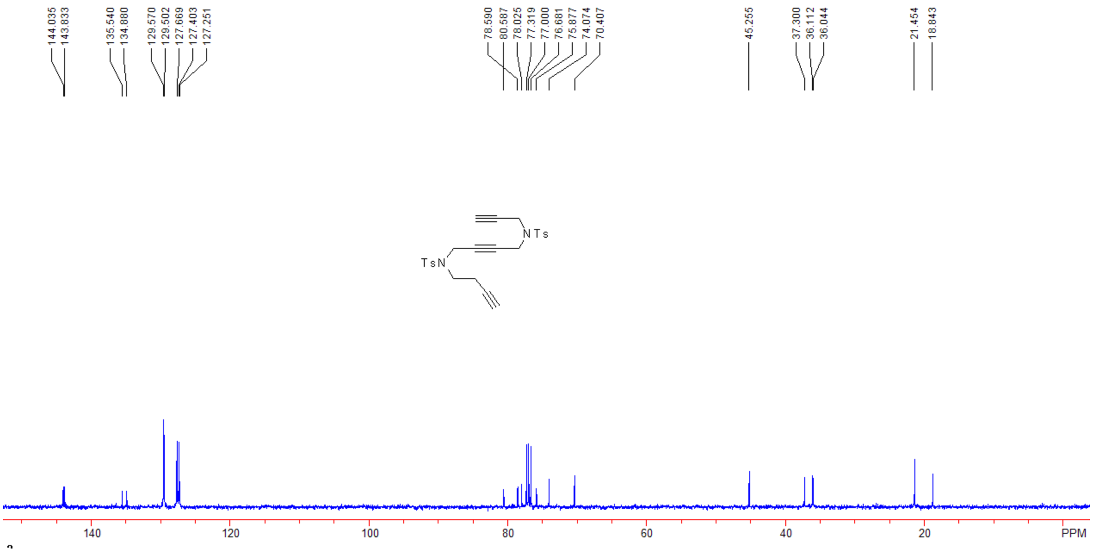

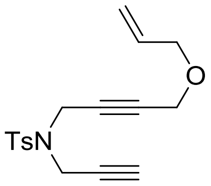

Compound 3c. 0.256 g, yield: 80%, colorless oil. ^{1}H NMR ($CDCl_3$, 400 MHz, TMS) δ 2.18 (t,

J = 1.2 Hz, 1H, ≡CH), 2.42 (s, 3H, CH_3), 3.92 (dd, J_1 = 5.6 Hz, J_2 = 1.2 Hz, 1H, CH_2), 3.99 (d, J = 1.2 Hz, 1H, CH_2), 4.09-4.23 (m, 6H, CH_2), 5.19-5.29 (m, 2H, =CH_2), 5.80-5.89 (m, 1H, =CH), 7.30 (d, J = 8.0 Hz, 2H, Ar), 7.71 (d, J = 8.0 Hz, 2H, Ar). ^{13}C NMR ($CDCl_3$, 100 MHz, TMS) δ 21.4, 36.2, 36.4, 56.9, 70.4, 74.0, 76.1, 78.5, 81.7, 117.9, 127.7, 129.4, 133.6, 134.9, 143.9. IR (CH_2Cl_2) ν 2957, 2926, 2857, 1736, 1652, 1457, 1374, 697, 637 cm^{-1}. MS (ESI) m/e 318.1 (M^++1). HRMS (ESI) calcd. for $C_{17}H_{19}NO_3S$: 317.1086, Found: 317.1084.

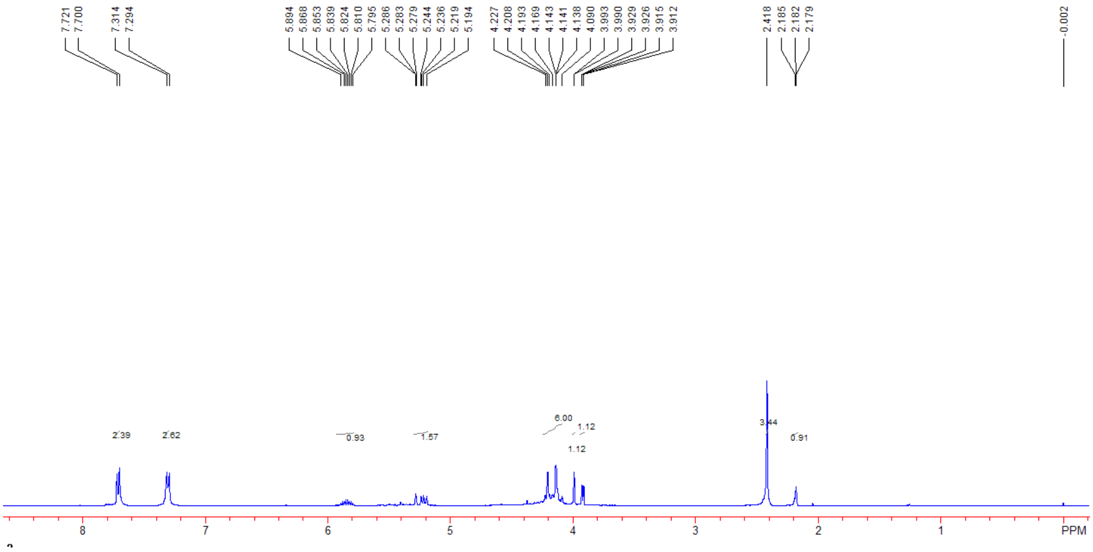

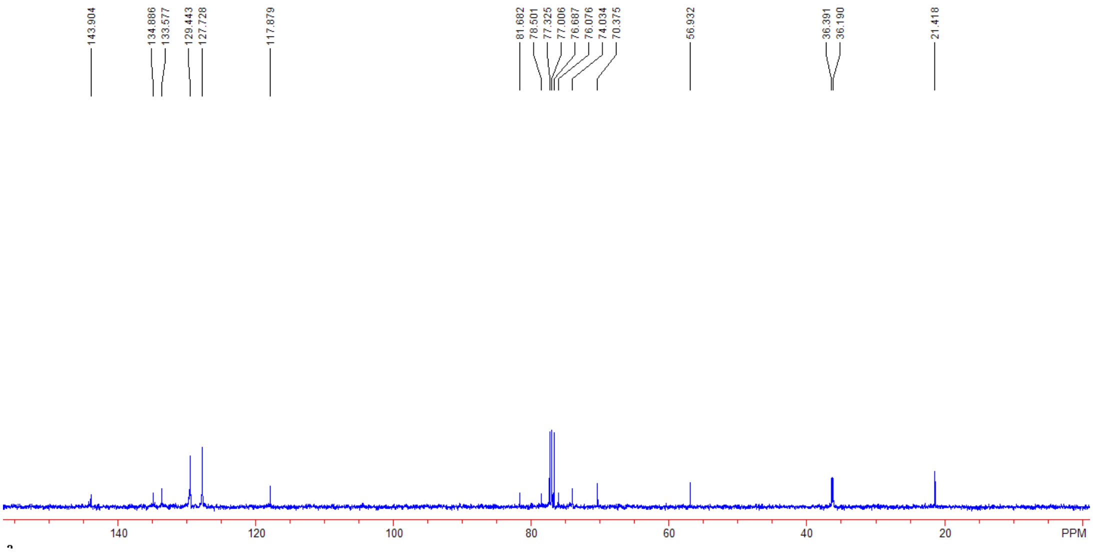

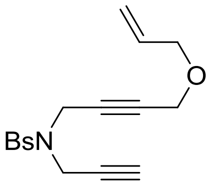

Compound 3d. 0.382 g, yield: 50%, colorless oil. ^1H NMR ($CDCl_3$, 400 MHz, TMS) δ 2.20 (t,

J = 2.4 Hz, 1H, ≡CH), 3.94 (d, J = 5.6 Hz, 2H, CH_2), 4.00 (s, 2H, CH_2), 4.16 (d, J = 2.4 Hz, 2H, CH_2), 4.22 (s, 2H, CH_2), 5.22 (d, J = 10.4 Hz, 1H, =CH_2), 5.28 (d, J = 17.2 Hz, 1H, =CH_2), 5.82-5.91 (m, 1H, =CH), 7.66 (d, J = 8.4 Hz, 2H, Ar), 7.71 (d, J = 8.4 Hz, 2H, Ar). ^{13}C NMR ($CDCl_3$, 100 MHz, TMS) δ 36.4, 36.6, 57.0, 70.6, 74.4, 75.8, 78.2, 82.1, 118.0, 128.1, 129.3, 132.2, 133.6, 137.1. IR (CH_2Cl_2) ν 3286, 3095, 1574, 1472, 1390, 1351, 1163, 1091, 1068, 1009, 896, 761 cm^{-1}. MS (ESI) m/e 399.0 (M^++NH_4). HRMS (ESI) calcd. for $C_{16}H_{16}BrNO_3S$: 381.0034, Found: 381.0034.

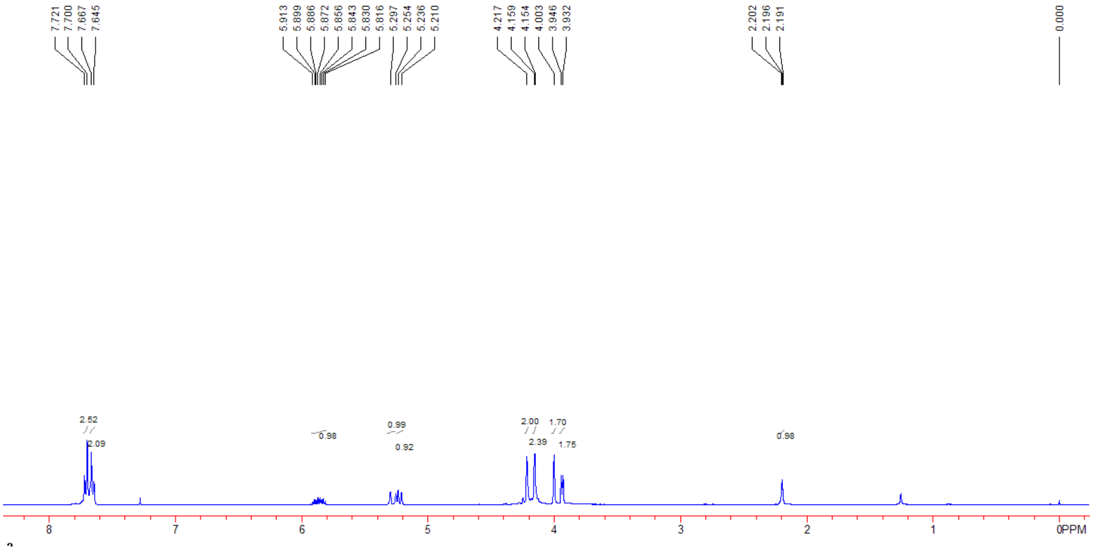

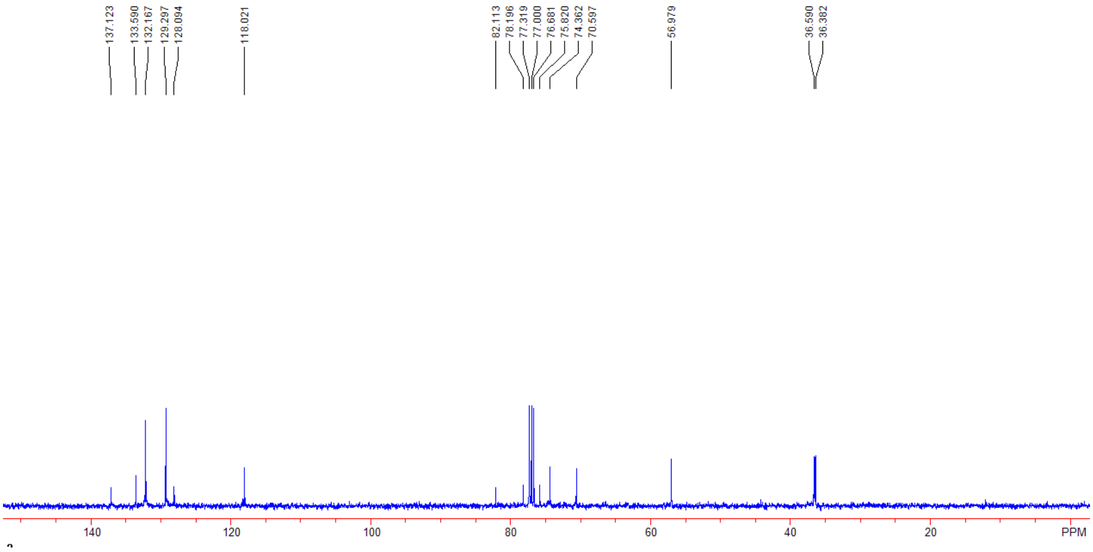

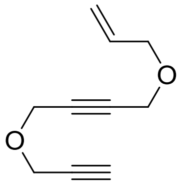

Compound 3e. 1.394 g, yield: 85%, colorless oil. ^{1}H NMR ($CDCl_3$, 400 MHz, TMS) δ 2.46 (t, J = 2.4 Hz, 1H, ≡CH), 4.07 (dt, J_1 = 6.0 Hz, J_2 = 1.6 Hz, 2H, CH_2), 4.21 (t, J = 1.6 Hz, 2H, CH_2), 4.26 (d, J = 2.4 Hz, 2H, CH_2), 4.32 (t, J = 1.6 Hz, 2H, CH_2), 5.22-5.25 (m, 1H, =CH_2), 5.29-5.34 (m, 1H, =CH_2), 5.86-5.94 (m, 1H, =CH). ^{13}C NMR ($CDCl_3$, 100 MHz, TMS) δ 56.4, 56.7, 57.3, 70.6, 75.0, 78.8, 81.3, 82.9, 118.0, 133.8. IR (CH_2Cl_2) ν 3287, 2912, 2858, 1443, 1345, 1245, 1120, 1070, 933 cm^{-1}. MS (ESI) m/e 165.0 (M^++1). HRMS (ESI) calcd. for $C_{10}H_{12}NaO_2$: 187.0730, Found: 187.0729.

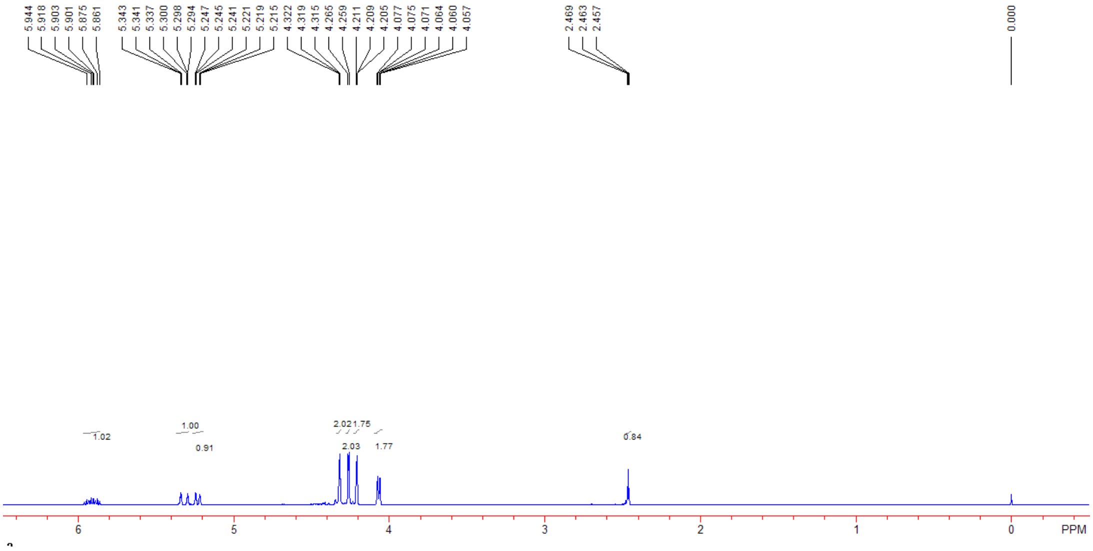

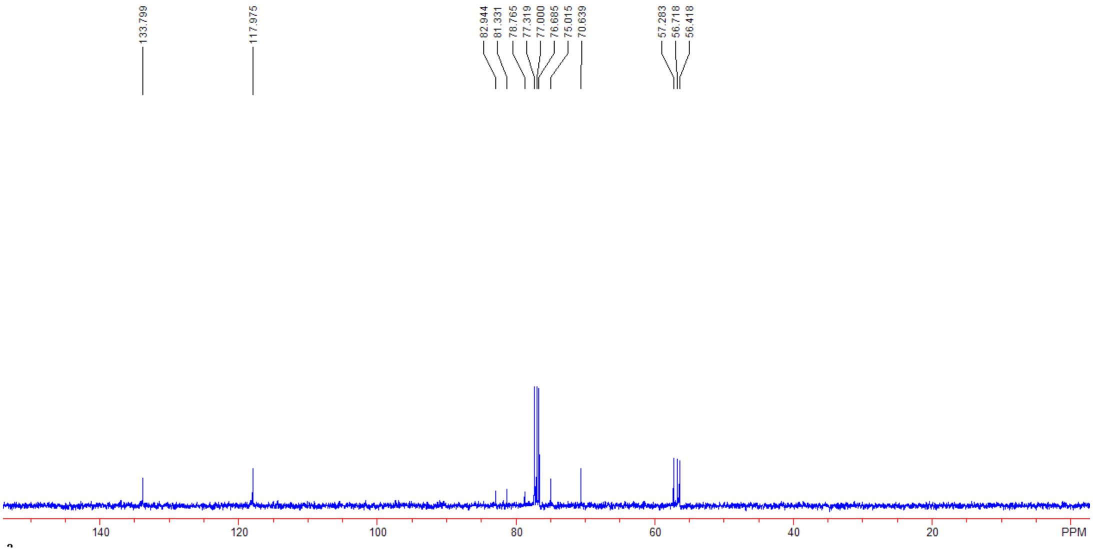

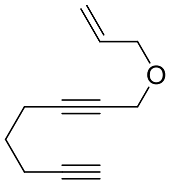

Compound 3f. 1.222 g, yield: 82%, colorless oil. ^{1}H NMR ($CDCl_3$, 400 MHz, TMS) δ 1.71-1.78 (m, 2H, CH_2), 1.97 (t, J = 2.4 Hz, 1H, ≡CH), 2.30-2.39 (m, 4H, CH_2), 4.05 (dt, J_1 = 6.0 Hz, J_2 = 1.6 Hz, 2H, CH_2), 4.14 (t, J = 2.4 Hz, 2H, CH_2), 5.20-5.23 (m, 1H, =CH_2), 5.28-5.33 (m, 1H, =CH_2), 5.87-5.96 (m, 1H, =CH). ^{13}C NMR ($CDCl_3$, 100 MHz, TMS) δ 17.5, 17.8, 27.4, 57.6, 68.8, 70.4, 76.6, 83.4, 85.6, 117.7, 134.1. IR (CH_2Cl_2) ν 3297, 2938, 2908, 2852, 1456, 1432, 1353, 1132, 1078, 925 cm^{-1}. MS (ESI) m/e 180.1 (M^{+}+NH_4). HRMS (ESI) calcd. for $C_{11}H_{14}NaO$: 185.0937, Found: 185.0938.

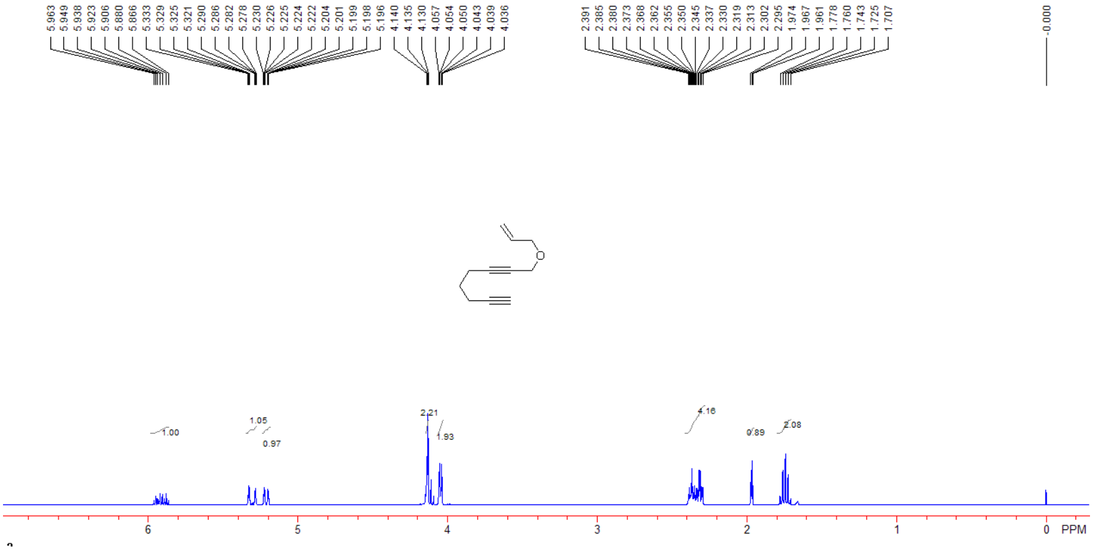

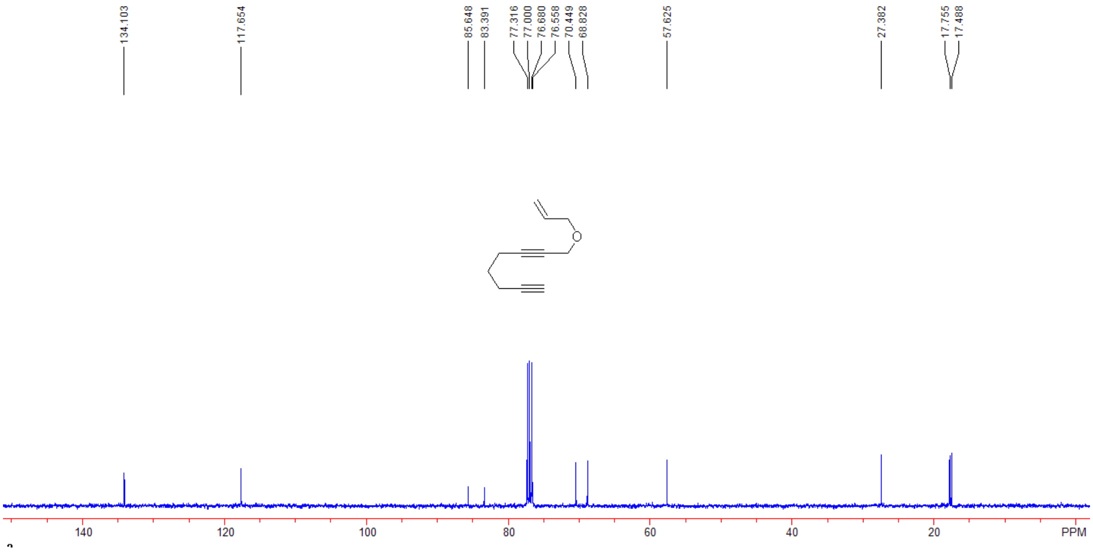

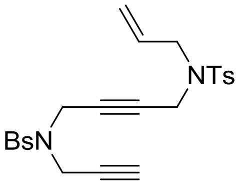

Compound 3g. 0.102 g, yield: 11%, white solid, Mp: 124-126 °C. ^{1}H NMR ($CDCl_3$, 400 MHz, TMS) δ 2.12 (t, *J* = 8.0 Hz, 1H, ≡CH), 2.45 (s, 3H, CH_3), 3.73 (d, *J* = 6.4 Hz, 2H, CH_2), 3.91 (s, 2H, CH_2), 3.92 (d, *J* = 2.0 Hz, 2H, CH_2), 3.99 (s, 2H, CH_2), 5.19 (d, *J* = 6.4 Hz, 1H, =CH_2), 5.23 (s, 1H, =CH_2), 5.63-5.73 (m, 1H, =CH), 7.33 (d, *J* = 8.0 Hz, 2H, Ar), 7.61-7.66 (m, 4H, Ar), 7.71 (d, *J* = 8.0 Hz, 2H, Ar). ^{13}C NMR ($CDCl_3$, 100 MHz, TMS) δ 21.5, 35.8, 36.1, 36.3, 49.2, 74.4, 75.6, 77.6, 79.0, 119.9, 127.1, 127.6, 128.2, 129.2, 129.6, 131.8, 132.2, 135.9, 137.1, 143.8. IR (CH_2Cl_2) ν 3283, 2923, 1597, 1574, 1430, 1390, 1348, 1159, 1191, 1009, 895, 760 cm^{-1}. MS (ESI) m/e 552.1 (M^++NH_4). HRMS (ESI) calcd. for $C_{23}H_{23}BrN_2O_4S_2$: 534.0283, Found: 534.0286.

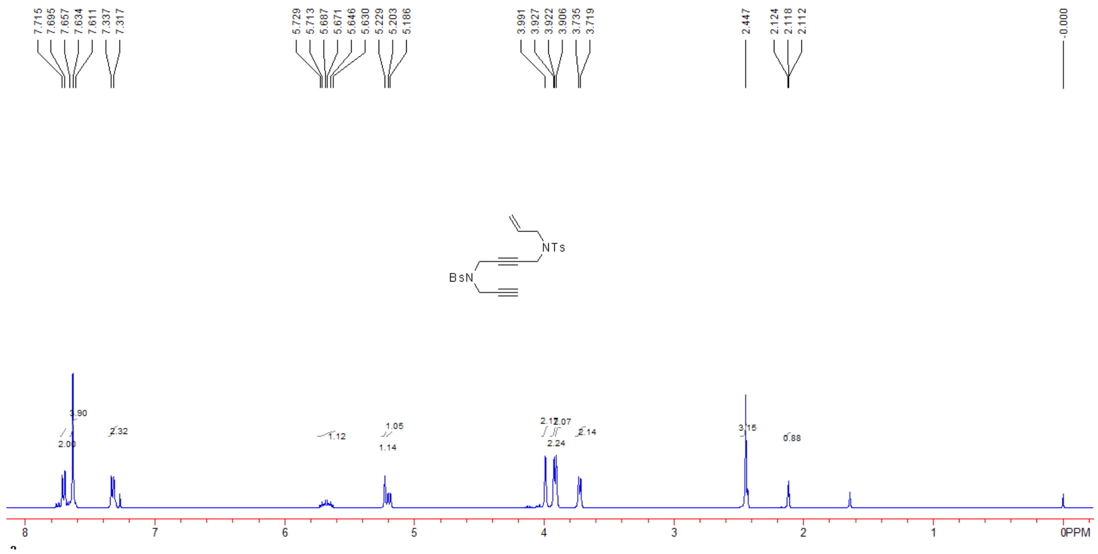

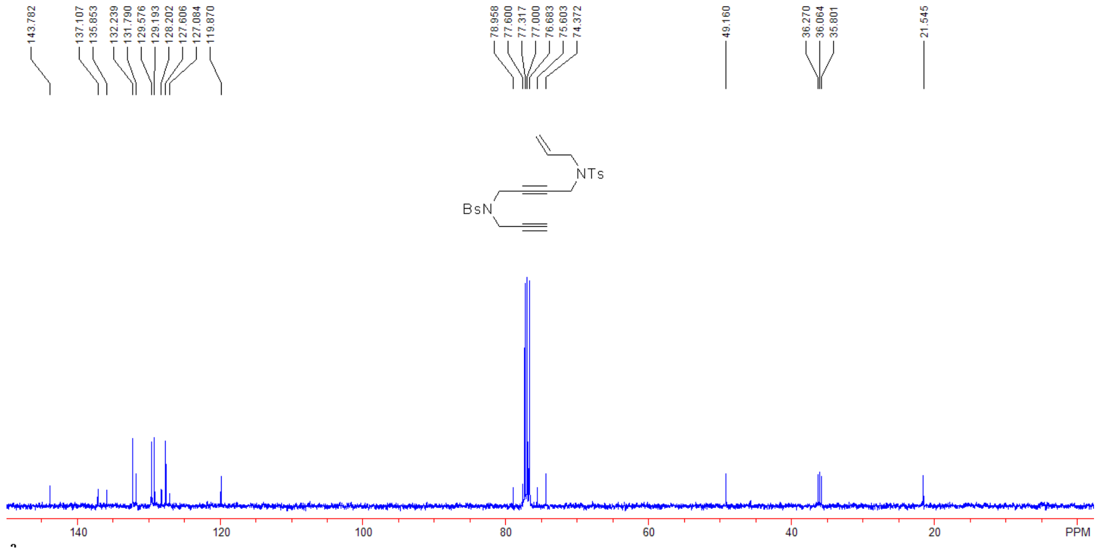

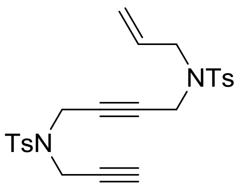

Compound 3h. 0.102 g, yield: 11%, white solid, Mp: 91-93 °C. ^{1}H NMR ($CDCl_3$, 400 MHz, TMS) δ 2.09 (t, *J* = 2.4 Hz, 1H, ≡CH), 2.43 (s, 3H, CH_3), 2.45 (s, 3H, CH_3), 3.72 (d, *J* = 6.4 Hz, 2H, CH_2), 3.90 (s, 2H, CH_2), 3.97 (s, 2H, CH_2), 5.19 (d, *J* = 5.6 Hz, 2H, =CH_2), 5.22 (s, 1H, =CH_2), 5.62-5.72 (m, 1H, =CH), 7.29 (d, *J* = 8.0 Hz, 2H, Ar), 7.32 (d, *J* = 8.0 Hz, 2H, Ar), 7.64 (d, *J* = 8.0 Hz, 2H, Ar), 7.69 (d, *J* = 8.0 Hz, 2H, Ar). ^{13}C NMR ($CDCl_3$, 100 MHz, TMS) δ 21.5, 21.9, 35.8, 36.0, 36.1, 49.1, 74.0, 76.0, 78.0, 78.5, 119.9, 127.6, 127.7, 129.5, 129.6, 131.8, 135.0, 135.9, 143.8, 144.1. IR (CH_2Cl_2) ν 3284, 1643, 1598, 1427, 1320, 1305, 1154, 1092, 922, 812 cm^{-1}. MS (ESI) m/e 488.2 ($M^{+}+NH_4$). HRMS (ESI) calcd. for $C_{24}H_{26}N_2O_4S_2$: 470.1334, Found: 470.1329.

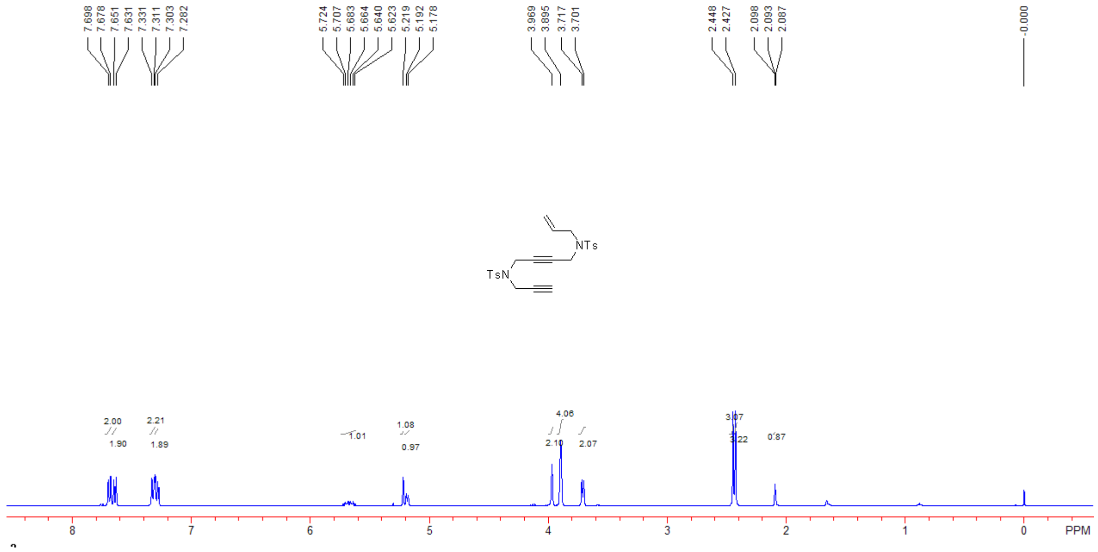

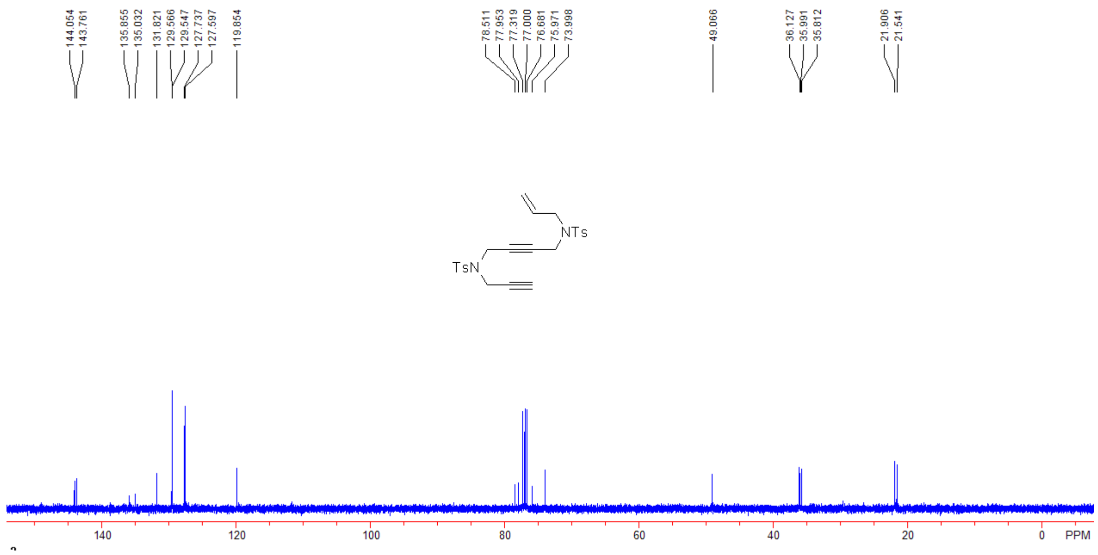

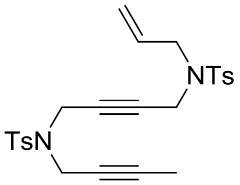

Compound 3i. 0.311 g, yield: 64%, white solid, Mp: 98-100 ^{o}C. ^{1}H NMR ($CDCl_3$, 400 MHz, TMS) δ 1.61 (t, *J* = 2.4 Hz, 1H, ≡CH), 2.42 (s, 3H, CH_3), 2.45 (s, 3H, CH_3), 3.71 (d, *J* = 2.0 Hz, 2H, CH_2), 3.83 (d, *J* = 2.4 Hz, 2H, CH_2), 3.86 (s, 2H, CH_2), 3.97 (s, 2H, CH_2), 5.18-5.22 (m, 2H, $=CH_2$), 5.61-5.73 (m, 1H, =CH), 7.28 (d, *J* = 8.0 Hz, 2H, Ar), 7.32 (d, *J* = 8.0 Hz, 2H, Ar), 7.64 (d, *J* = 8.0 Hz, 2H, Ar), 7.69 (d, *J* = 8.0 Hz, 2H, Ar). ^{13}C NMR ($CDCl_3$, 100 MHz, TMS) δ 3.3, 21.49, 21.52, 35.9, 36.1, 36.5, 49.0, 71.1, 78.1, 78.4, 81.9, 119.9, 127.1, 127.6, 127.8, 129.4, 129.5, 131.8, 135.2, 135.8, 143.8. IR (CH_2Cl_2) ν 2963, 2919, 1597, 1494, 1430, 1347, 1260, 1159, 1091, 1018, 800 cm^{-1}. MS (ESI) m/e 502.2 (M^++NH_4). HRMS (ESI) calcd. for $C_{25}H_{28}N_2O_4S_2$: 484.1490, Found: 484.1497.

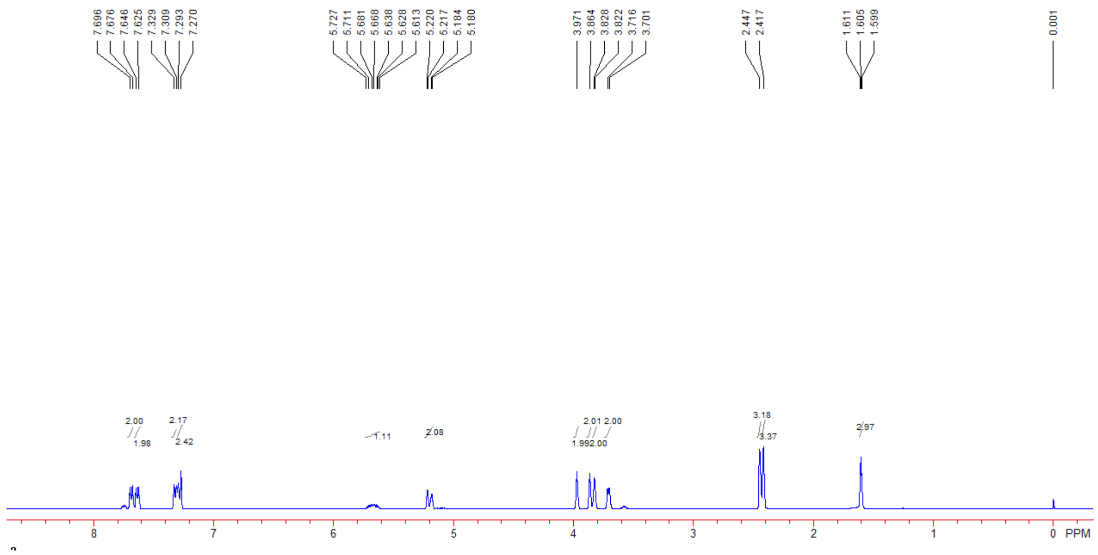

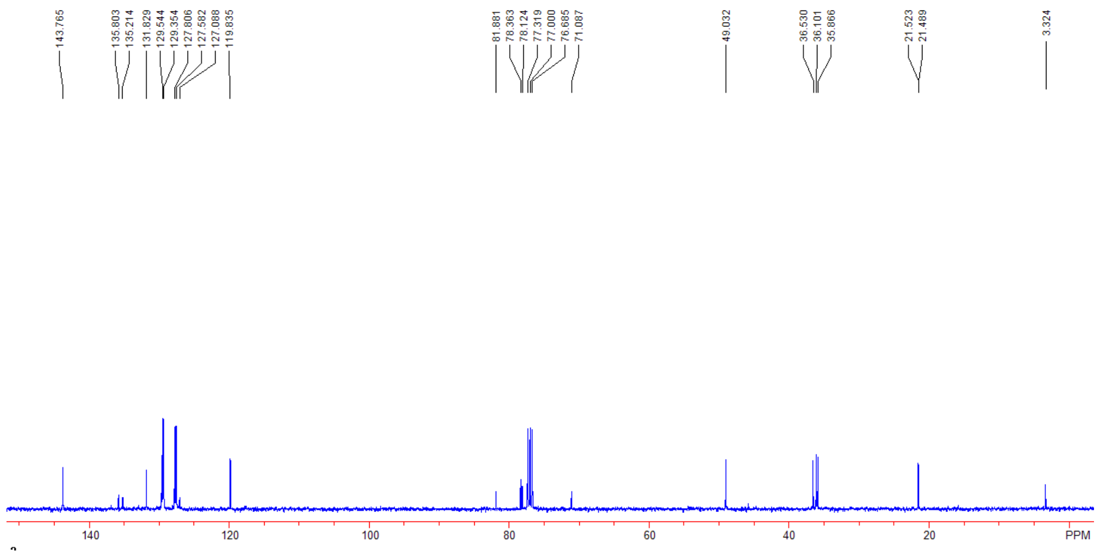

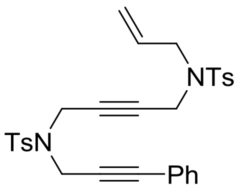

Compound 3j. 0.311 g, yield: 64%, white solid, Mp: 100-102 °C. ^{1}H NMR ($CDCl_3$, 400 MHz, TMS) δ 2.35 (s, 3H, CH_3), 2.40 (s, 3H, CH_3), 3.74 (d, J = 3.2 Hz, 2H, CH_2), 3.92 (s, 2H, CH_2), 4.01 (s, 2H, CH_2), 4.13 (s, 2H, CH_2), 5.19 (d, J = 3.2 Hz, 1H, =CH_2), 5.23 (d, J = 10.4 Hz, 1H, =CH_2), 5.64-5.74 (m, 1H, =CH), 7.11 (d, J = 8.0 Hz, 2H, Ar), 7.24-7.30 (m, 7H, Ar), 7.68 (d, J = 8.8 Hz, 2H, Ar), 7.70 (d, J = 8.8 Hz, 2H, Ar). ^{13}C NMR ($CDCl_3$, 100 MHz, TMS) δ 21.4, 21.5, 35.9, 36.4, 36.9, 49.1, 78.2, 78.4, 80.9, 85.9, 119.9, 121.8, 127.1, 127.6, 127.8, 128.2, 128.6, 129.5, 129.6, 129.7, 131.4, 131.8, 135.0, 135.7, 143.8, 144.0. IR (CH_2Cl_2) ν 1597, 1490,

1442, 1347, 1329, 1159, 1092, 898, 659 cm^{-1}. MS (ESI) m/e 564.2 ($M^{+}+NH_4$). HRMS (ESI) calcd. for $C_{30}H_{30}N_2O_4S_2$: 546.1647, Found: 546.1647.

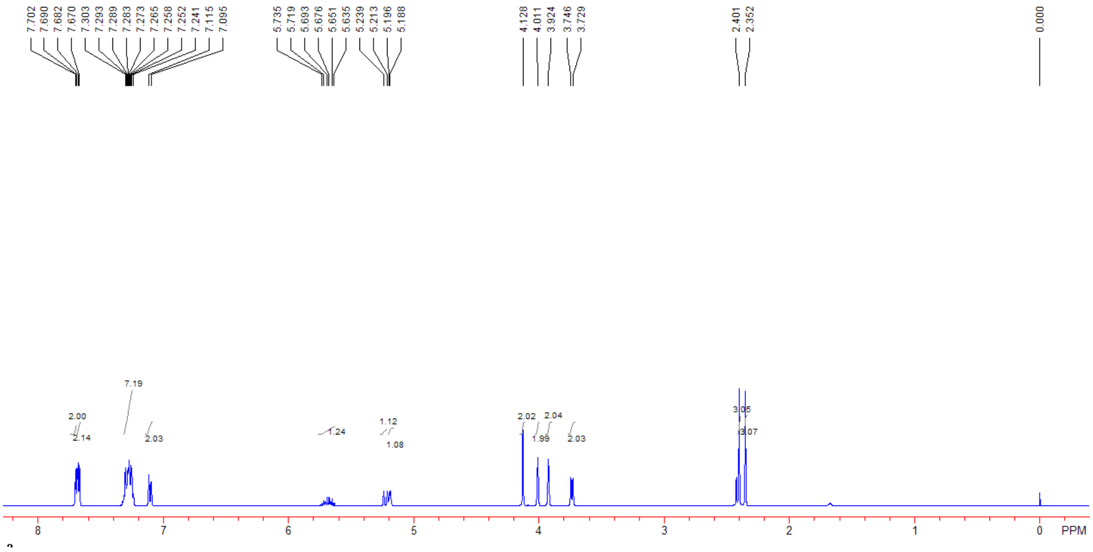

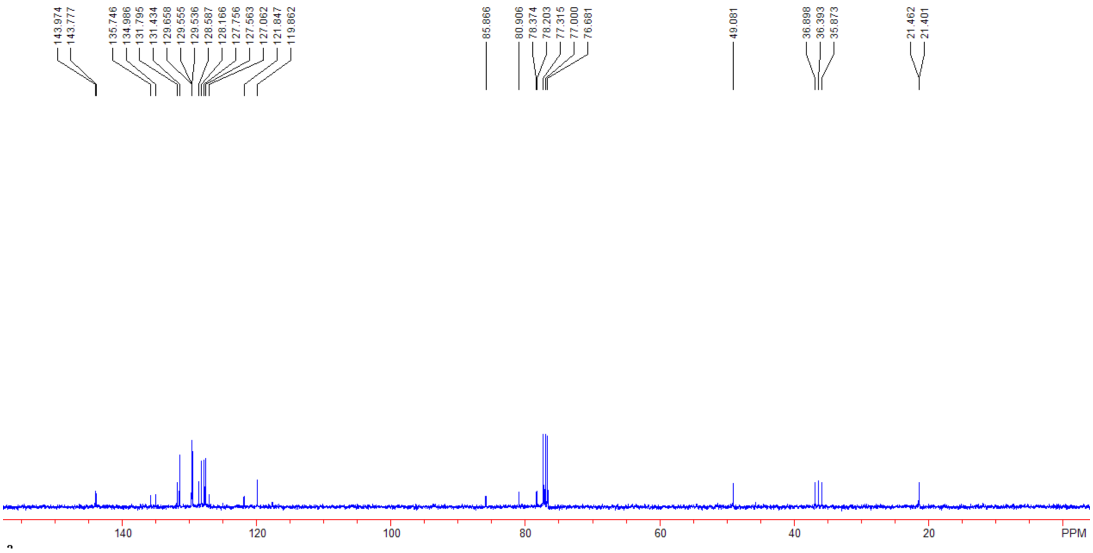

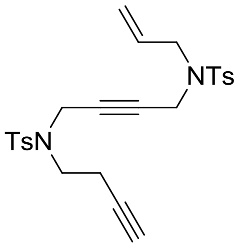

Compound 3k. 0.319 g, yield: 66%, white solid, Mp: 114-116 °C. ^{1}H NMR ($CDCl_3$, 400 MHz, TMS) δ 2.00 (t, J = 2.8 Hz, 1H, ≡CH), 2.37 (td, J_1 = 7.2 Hz, J_2 = 2.8 Hz, 2H, CH_2), 2.42 (s, 3H, CH_3), 2.44 (s, 3H, CH_3), 3.16 (t, J = 7.2 Hz, 2H, CH_2), 3.64 (d, J = 6.4 Hz, 2H, CH_2), 3.89 (t, J = 1.6 Hz, 2H, CH_2), 3.95 (t, J = 1.6 Hz, 2H, CH_2), 5.10-5.19 (m, 2H, =CH_2), 5.58-5.68 (m,

1H, =CH), 7.29 (d, *J* = 8.0 Hz, 2H, Ar), 7.31 (d, *J* = 8.0 Hz, 2H, Ar), 7.64 (d, *J* = 8.0 Hz, 2H, Ar), 7.67 (d, *J* = 8.0 Hz, 2H, Ar). ^{13}C NMR ($CDCl_3$, 100 MHz, TMS) δ 18.7, 21.4, 35.7, 37.2, 45.2, 48.9, 70.4, 78.28, 78.33, 80.5, 119.6, 127.4, 127.5, 129.4, 129.5, 131.7, 135.6, 135.8, 143.6, 143.7. IR (CH_2Cl_2) ν 3284, 1597, 1494, 1431, 1346, 1159, 1093, 931, 845 cm^{-1}. MS (ESI) m/e 502.2 (M^++NH_4). HRMS (ESI) calcd. for $C_{25}H_{28}N_2O_4S_2$: 484.1490, Found: 484.1497.

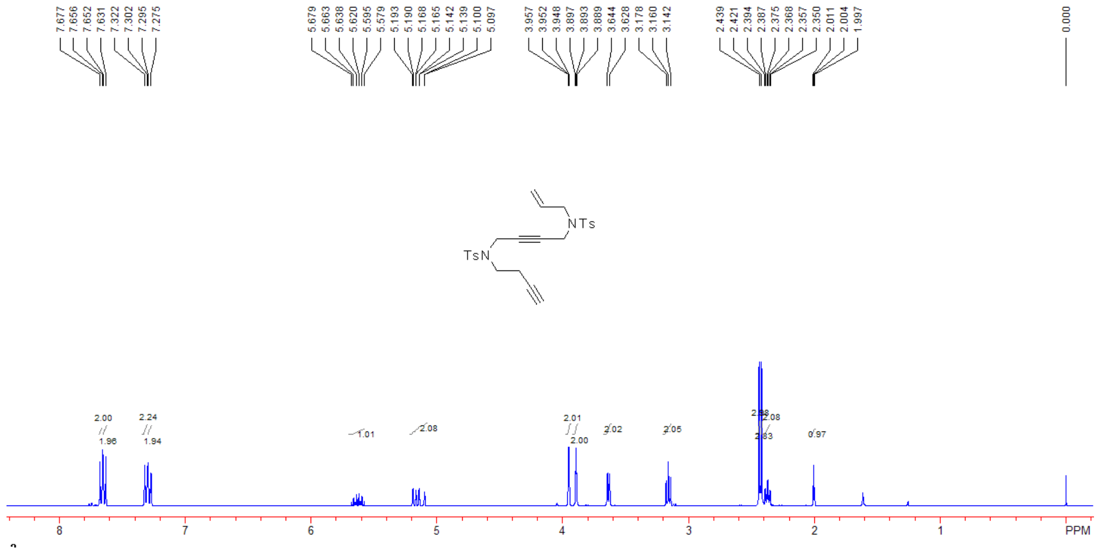

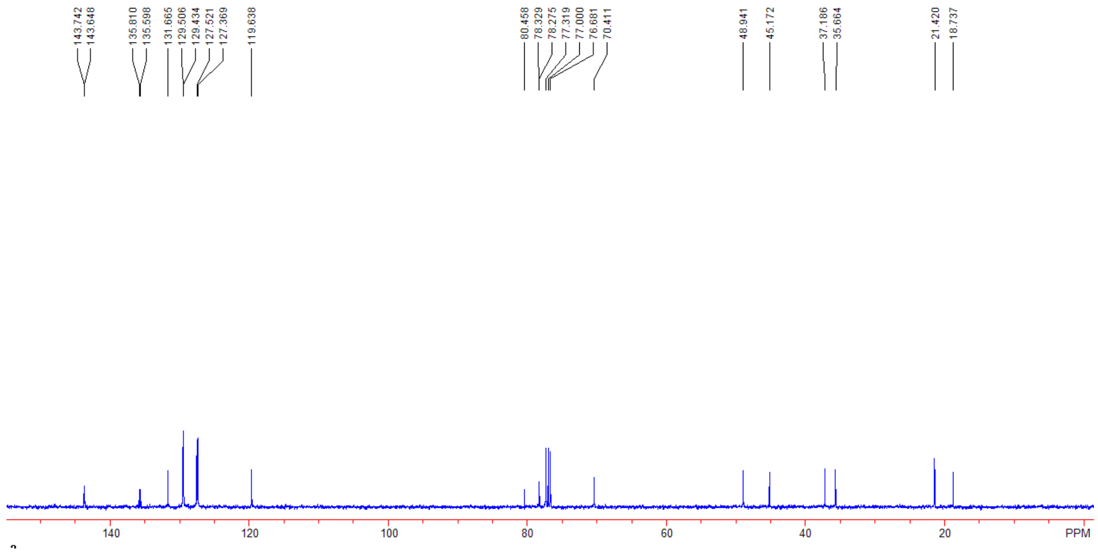

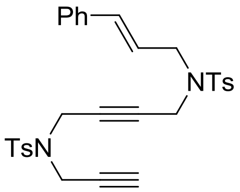

Compound 3m. 0.256 g, yield: 69%, white solid, Mp: 108-110 °C. ^{1}H NMR ($CDCl_3$, 400 MHz, TMS) δ 2.10 (t, *J* = 2.4 Hz, 1H, ≡CH), 2.39 (s, 3H, CH_3), 2.45 (s, 3H, CH_3), 3.88 (d, *J* = 6.8 Hz, 2H, CH_2), 3.90 (d, *J* = 2.4 Hz, 2H, CH_2), 3.92 (s, 2H, CH_2), 4.00 (s, 2H, CH_2), 5.98-6.06 (m, 1H, =CH), 6.49 (d, *J* = 16.0 Hz, 1H, =CH), 7.25-7.35 (m, 9H, Ar), 7.64 (d, *J* = 8.0 Hz, 2H, Ar), 7.72 (d, *J* = 8.0 Hz, 2H, Ar). ^{13}C NMR ($CDCl_3$, 100 MHz, TMS) δ 21.5, 21.6, 35.9, 36.0, 36.2, 48.7, 74.1, 76.0, 78.1, 78.5, 122.7, 126.4, 126.5, 127.2, 127.65, 127.73, 128.1, 128.5, 128.6, 129.56, 129.60, 129.7, 134.8, 135.0, 135.8, 135.9, 143.8, 144.1. IR (CH_2Cl_2) ν 3281, 1597, 1495, 1448, 1348, 1332, 1301, 1161, 1092, 971, 749, 662 cm^{-1}. MS (ESI) m/e 564.2 ($M^{+}+NH_4$). HRMS (ESI) calcd. for $C_{30}H_{30}N_2O_4S_2$: 546.1647, Found: 546.1625.

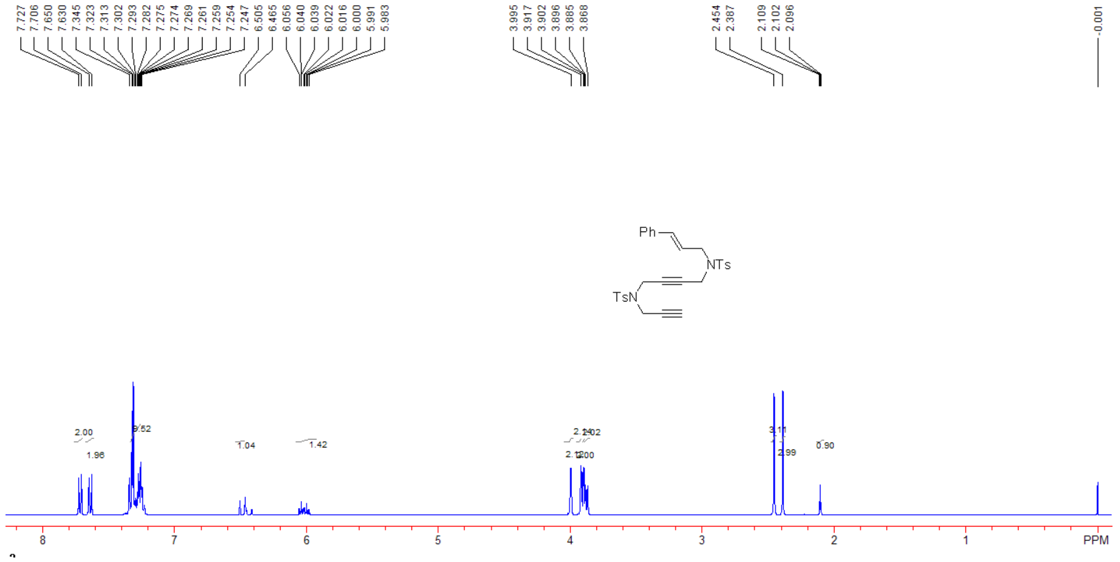

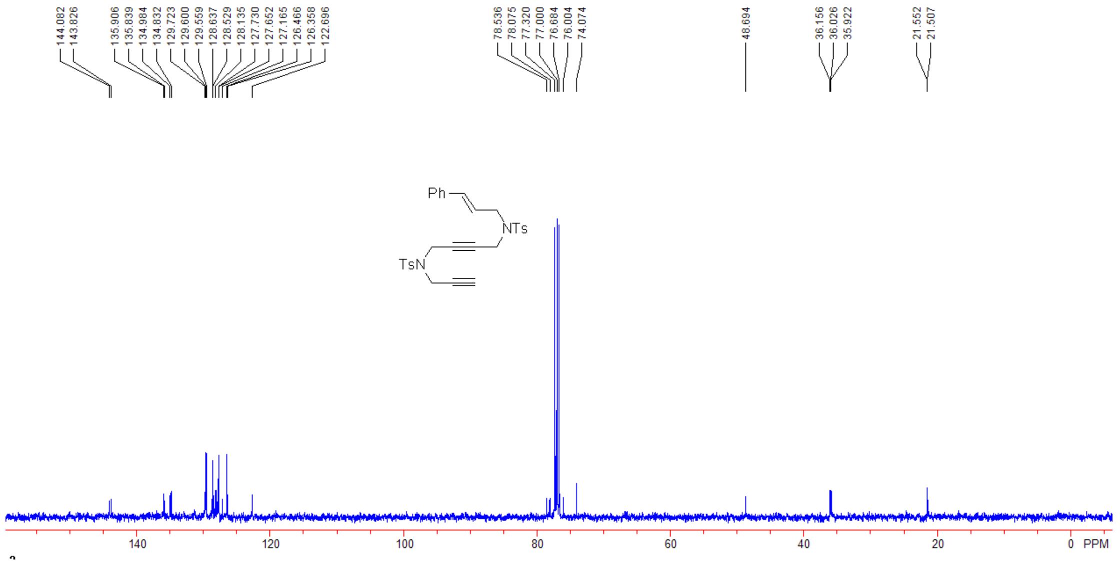

Synthesis of [D]-1e:

To the solution of compound **1e** (0.2 mmol) in THF (5.0 mL) was added ^{n}BuLi (0.48 mmol, 2.5 M in THF) within 10 min at -78 ^{o}C under argon. The resulting solution was allowed to stir at -78 ^{o}C for 0.5 h before D_2O (1.0 mL) was added in one portion. Consequently, the reaction mixture was allowed to stir at -78 ^{o}C for 1 h and then the reaction solution was naturally warmed up to room temperature to stir for another 1 h. Then, extracted with CH_2Cl_2, dried over anhydrous Na_2SO_4, filtered, the organic phase was purified by flash column chromatography on silica gel to give the desired product **[D]-1e** in 96% yield (PE/EA: 4:1).

Synthesis of [D]-3h:

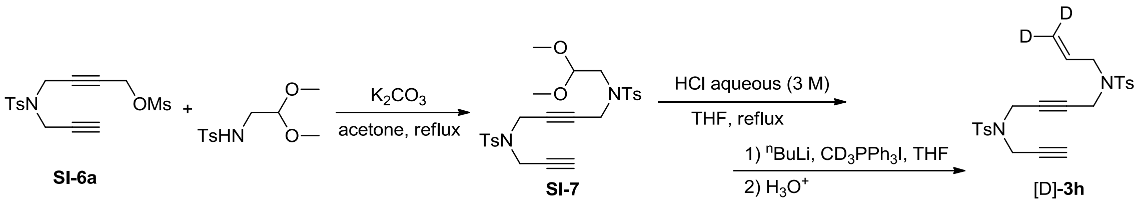

Synthesis of SI-7:

The corresponding compound **SI-6** (2.0 mmol), N-(2,2-dimethoxyethyl)-4-methylbenzenesulfonamide (2.0 mmol), K_2CO_3 (3.0 mmol) and the solvent acetone (20 mL) were added into a 50 mL flask. Then, the flask was heated to reflux for 8 h. Finally, the suspension was filtered off and the organic phase was purified by flash column chromatography on silica gel to give the desired product **SI-7** (PE/EA: 10:1~4:1).

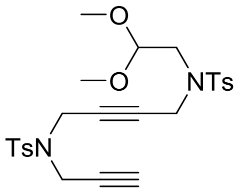

Compound SI-7. 0.996 g, yield: 96%, colorless oil. ^{1}H NMR ($CDCl_3$, 400 MHz, TMS) δ 2.08 (t, J = 2.4 Hz, 1H, ≡CH), 2.43 (s, 3H, CH_3), 2.44 (s, 3H, CH_3), 3.14 (d, J = 5.2 Hz, 2H, CH_2), 3.40 (s, 3H, CH_3), 3.86 (d, J = 2.4 Hz, 2H, CH_2), 3.89 (s, 2H, CH_2), 4.14 (t, J = 2.0 Hz, 2H, CH_2), 7.29 (d, J = 8.1 Hz, 2H, Ar), 7.31 (d, J = 8.1 Hz, 2H, Ar), 7.63 (d, J = 8.0 Hz, 2H, Ar), 7.69 (d, J = 8.0 Hz, 2H, Ar). ^{13}C NMR ($CDCl_3$, 100 MHz, TMS) δ 21.5, 35.9, 36.1, 38.2, 44.4,

47.6, 54.5, 54.7, 74.0, 75.9, 77.7, 79.1, 102.4, 104.4, 127.0, 127.5, 127.7, 129.6, 129.7, 135.0, 135.8, 143.5, 143.8, 144.0. IR (CH_2Cl_2) ν 3275, 2931, 2828, 1597, 1494, 1437, 1347, 1158, 1127, 1090, 747 cm^{-1}. MS (ESI) m/e 536.2 (M^++NH_4). HRMS (ESI) calcd. for $C_{25}H_{30}N_2O_6S_2$: 518.1545, Found: 518.1553.

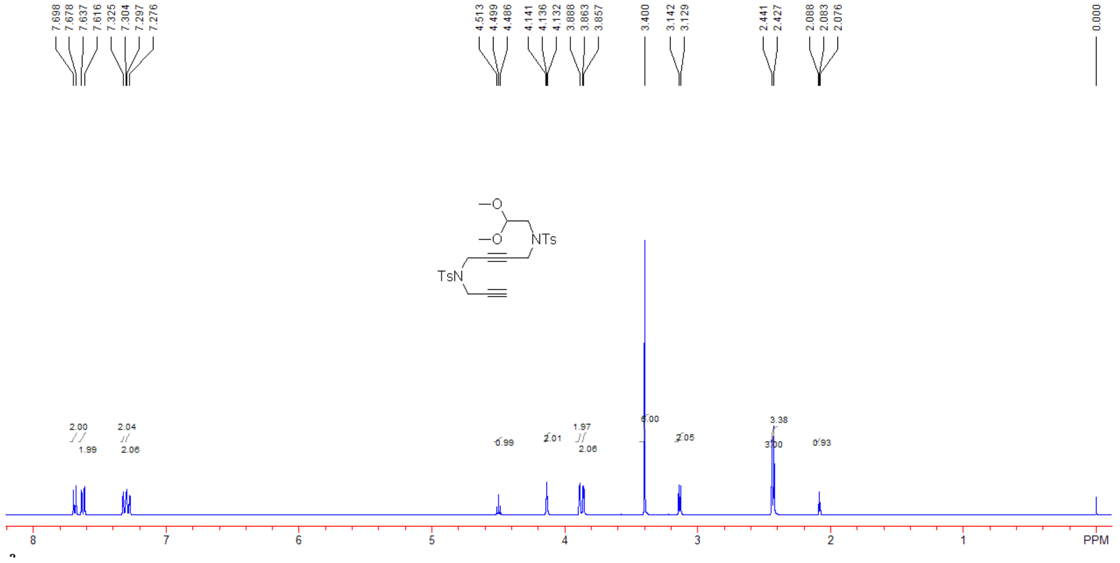

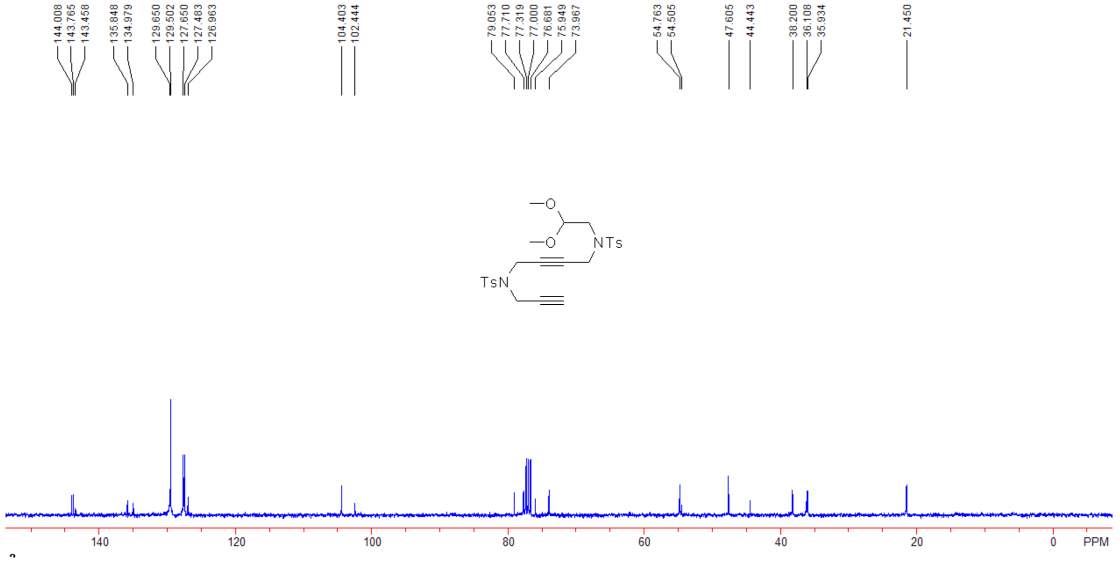

Synthesis of [D]-3h:

To the solution of **SI-7** (5 mmol) in THF (10 mL) was added HCl aqueous solution (3.0 M) (5.0 mL). The resulting solution was heated to reflux for 24 h. Then, extracted with CH_2Cl_2, dried over anhydrous Na_2SO_4, filtered, the organic phase was evaporated to dryness without purification.

To the solution of CD_3PPh_3I (0.24 mmol) in THF (5.0 mL) was added ^{n}BuLi (0.24 mmol, 2.5 M in THF) within 10 min at -78 °C under argon. Then, the mixture was allowed to stir at -78

°C for 0.5 h before the above crude product (0.2 mmol) in THF (5.0 mL) was added. The resulting solution was allowed to stir at -78 °C for 1 h and then the solution was naturally warmed up to room temperature to stir for another 1 h. Then, extracted with EtOAc, dried over anhydrous Na_2SO_4, filtered, the organic phase was purified by flash column chromatography on silica gel to give the desired product **[D]-3h** in 85% yield (PE/EA: 4:1).

Representative procedure for the ruthenium-catalyzed [2+2+2] intramolecular cycloaddition of triynes:

To a flame dried Schlenk tube was added substrate **1** (0.2 mmol), Grubbs-I catalyst (10 mol%) and the solvent CH_2Cl_2 (2.0 mL). The resulting solution was allowed to stir at room temperature for 12 h. Then, the reaction mixture was evaporated to dryness and the residue was purified by flash silica gel column chromatography (PE:EA = 10:1~4:1).

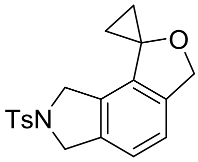

Compound 2a. 57 mg, yield: 80%, white solid, 217-219 °C. ^{1}H NMR ($CDCl_3$, 300 MHz, TMS) δ 1.02 (dd, J_1 = 8.4 Hz, J_2 = 6.0 Hz, 2H, CH_2), 1.23 (dd, J_1 = 8.4 Hz, J_2 = 6.0 Hz, 2H, CH_2), 2.41 (s, 3H, CH_3), 4.40 (s, 2H, CH_2), 4.56 (s, 2H, CH_2), 5.11 (s, 2H, CH_2), 7.03 (d, J = 7.8 Hz, 1H, Ar), 7.09 (d, J = 7.8 Hz, 1H, Ar), 7.32 (d, J = 8.1 Hz, 2H, Ar), 7.75 (d, J = 8.1 Hz, 2H, Ar). ^{13}C NMR ($CDCl_3$, 75 MHz, TMS) δ 11.2, 21.5, 50.5, 52.9, 68.3, 71.6, 120.6, 120.9, 126.8, 127.5, 129.9, 133.4, 136.3, 136.5, 139.3, 143.8. IR (CH_2Cl_2) ν 2956, 2923, 2855, 1597, 1493, 1465, 1345, 1163, 1098, 680 cm^{-1}. MS (ESI) m/e 342.1 (M^++1). HRMS (ESI) calcd. for $C_{19}H_{19}NO_3S$: 341.1086, Found: 341.1083.

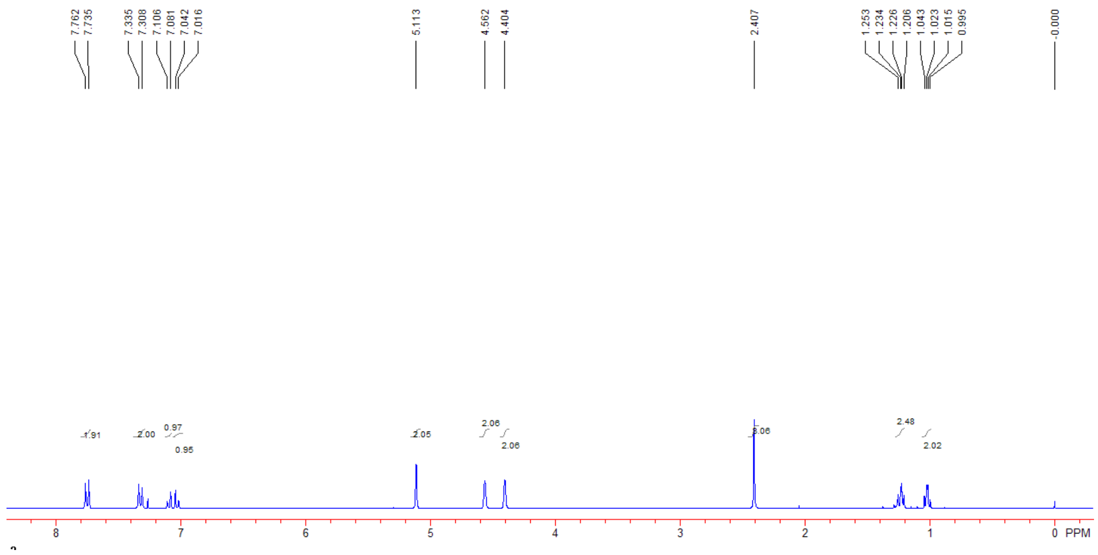

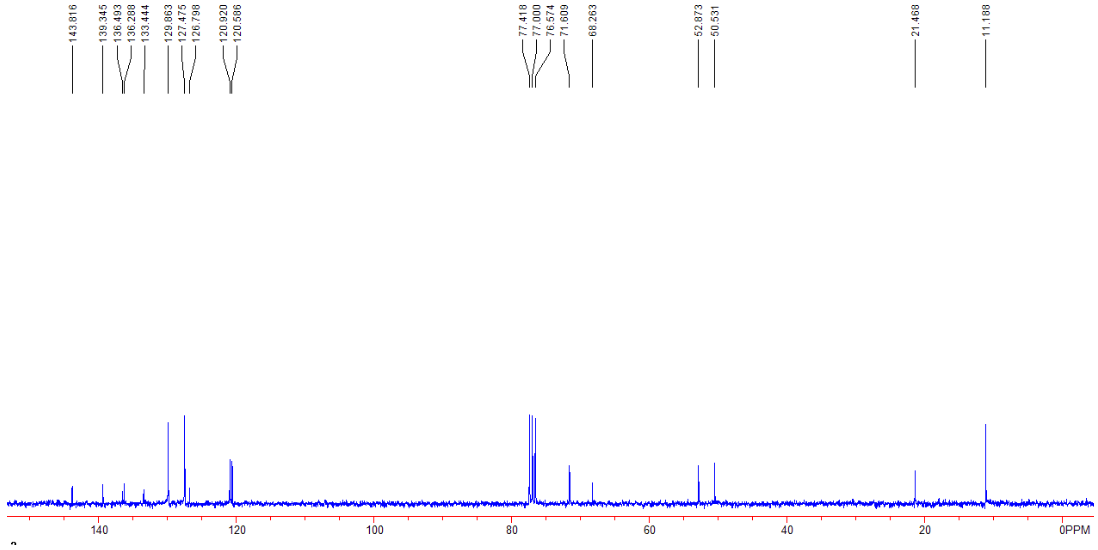

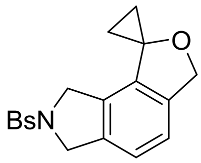

Compound 2b. 69 mg, yield: 85%, white solid, 245-247 °C. ^{1}H NMR ($CDCl_3$, 400 MHz, TMS) δ 1.02 (dd, J_1 = 8.0 Hz, J_2 = 6.0 Hz, 2H, CH_2), 1.24 (dd, J_1 = 8.0 Hz, J_2 = 6.0 Hz, 2H, CH_2), 4.41 (s, 2H, CH_2), 4.57 (s, 2H, CH_2), 5.12 (s, 2H, CH_2), 7.04 (d, J = 8.0 Hz, 1H, Ar), 7.11 (d, J = 8.0 Hz, 1H, Ar), 7.66-7.74 (m, 4H, Ar). ^{13}C NMR ($CDCl_3$, 100 MHz, TMS) δ 11.2, 50.6, 52.9, 68.3, 71.6, 120.8, 121.0, 126.5, 128.1, 128.9, 132.6, 135.7, 136.0, 136.6, 139.6. IR (CH_2Cl_2) ν 2953, 2916, 2860, 1574, 1466, 1386, 1341, 1174, 1341, 1151, 1067, 744 cm^{-1}. MS (ESI) m/e 406.0 (M^++1). HRMS (ESI) calcd. for $C_{18}H_{16}BrNO_3S$: 405.0034, Found: 405.0028.

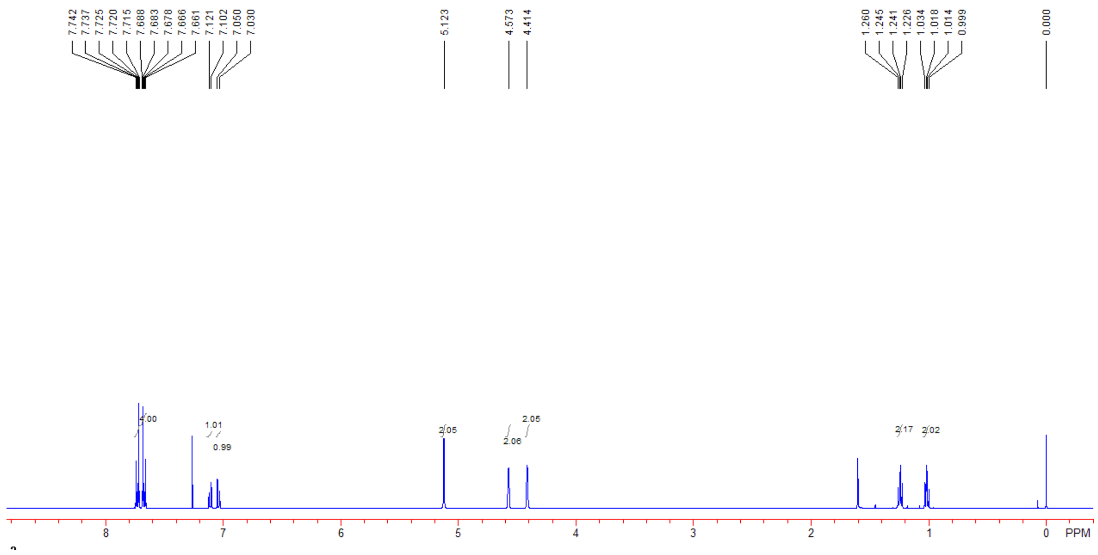

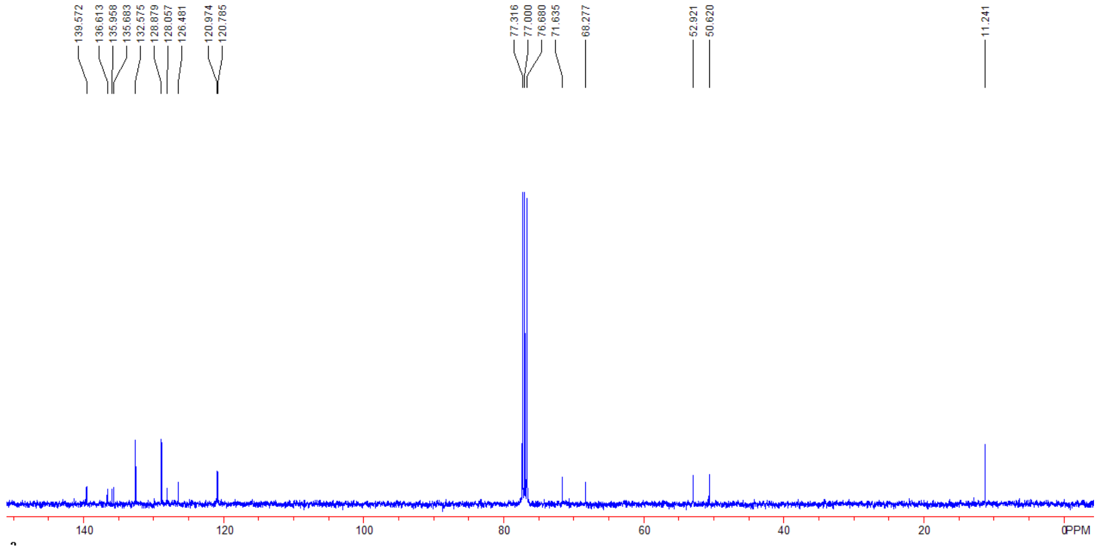

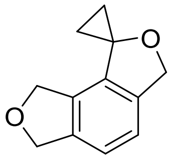

Compound 2c. 71 mg, yield: 86%, white solid, Mp: 130-132 °C . ^{1}H NMR ($CDCl_3$, 400 MHz, TMS) δ 1.00 (dd, J_1 = 8.0 Hz, J_2 = 6.0 Hz, 2H, CH_2), 1.24 (dd, J_1 = 8.0 Hz, J_2 = 6.0 Hz, 2H, CH_2), 4.93 (t, J = 2.0 Hz, 2H, CH_2), 5.06 (s, 2H, CH_2), 5.19 (s, 2H, CH_2), 7.10 (d, J = 8.0 Hz, 1H, Ar), 7.13 (d, J = 8.0 Hz, 1H, Ar). ^{13}C NMR ($CDCl_3$, 100 MHz, TMS) δ 11.2, 68.4, 70.4, 71.9, 72.9, 119.2, 120.1, 129.8, 135.5, 138.7, 139.3. IR (CH_2Cl_2) ν 3000, 2919, 2854, 1460, 1441, 1340, 1194, 1124, 1051, 1028, 904, 840 cm^{-1}. MS (ESI) m/e 206.1 (M^++NH_4). HRMS (ESI) calcd. for $C_{12}H_{12}O_2$: 188.0837, Found: 188.0846.

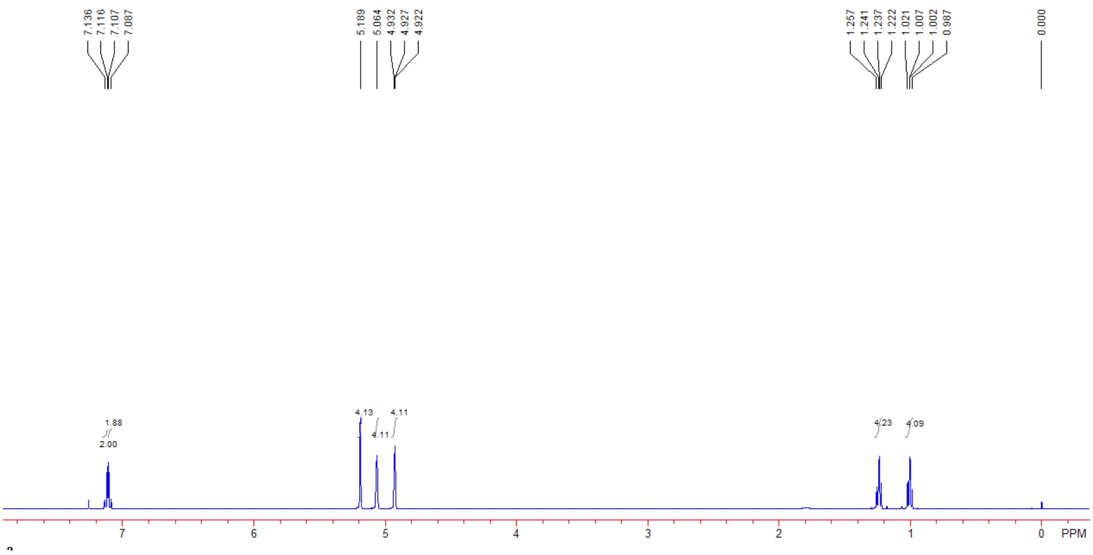

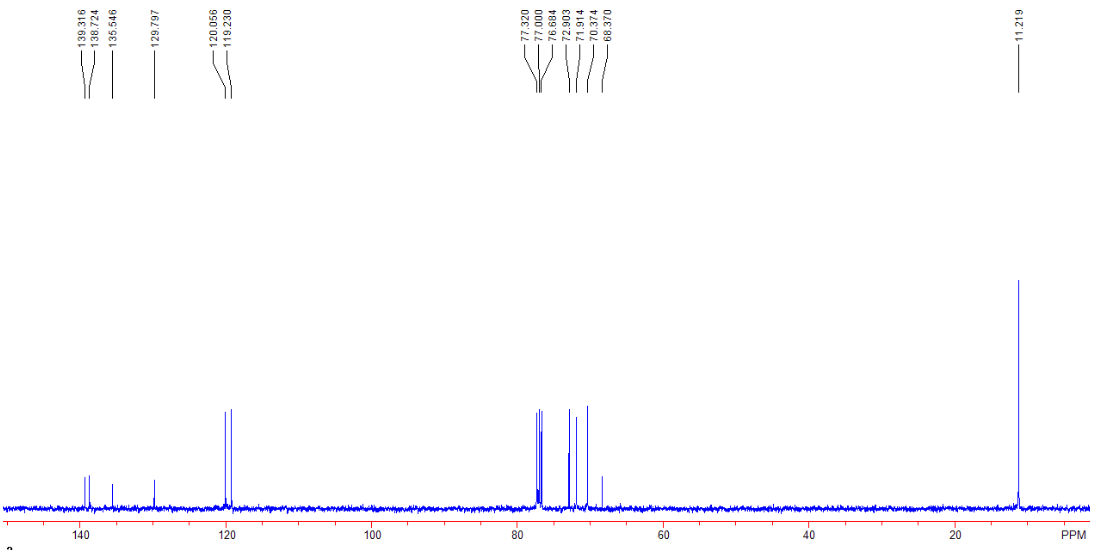

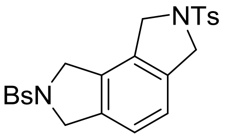

Compound 2d. 77 mg, yield: 72%, white solid, Mp: 252-254 °C. ^{1}H NMR ($CDCl_3$, 400 MHz, TMS) δ 2.41 (s, 3H, CH_3), 4.47 (s, 4H, CH_2), 4.58 (s, 4H, CH_2), 7.05 (d, J = 8.0 Hz, 1H, Ar), 7.07 (d, J = 8.0 Hz, 1H, Ar), 7.32 (d, J = 8.0 Hz, 2H, Ar), 7.65-7.67 (m, 2H, Ar), 7.718-7.723 (m, 2H, Ar), 7.75 (d, J = 8.0 Hz, 2H, Ar). ^{13}C NMR ($CDCl_3$, 100 MHz, TMS) δ 21.5, 52.1, 52.2, 53.4, 53.5, 122.1, 122.2, 127.5, 128.1, 128.9, 129.9, 130.5, 130.9, 132.6, 133.4, 135.6, 135.7, 136.3, 143.9. IR (CH_2Cl_2) ν 2957, 2924, 2852, 1574, 1459, 1345, 1157, 1098, 1067, 667 cm^{-1}. MS (ESI) m/e 550.0 (M^++NH_4). HRMS (ESI) calcd. for $C_{23}H_{21}BrN_2O_4S_2$: 532.0126,

Found: 532.0124.

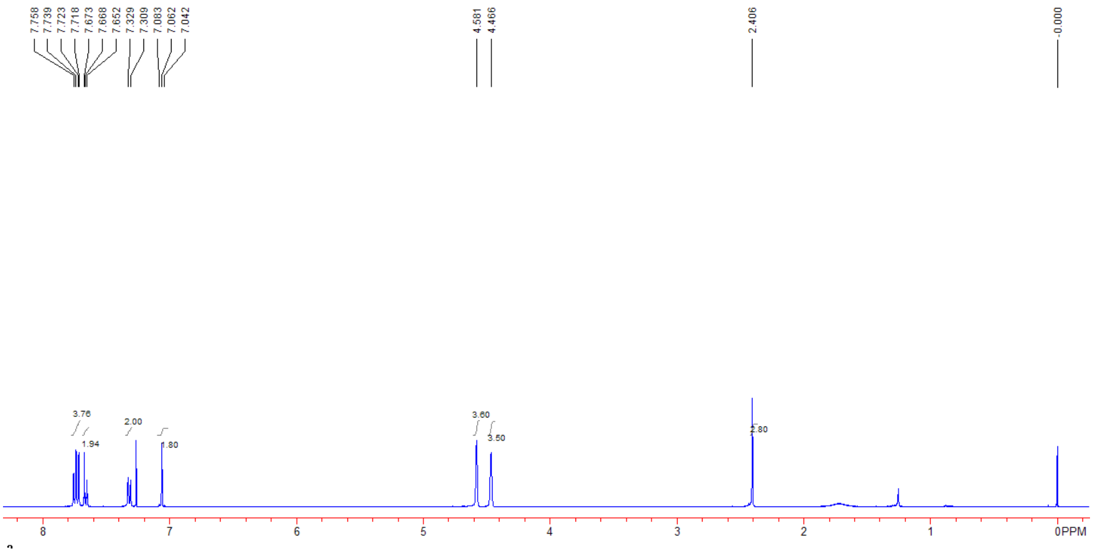

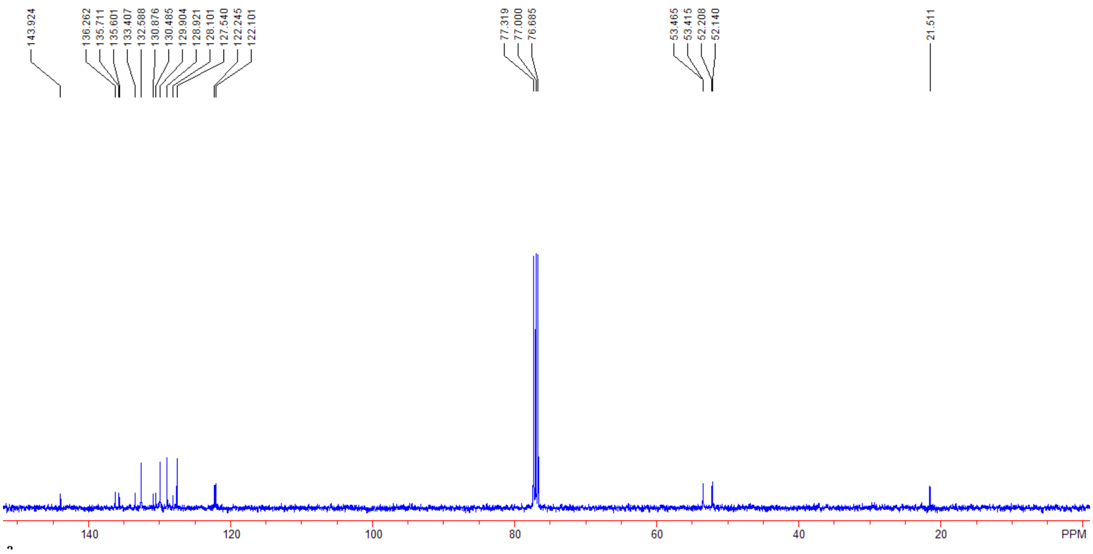

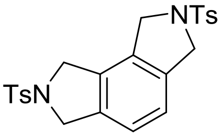

Compound 2e. 88 mg, yield: 94%, white solid, Mp: 264-266 °C. ^{1}H NMR ($CDCl_3$, 400 MHz, TMS) δ 2.40 (s, 6H, CH_3), 4.46 (s, 4H, CH_2), 4.57 (s, 4H, CH_2), 7.05 (s, 2H, Ar), 7.32 (d, J = 8.0 Hz, 4H, Ar), 7.75 (d, J = 8.0 Hz, 4H, Ar). ^{13}C NMR ($CDCl_3$, 100 MHz, TMS) δ 21.5, 52.1, 53.4, 122.0, 127.5, 129.9, 130.8, 133.4, 136.0, 143.9. IR (CH_2Cl_2) ν 2923, 2846, 1597, 1460, 1343, 1158, 1097, 1068, 812, 669 cm^{-1}. MS (ESI) m/e 486.1 (M^++NH_4). HRMS (ESI) calcd. for $C_{24}H_{24}N_2O_4S_2$: 468.1177, Found: 468.1159.

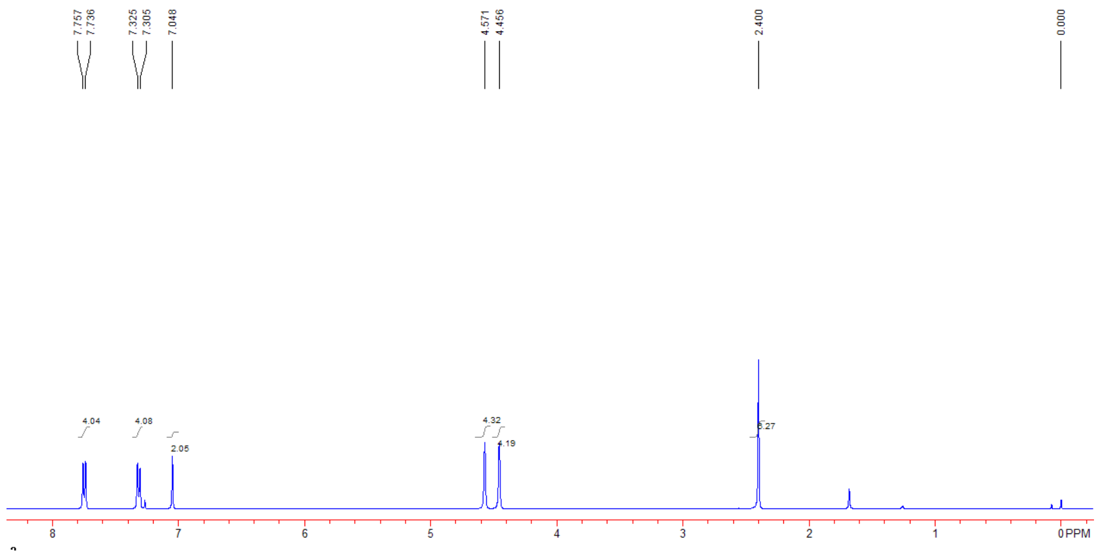

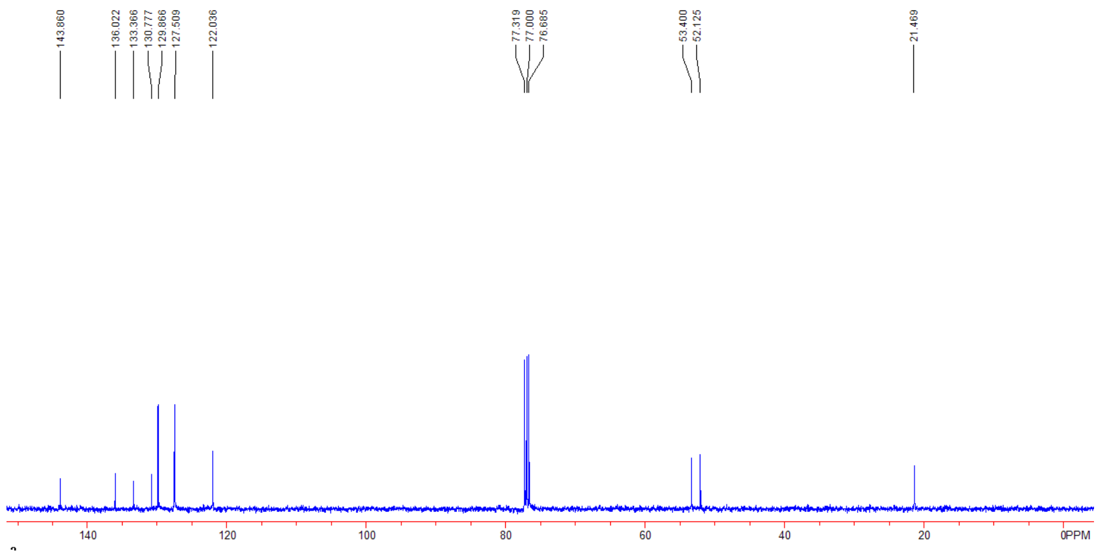

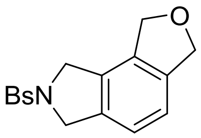

Compound 2f. 50 mg, yield: 66%, white solid, Mp: 206-208 °C. ^{1}H NMR ($CDCl_3$, 400 MHz, TMS) δ 4.52 (s, 2H, CH_2), 4.64 (s, 2H, CH_2), 4.99 (s, 2H, CH_2), 5.08 (s, 2H, CH_2), 7.10 (d, J = 7.2 Hz, 1H, Ar), 7.14 (d, J = 7.2 Hz, 1H, Ar), 7.67 (d, J = 8.0 Hz, 4H, Ar), 7.75 (d, J = 8.0 Hz, 4H, Ar). ^{13}C NMR ($CDCl_3$, 100 MHz, TMS) δ 52.4, 53.4, 72.0, 73.4, 120.6, 121.6, 128.0, 128.9, 129.1, 132.5, 133.7, 135.2, 135.8, 139.3. IR (CH_2Cl_2) ν 2924, 2854, 1574, 1468, 1389, 1345, 1167, 1098, 1067, 1053, 1008, 740 cm^{-1}. MS (ESI) m/e 380.0 (M^++H). HRMS (ESI) calcd. for $C_{16}H_{14}BrNO_3S$: 378.9878, Found: 378.9877.

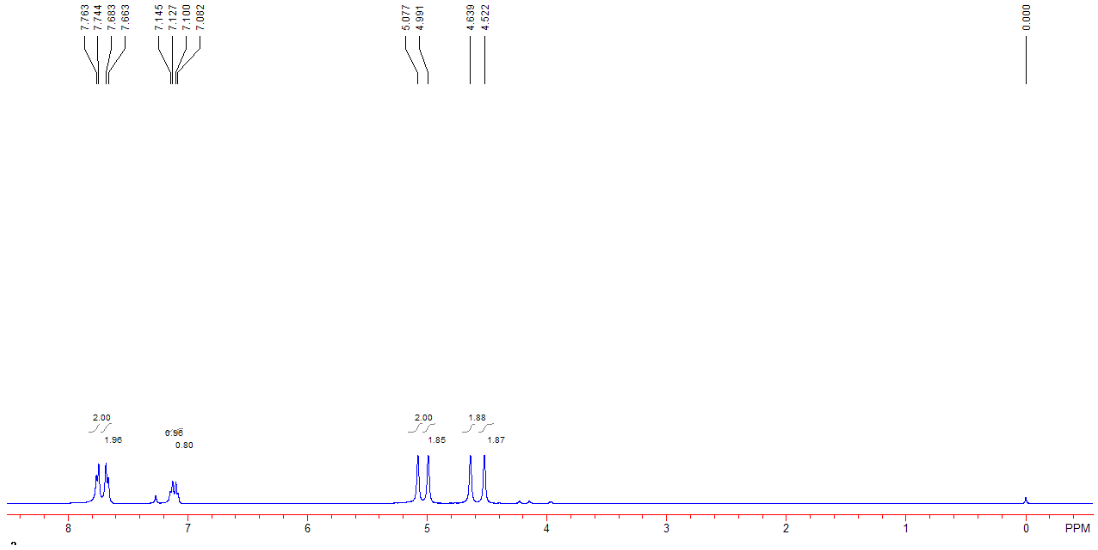

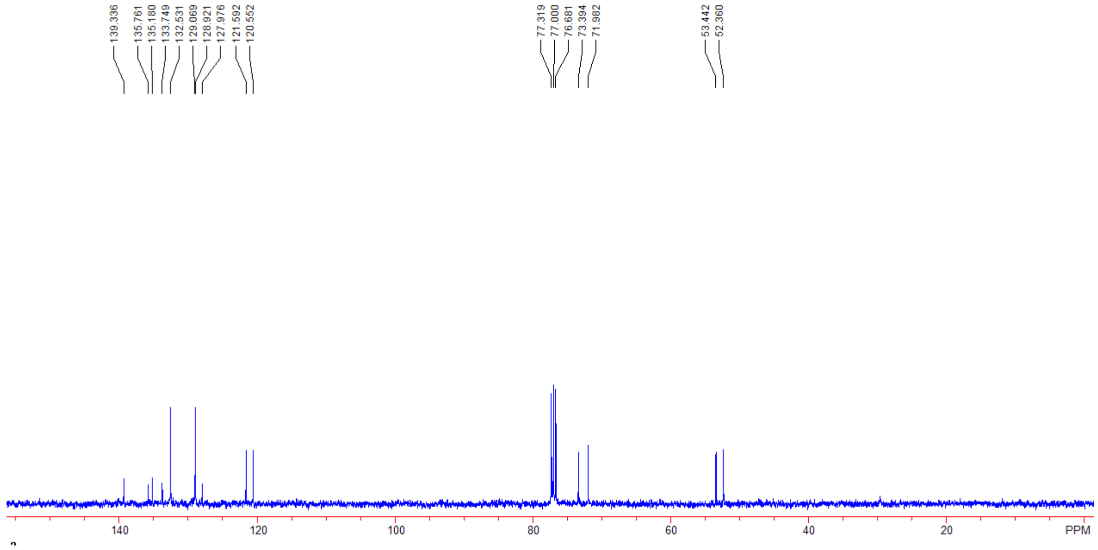

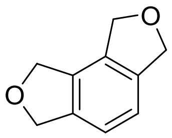

Compound 2g. 61 mg, yield: 95%, white solid, Mp: 112-114 ^{o}C. ^{1}H NMR ($CDCl_3$, 400 MHz, TMS) δ 5.02 (s, 4H, CH_2), 5.12 (s, 4H, CH_2), 7.14 (s, 2H, Ar). ^{13}C NMR ($CDCl_3$, 100 MHz, TMS) δ 72.1, 73.3, 119.8, 132.2, 138.6. IR (CH_2Cl_2) ν 2923, 2853, 1765, 1464, 1383, 1351, 1161, 1039, 899, 806 cm^{-1}. MS (%) m/e 162 (M^+, 62.87), 133 (28.22), 132 (21.79), 161 (29.82), 104 (100), 105 (77.12), 103 (34.66), 77 (34.45). HRMS (EI) calcd. for $C_{10}H_{10}O_2$: 162.0681, Found: 162.0676.

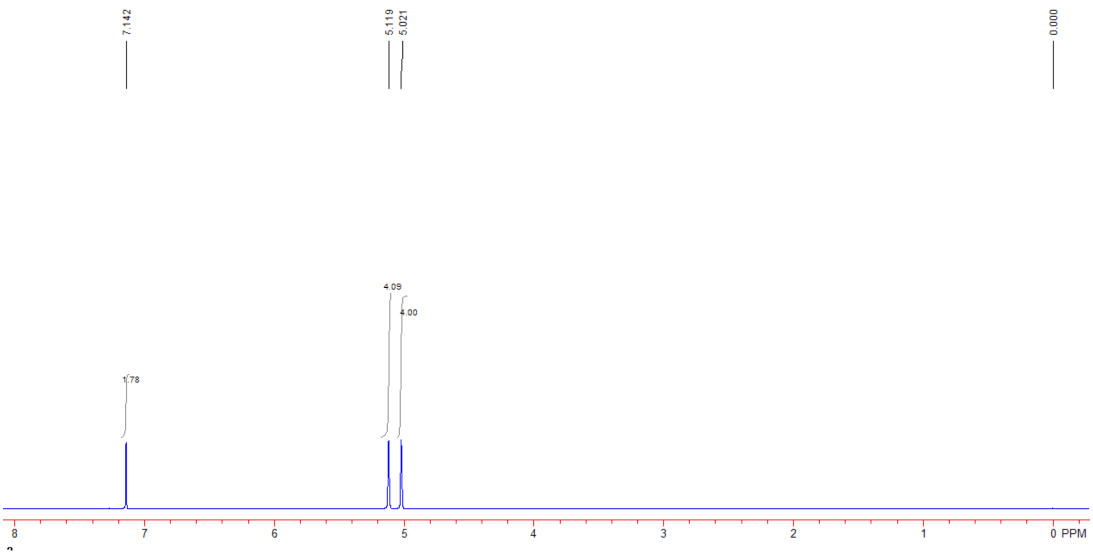

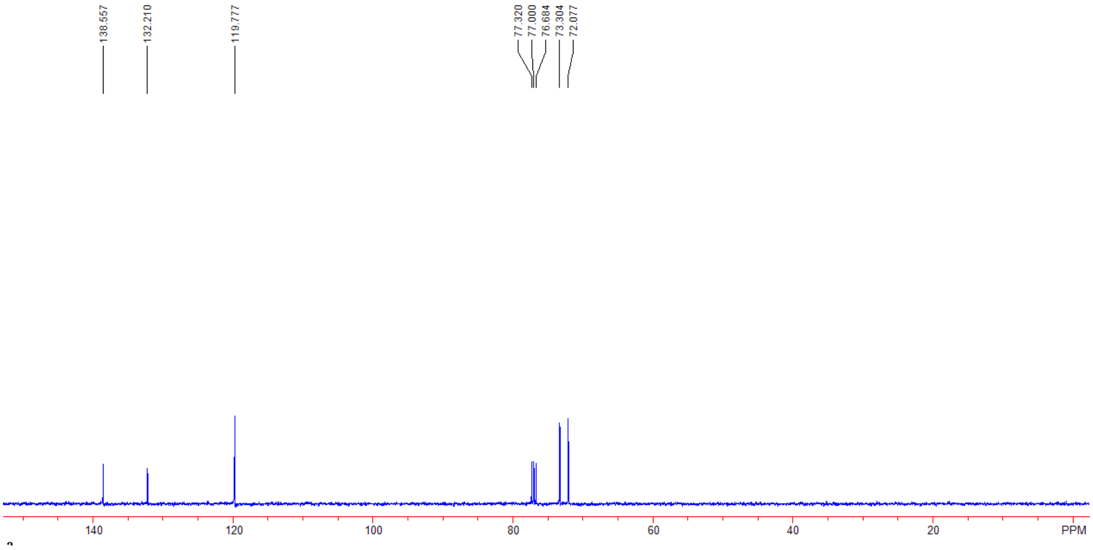

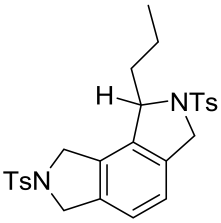

Compound 2h. 87 mg, yield: 83%, light yellow oil. ^{1}H NMR ($CDCl_3$, 400 MHz, TMS) δ 0.80-0.88 (m, 4H, CH_3 and CH_2), 1.24-1.36 (m, 2H, CH_2), 1.62-1.69 (m, 1H, CH_2), 2.09-2.15 (m, 1H, CH_2), 2.34 (s, 3H, CH_3), 2.41 (s, 3H, CH_3), 4.44-4.55 (m, 4H, CH_2), 4.60 (s, 2H, CH_2), 4.99 (s, 1H, CH), 6.99 (d, J = 8.0 Hz, 1H, Ar), 7.01 (d, J = 8.0 Hz, 1H, Ar), 7.22 (d, J = 8.4 Hz, 2H, Ar), 7.32 (d, J = 8.4 Hz, 2H, Ar), 7.68 (d, J = 8.0 Hz, 2H, Ar), 7.76 (d, J = 8.0 Hz, 2H, Ar). ^{13}C NMR ($CDCl_3$, 100 MHz, TMS) δ 13.8, 16.4, 21.3, 21.4, 36.2, 51.9, 53.1,

53.8, 64.9, 121.87, 121.90, 127.1, 127.4, 129.6, 129.8, 130.1, 133.4, 134.6, 134.7, 135.9, 136.1, 143.5, 143.8. IR (CH_2Cl_2) ν 2958, 2920, 2871, 1597, 1494, 1463, 1340, 1159, 1093, 1057, 813, 669 cm^{-1}. MS (%) m/e 467 (M^+-^{n}Pr, 100), 91 (77.03), 155 (44.41), 468 (31.67), 156 (29.14), 157 (25.20), 469 (15.01), 311 (14.91). HRMS (EI) calcd. for $C_{24}H_{23}N_2O_4S_2$: 467.1099, Found: 467.1102.

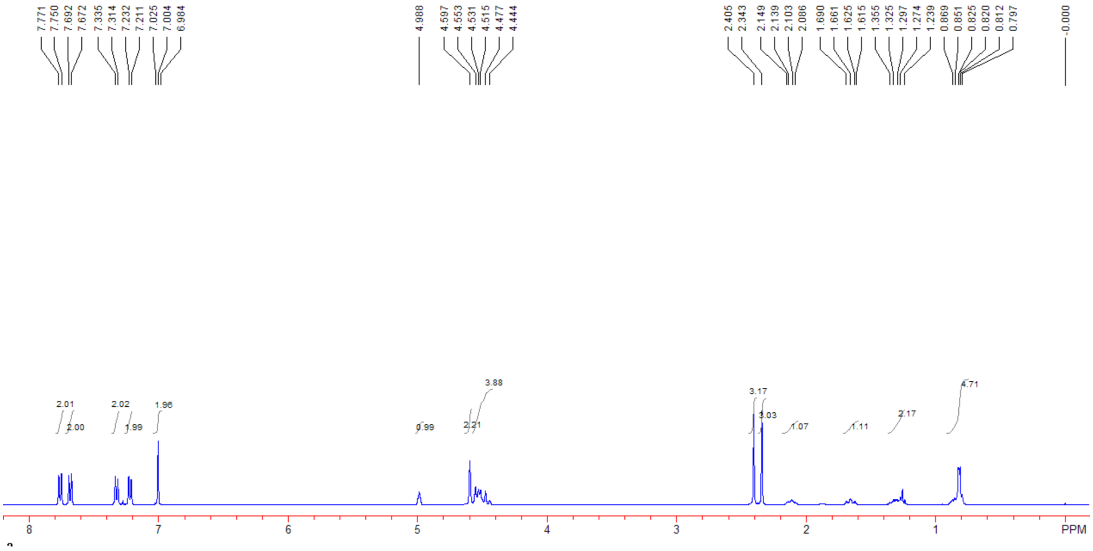

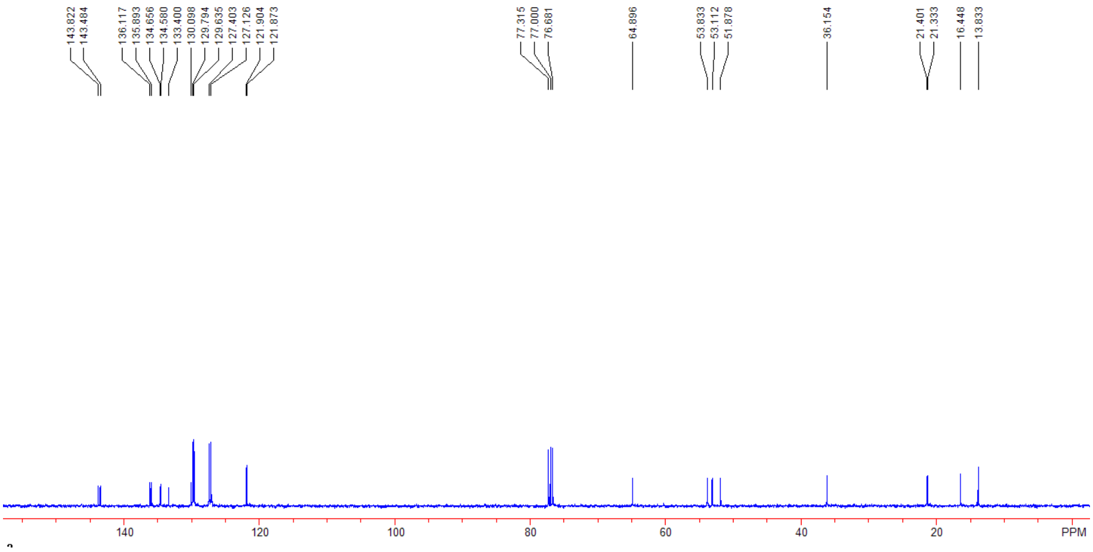

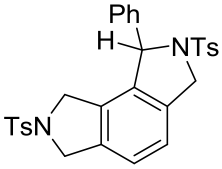

Compound 2i. 34 mg, yield: 55%, white solid, 227-229 °C. ^{1}H NMR ($CDCl_3$, 400 MHz, TMS) δ 2.34 (s, 3H, CH_3), 2.37 (s, 3H, CH_3), 3.61 (d, *J* = 14.0 Hz, 1H, CH_2), 4.24 (d, *J* = 14.0 Hz,

1H, CH_2), 4.41 (d, J = 13.6 Hz, 1H, CH_2), 4.51 (d, J = 13.6 Hz, 1H, CH_2), 4.76 (d, J = 13.6 Hz, 1H, CH_2), 4.82 (d, J = 13.6 Hz, 1H, CH_2), 5.79 (s, 1H, CH), 7.05 (d, J = 8.0 Hz, 1H, Ar), 7.07 (d, J = 8.0 Hz, 2H, Ar), 7.12 (d, J = 8.0 Hz, 1H, Ar), 7.13 (d, J = 8.0 Hz, 2H, Ar), 7.21-7.24 (m, 3H, Ar), 7.26-7.31 (m, 2H, Ar), 7.45 (d, J = 8.0 Hz, 2H, Ar), 7.53 (d, J = 8.0 Hz, 2H, Ar). ^{13}C NMR ($CDCl_3$, 100 MHz, TMS) δ 21.4, 21.5, 51.5, 53.2, 53.7, 68.3, 122.0, 122.4, 127.2, 127.3, 128.0, 128.2, 128.6, 129.4, 129.7, 131.5, 133.3, 135.0, 135.3, 135.6, 136.6, 139.4, 143.3, 143.6. IR (CH_2Cl_2) ν 2922, 2854, 1597, 1494, 1455, 1344, 1160, 1095, 815, 666 cm^{-1}. MS (ESI) m/e 545.2 (M^++1). HRMS (ESI) calcd. for $C_{30}H_{28}N_2O_4S_2$: 544.1490, Found: 544.1501.

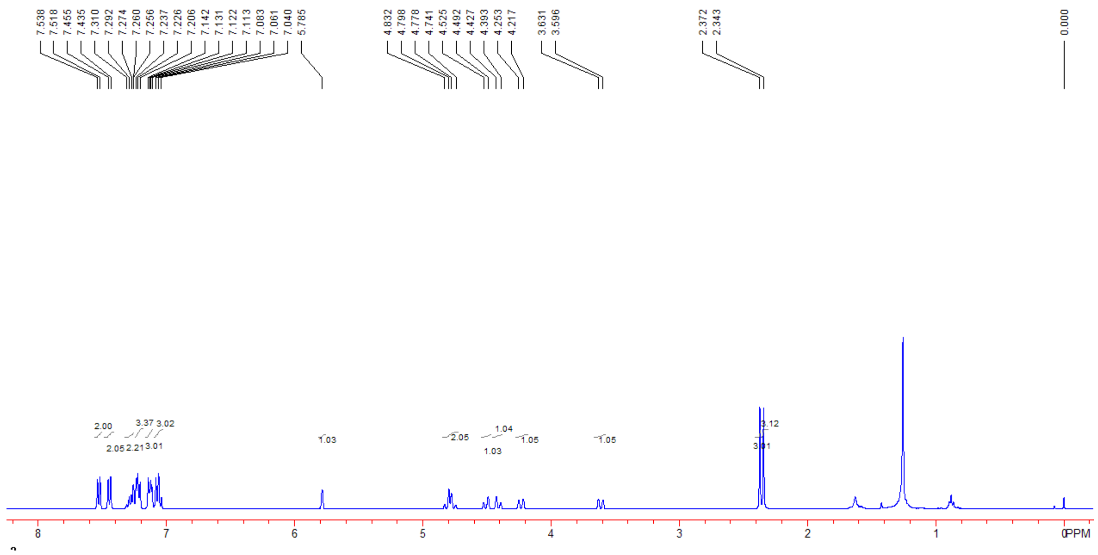

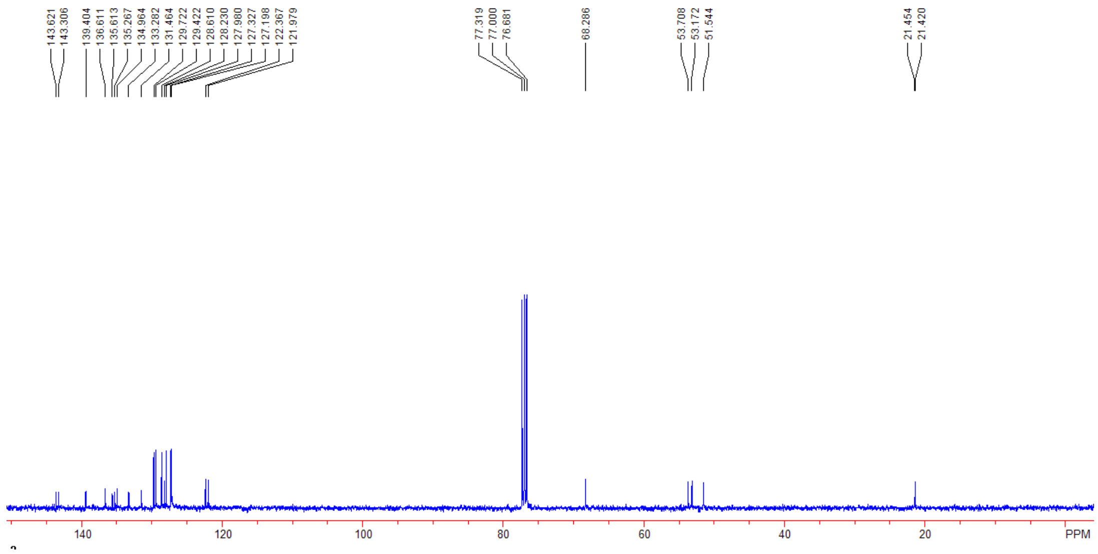

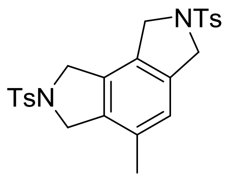

Compound 2j. 87 mg, yield: 83%, white solid, Mp: 233-235 °C. ^{1}H NMR ($CDCl_3$, 400 MHz, TMS) δ 2.15 (s, 3H, CH_3), 2.40 (s, 3H, CH_3), 2.41 (s, 3H, CH_3), 4.41 (s, 2H, CH_2), 4.46 (s, 2H, CH_2), 4.50 (s, 2H, CH_2), 4.54 (s, 2H, CH_2), 6.84 (s, 1H, Ar), 7.31 (d, *J* = 8.0 Hz, 2H, Ar), 7.32 (d, *J* = 8.0 Hz, 2H, Ar), 7.74 (d, *J* = 8.0 Hz, 2H, Ar), 7.76 (d, *J* = 8.0 Hz, 2H, Ar). ^{13}C NMR ($CDCl_3$, 100 MHz, TMS) δ 18.6, 21.5, 52.0, 52.5, 52.9, 53.3, 122.7, 127.5, 127.9, 129.85, 129.89, 130.3, 132.4, 133.47, 133.55, 135.0, 136.5, 143.80, 143.84. IR (CH_2Cl_2) ν 2927, 2853, 1596, 1447, 1339, 1307, 1158, 1096, 1067, 818, 666 cm^{-1}. MS (ESI) m/e 483.1 (M^++1). HRMS (ESI) calcd. for $C_{25}H_{26}N_2O_4S_2$: 482.1334, Found: 482.1345.

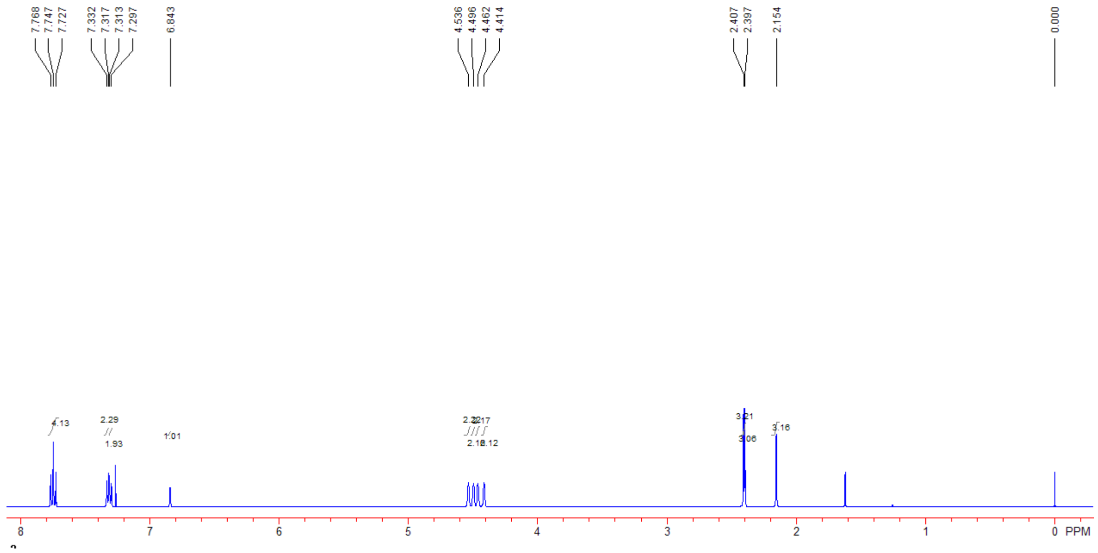

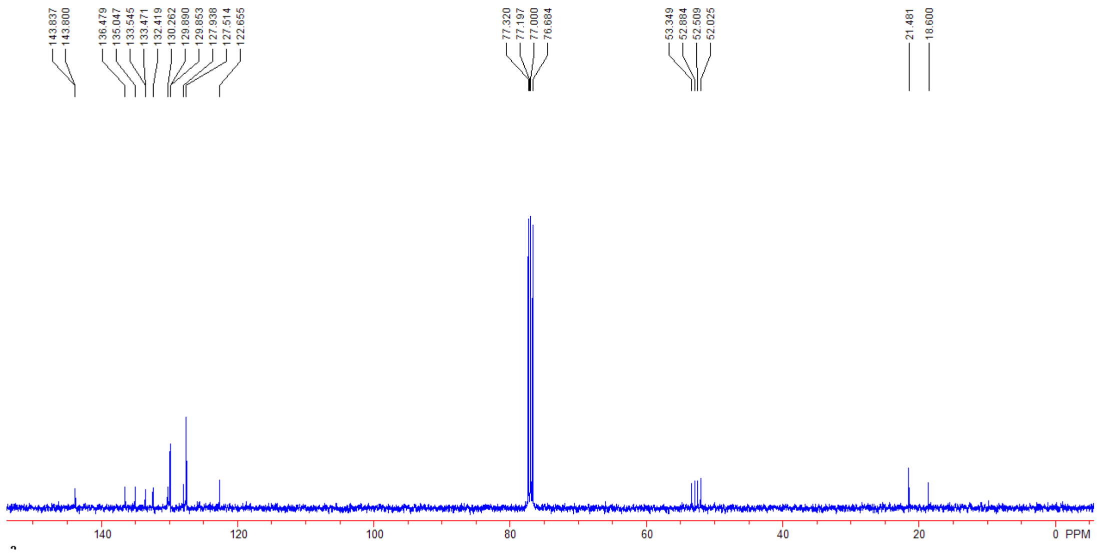

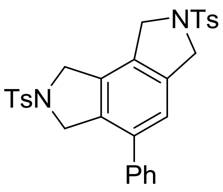

Compound 2k. 63 mg, yield: 58%, white solid, 256-258 °C. ^{1}H NMR ($CDCl_3$, 400 MHz, TMS) δ 2.40 (s, 3H, CH_3), 2.41 (s, 3H, CH_3), 4.50 (s, 4H, CH_2), 4.58 (s, 2H, CH_2), 4.62 (s, 2H, CH_2), 7.04 (s, 1H, Ar), 7.23 (d, J = 8.0 Hz, 2H, Ar), 7.30-7.42 (m, 7H, Ar), 7.72 (d, J = 8.0 Hz, 2H, Ar), 7.77 (d, J = 8.0 Hz, 2H, Ar). ^{13}C NMR ($CDCl_3$, 100 MHz, TMS) δ 21.5, 52.1, 52.3, 53.3, 53.4, 122.4, 127.5, 127.8, 127.9, 128.8, 129.6, 129.9, 131.3, 133.3, 134.1, 136.9, 137.4, 139.0, 143.87, 143.90. IR (CH_2Cl_2) ν 2956, 2923, 2853, 1596, 1465, 1345, 1307, 1163, 1097, 1070, 669 cm^{-1}. MS (ESI) m/e 545.2 (M^{+}+1). HRMS (ESI) calcd. for $C_{30}H_{28}N_2O_4S_2$: 544.1490, Found: 544.1498.

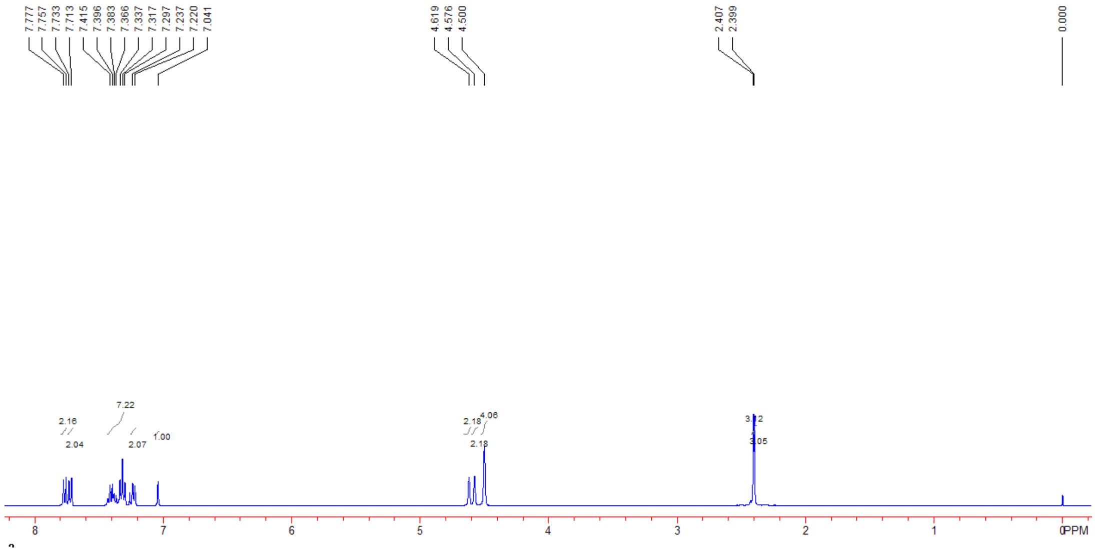

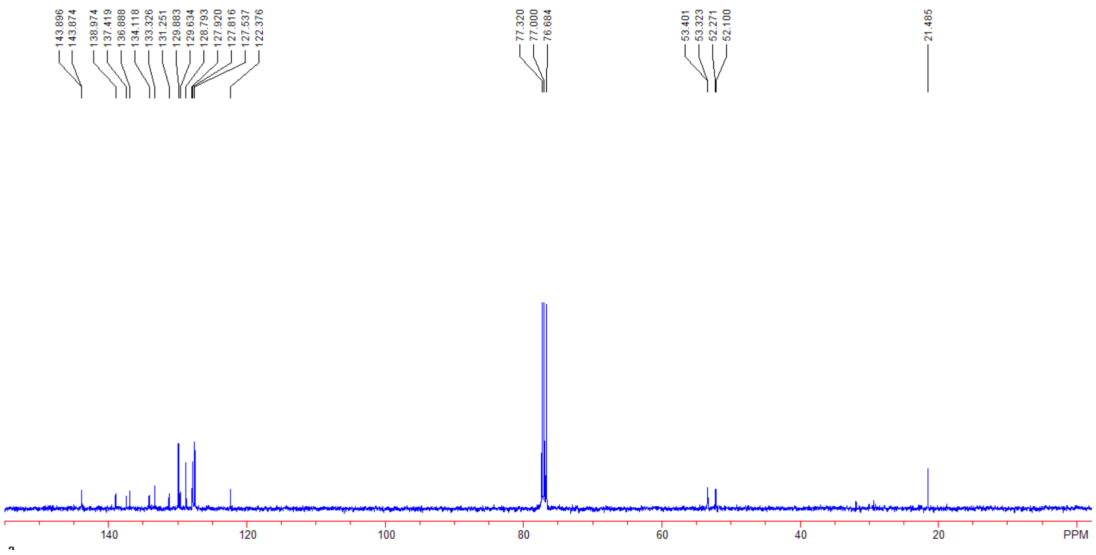

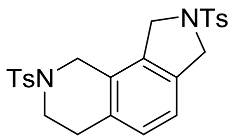

Compound 2l. 89 mg, yield: 92%, white solid, Mp: 254-256 °C. ^{1}H NMR ($(CD_3)_2SO$, 400 MHz, TMS) δ 2.34 (s, 3H, CH_3), 2.38 (s, 3H, CH_3), 2.76 (t, *J* = 5.6 Hz, 2H, CH_2), 3.19 (t, *J* = 5.6 Hz, 2H, CH_2), 4.07 (s, 2H, CH_2), 4.46 (s, 2H, CH_2), 4.52 (s, 2H, CH_2), 6.98 (d, *J* = 8.0 Hz, 1H, Ar), 7.02 (d, *J* = 8.0 Hz, 1H, Ar), 7.40 (d, *J* = 8.0 Hz, 2H, Ar), 7.41 (d, *J* = 8.0 Hz, 2H, Ar), 7.74 (d, *J* = 8.0 Hz, 2H, Ar), 7.78 (d, *J* = 8.0 Hz, 2H, Ar). ^{13}C NMR ($(CD_3)_2SO$, 100 MHz, TMS) δ 21.0, 28.0, 43.2, 44.8, 52.1, 53.3, 120.9, 126.7, 127.6, 128.4, 129.9, 130.0, 132.4, 132.9, 132.99, 133.03, 133.6, 143.66, 143.69. IR (CH_2Cl_2) ν 2925, 2850, 1597, 1491, 1454, 1339, 1163, 1097, 666 cm^{-1}. MS (ESI) m/e 500.2 (M^{+}+NH_4). HRMS (ESI) calcd. for $C_{25}H_{26}N_2O_4S_2$: 482.1334, Found: 482.1328.

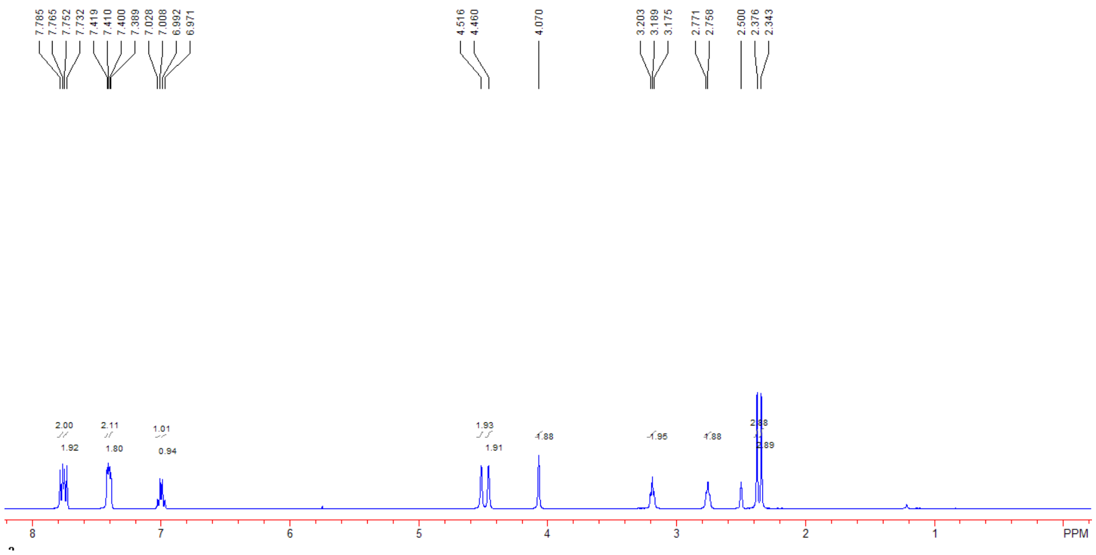

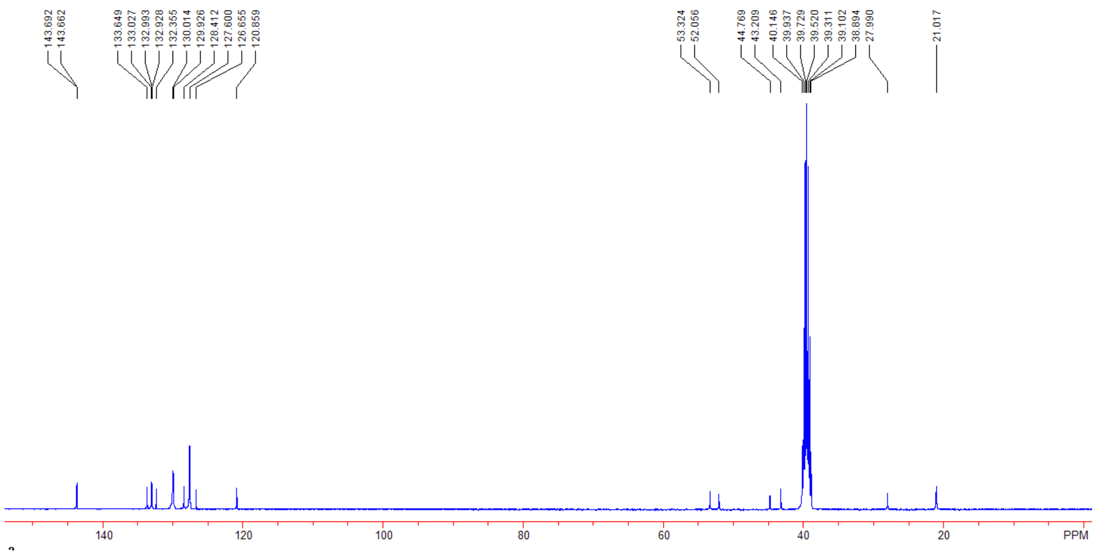

Table SI-1. Optimization of the Reaction Conditions for the Intramolecular Tandem Cross-Metathesis Reactions of Enediynes.

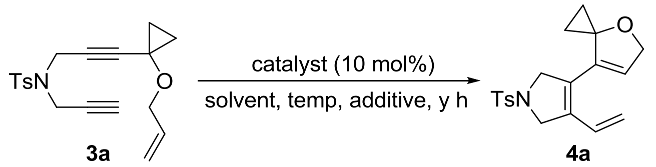

entry[a]	catalyst	solvent	additive	time (h)	temp	yield (%)[b] 4a
1	**Ru gen-1**	styrene	-	12	rt	27[c]
2	**Ru gen-1**	CH_2Cl_2	-	12	rt	15
3	**Ru gen-1**	CH_2Cl_2	-	6	rt	18
4	**Ru gen-1**	THF	-	12	rt	10
5	**Ru gen-1**	DMF	-	12	rt	-
6	**Ru gen-1**	CH_3CN	-	12	rt	-
7	**Ru gen-1**	DCE	-	12	rt	37
8	**Ru gen-1**	toluene	-	12	rt	16
9	**Ru gen-1**	1,4-dioxane	-	12	rt	complex
10	**Ru-4**	CH_2Cl_2	-	12	rt	-
11	**Ru gen-2**	CH_2Cl_2	-	12	rt	-
12	**Ru-5**	CH_2Cl_2	-	12	rt	-
13	**Ru gen-1**	CH_2Cl_2	styrene (2.0 equiv)	12	rt	10
14	***Ru gen-1***	***DCE***	***-***	***12***	***70 °C***	***63 (52)[c]***
15	**Ru gen-1**	DCE	-	12	65 °C	53 (45)[c]
16	**Ru gen-1**	DCE	-	12	reflux	13
17	**Ru gen-1**	DCE	-	24	70 °C	complex
18	**Ru gen-1**	toluene	-	12	70 °C	21
19	**Ru gen-1**	DCE	styrene (10 equiv)	12	70 °C	54
20	**Ru gen-1**	DCE	Ti(O-*i*Pr)$_4$ (0.3 equiv)	12	70 °C	23
21	**Ru gen-1**	toluene	-	12	70 °C	21
22	**Ru gen-1**	styrene	-	12	70 °C	complex
23	**Ru gen-1**	DCE	hydroquinone (0.2 equiv)	12	70 °C	12
24	**Ru gen-1**	DCE	hydroquinone (1.0 equiv)	12	70 °C	28
25	**Ru gen-1**	DCE	BHT (1.0 equiv)	12	70 °C	16
26	**Ru-3**	DCE	-	12	70 °C	complex
27	**Ru gen-1**	DCE	-	12	reflux	13
28	**Ru gen-1**	DCE	-	12	70 °C	38[d]

[a] Enyne substrate **3a** (0.1 mmol), catalyst (10 mol%), additive and the solvent (1.0 mL) were added into a reaction tube under argon. Then, the reactions were carried out at certain temperature within y h under argon. [b] The yield was determined by ^1H NMR (using 1,3,5-trimethoxybenzene as an internal standard). [c] Isolated yields. [d] The reaction was carried out in 5.0 mL DCE.

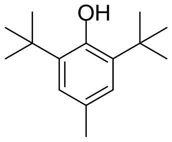

2,6-di-*tert*-butyl-4-methylphenol (BHT)

Representative procedure for the ruthenium-catalyzed intramolecular cross-metathesis of diynes:

To a flame dried Schlenk tube was added substrate **3** (0.2 mmol), Grubbs-I catalyst (10 mol%) and the solvent DCE (2.0 mL). The resulting solution was allowed to heat to 70 °C and stir at the specific temperature for 12 h. Then, the reaction mixture was evaporated to dryness and

the residue was purified by flash silica gel column chromatography (PE:EA = 10:1~4:1).

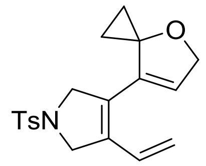

Compound 4a. 54 mg, yield: 68%, colorless oil. ^{1}H NMR ($CDCl_3$, 400 MHz, TMS) δ 0.51 (dd, J_1 = 8.0 Hz, J_2 = 6.4 Hz, 2H, CH_2), 0.93 (dd, J_1 = 8.0 Hz, J_2 = 6.4 Hz, 2H, CH_2), 2.44 (s, 3H, CH_3), 4.06 (t, J = 4.0 Hz, 2H, CH_2), 4.26 (t, J = 4.0 Hz, 2H, CH_2), 4.75 (d, J = 2.0 Hz, 2H, CH_2), 5.08 (d, J = 18.0 Hz, 1H, =CH_2), 5.21 (d, J = 10.8 Hz, 1H, =CH_2), 5.76 (t, J = 2.0 Hz, 1H, =CH), 6.48 (dd, J_1 = 18.0 Hz, J_2 = 10.8 Hz, 1H, =CH), 7.34 (d, J = 8.0 Hz, 2H, Ar), 7.72 (d, J = 8.0 Hz, 2H, Ar). ^{13}C NMR ($CDCl_3$, 100 MHz, TMS) δ 10.3, 21.5, 54.4, 57.4, 71.9, 73.4, 117.6, 126.87, 126.94, 127.4, 128.4, 129.9, 133.8, 134.9, 143.8. IR (CH_2Cl_2) ν 2927, 2858, 1597, 1454, 1345, 1163, 1095, 817 cm^{-1}. MS (ESI) m/e 344.1 (M^++1). HRMS (ESI) calcd. for $C_{19}H_{21}NO_3S$: 343.1242, Found: 343.1248.

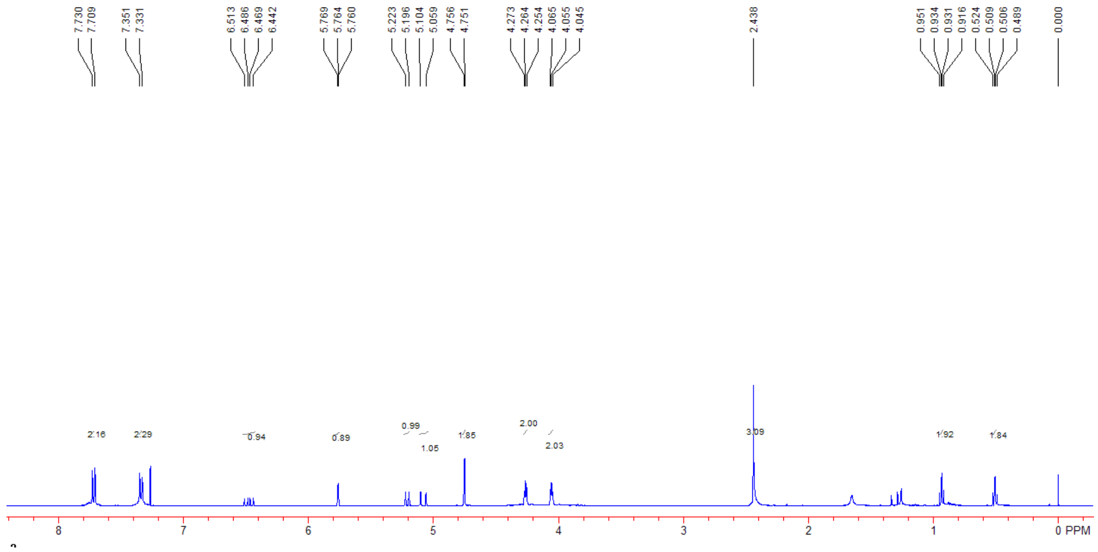

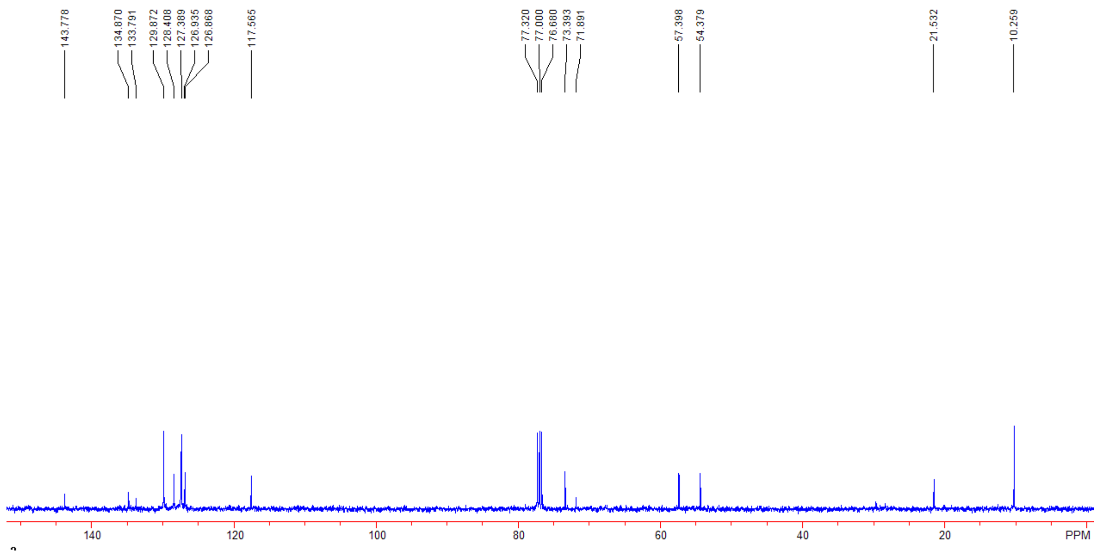

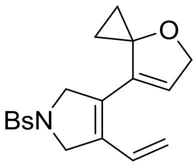

Compound 4b. 21 mg, yield: 25%, light yellow oil. ^{1}H NMR ($CDCl_3$, 300 MHz, TMS) δ 0.53 (t, J = 7.2 Hz, 2H, CH_2), 0.96 (t, J = 7.2 Hz, 2H, CH_2), 4.06 (t, J = 4.2 Hz, 2H, CH_2), 4.26 (t, J = 3.9 Hz, 2H, CH_2), 4.77 (s, 2H, CH_2), 5.09 (d, J = 17.7 Hz, 1H, $=CH_2$), 5.23 (d, J = 11.1 Hz, 1H, $=CH_2$), 5.79 (s, 1H, =CH), 6.49 (dd, J_1 = 17.7 Hz, J_2 = 11.1 Hz, 1H, =CH), 7.70 (s, 4H, Ar). ^{13}C NMR ($CDCl_3$, 100 MHz, TMS) δ 10.3, 54.4, 57.4, 71.9, 73.4, 117.8, 126.8, 127.1, 128.0, 128.3, 128.8, 132.6, 134.6, 134.8, 135.9. IR (CH_2Cl_2) ν 3288, 2924, 2854, 1575, 1471, 1390, 1355, 1166, 1093, 1069, 1010, 765 cm^{-1}. MS (ESI) m/e 425.1 (M^++NH_4). HRMS (ESI) calcd. for $C_{18}H_{18}BrNO_3S$: 407.0191, Found: 407.0193.

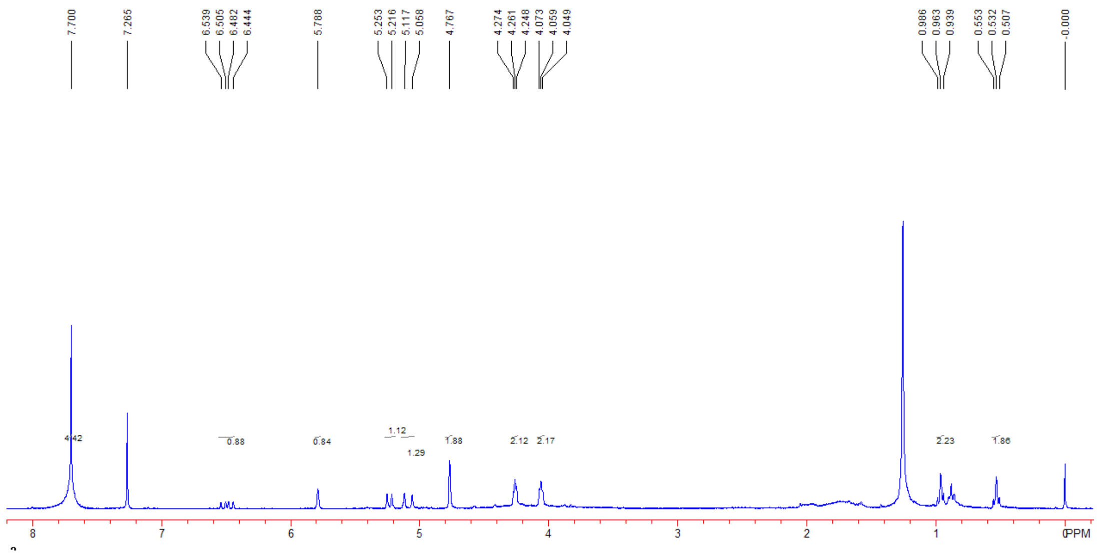

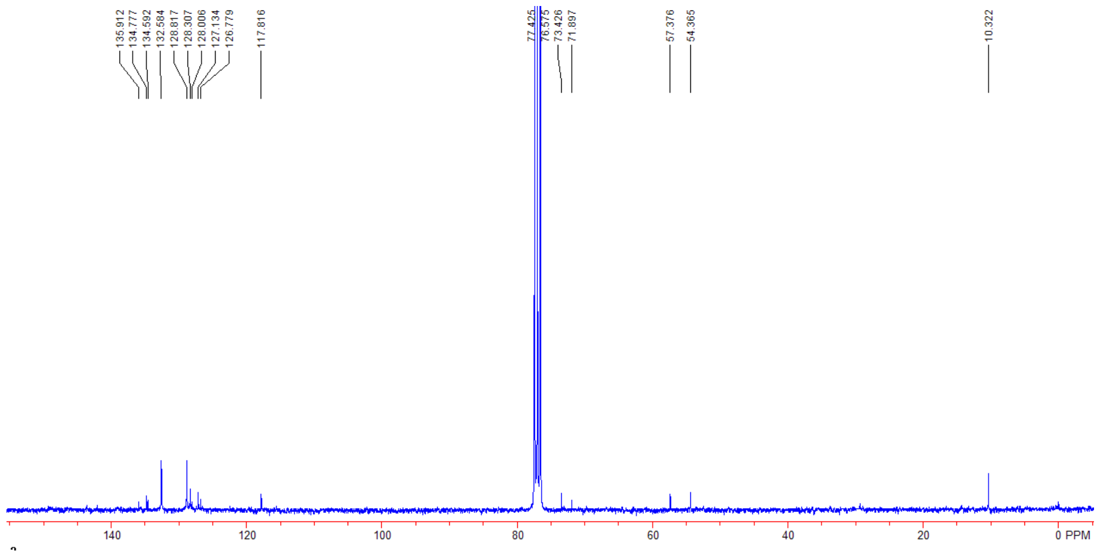

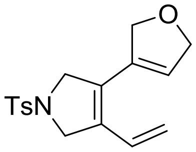

Compound 4c. 43 mg, yield: 68%, white solid, Mp: 139-141 ^{o}C. ^{1}H NMR ($CDCl_3$, 400 MHz, TMS) δ 2.43 (s, 3H, CH_3), 4.32 (s, 4H, CH_2), 4.68 (t, J = 1.6 Hz, 2H, CH_2), 4.85 (td, J_1 = 4.4 Hz, J_2 = 2.0 Hz, 2H, CH_2), 5.11 (d, J = 16.4 Hz, 1H, =CH_2), 5.25 (d, J = 10.4 Hz, 1H, =CH_2), 5.81 (t, J = 1.6 Hz, 1H, =CH), 6.37 (dd, J_1 = 16.4 Hz, J_2 = 10.4 Hz, 1H, =CH), 7.35 (d, J = 8.0 Hz, 2H, Ar), 7.76 (d, J = 8.0 Hz, 2H, Ar). ^{13}C NMR ($CDCl_3$, 100 MHz, TMS) δ 21.5, 55.2, 56.6, 74.9, 75.1, 118.5, 126.1, 126.6. 127.5, 128.1, 129.9, 131.8, 132.7, 133.5, 143.7. IR (CH_2Cl_2) ν 2959, 2924, 2855, 1597, 1494, 1456, 1341, 1161, 1093, 1069, 814, 666 cm^{-1}. MS

(ESI) m/e 318.1 (M^++1). HRMS (ESI) calcd. for $C_{17}H_{19}NO_3S$: 317.1086, Found: 317.1070.

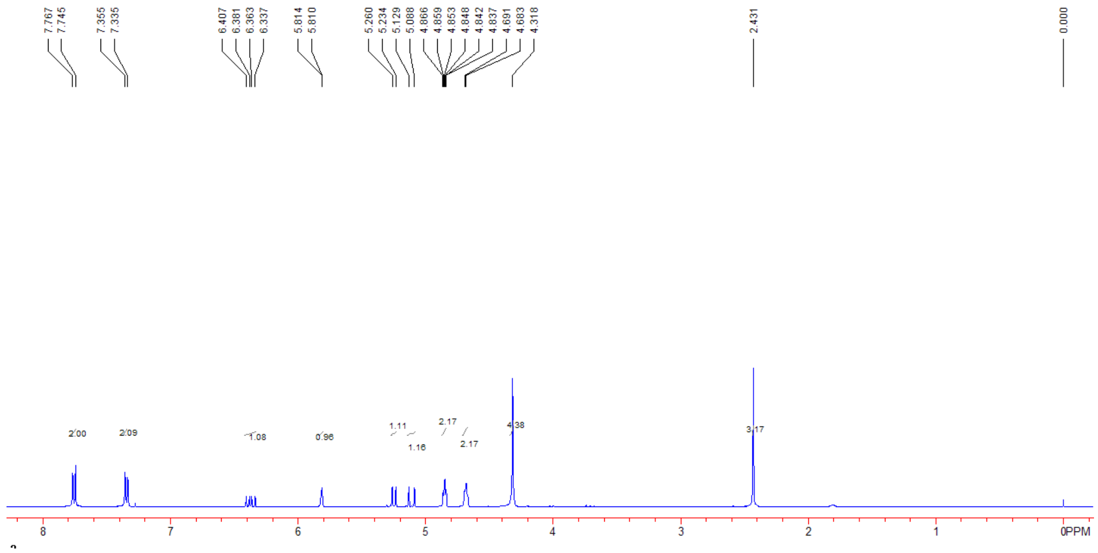

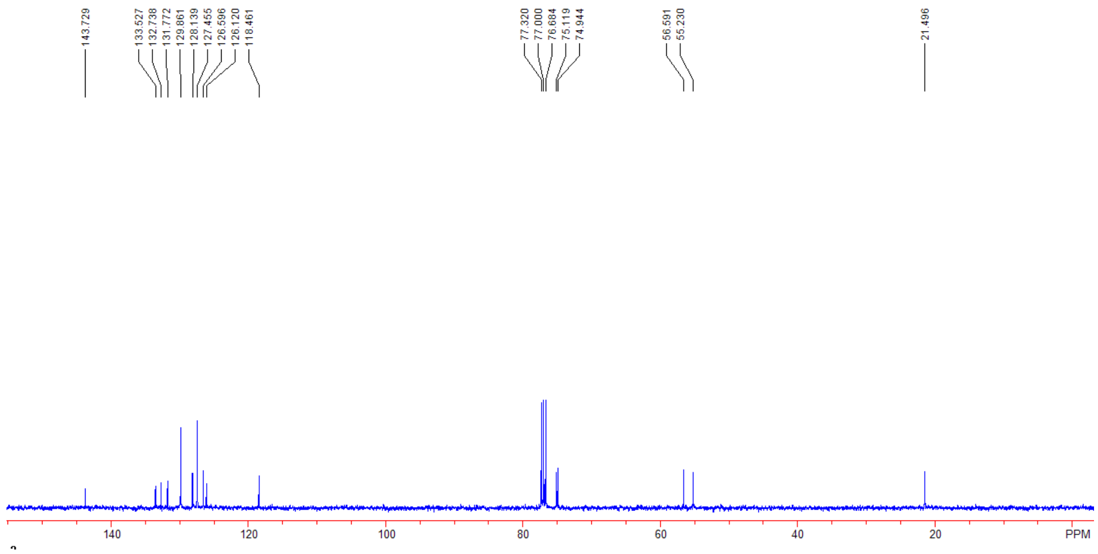

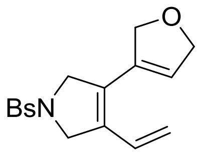

Compound 4d. 52 mg, yield: 68%, white solid, Mp: 110-112 °C. ^{1}H NMR ($CDCl_3$, 400 MHz, TMS) δ 4.32 (s, 4H, CH_2), 4.69 (s, 2H, CH_2), 4.86 (td, J_1 = 4.0 Hz, J_2 = 1.6 Hz, 2H, CH_2), 5.11 (d, J = 17.6 Hz, 1H, =CH_2), 5.27 (d, J = 10.4 Hz, 1H, =CH_2), 5.82 (s, 1H, =CH), 6.38 (dd, J_1 = 17.6 Hz, J_2 = 10.4 Hz, 1H, =CH), 7.69 (d, J = 8.8 Hz, 2H, Ar), 7.74 (d, J = 8.8 Hz, 2H, Ar). ^{13}C NMR ($CDCl_3$, 100 MHz, TMS) δ 55.3, 56.6, 75.0, 75.1, 118.7, 126.0, 126.8, 127.9, 128.0, 128.7, 131.6, 132.5, 132.6, 135.7. IR (CH_2Cl_2) ν 2959, 2923, 2851, 1574, 1471, 1388,

1346, 1167, 1092, 1068, 1008, 905, 739 cm^{-1}. MS (ESI) m/e 382.0 (M^{+}+1). HRMS (ESI) calcd. for $C_{16}H_{16}BrNO_3S$: 381.0034, Found: 381.0016.

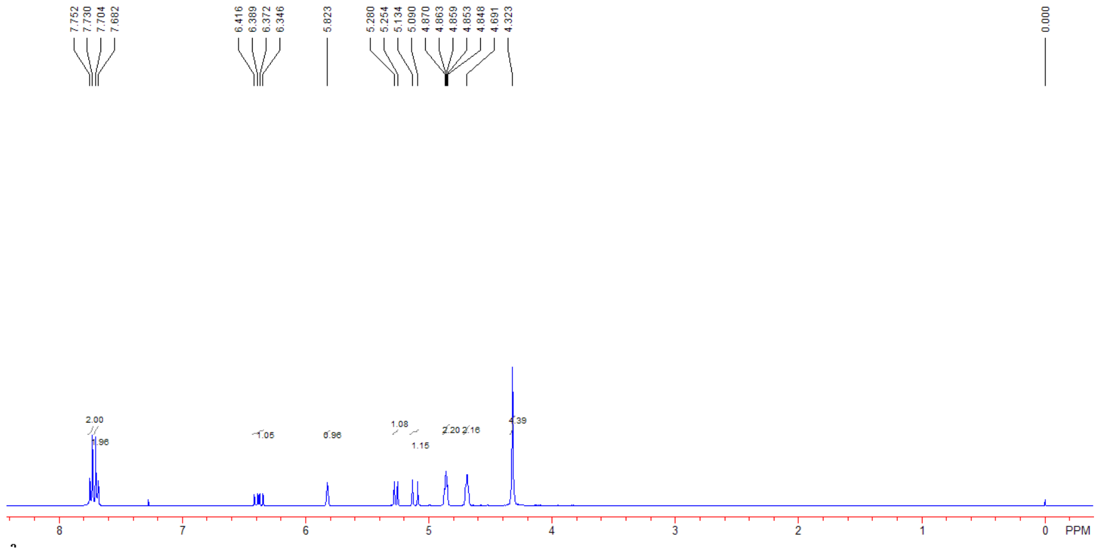

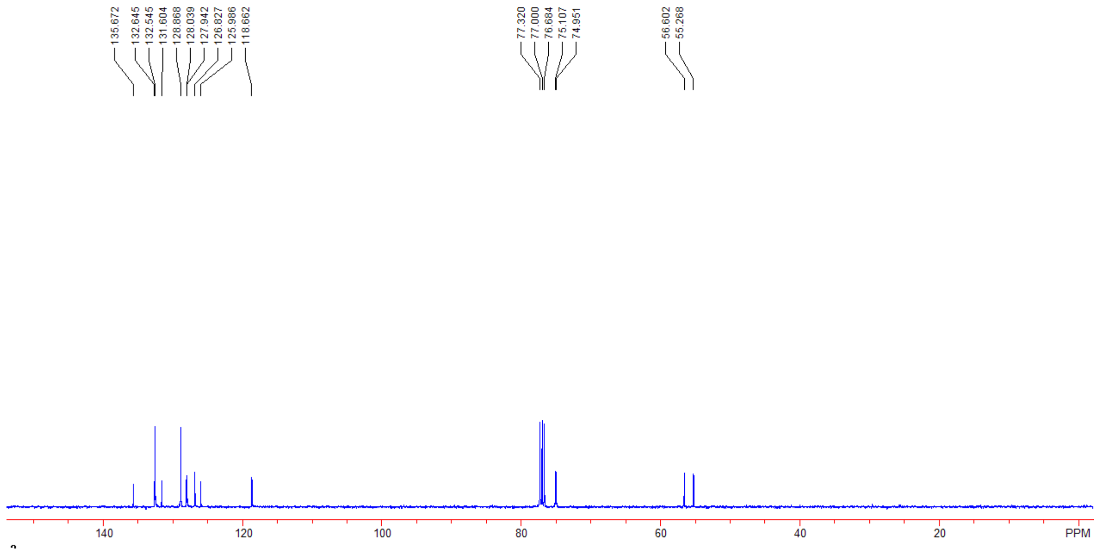

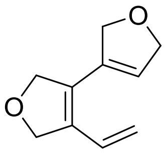

Compound 4e. 43 mg, yield: 65%, light yellow oil. ^{1}H NMR ($CDCl_3$, 400 MHz, TMS) δ 4.72 (t, J = 4.0 Hz, 2H, CH_2), 4.87-4.88 (m, 4H, CH_2), 4.96 (td, J_1 = 4.8 Hz, J_2 = 2.0 Hz, 2H, CH_2), 5.06 (d, J = 17.2 Hz, 1H, =CH_2), 5.25 (d, J = 10.8 Hz, 1H, =CH_2), 5.76 (t, J = 2.0 Hz, 1H, =CH), 6.50 (dd, J_1 = 17.2 Hz, J_2 = 10.8 Hz, 1H, =CH). ^{13}C NMR ($CDCl_3$, 100 MHz, TMS) δ 75.1, 75.2, 76.5, 77.5, 117.8, 125.3, 127.5, 127.7, 132.1, 133.2. IR (CH_2Cl_2) ν 2924, 2852,

1750, 1465, 1357, 1162, 1090, 1007, 904, 746 cm^{-1}. MS (%) m/e 164 (M^+, 25.38), 149 (23.70), 135 (29.60), 121 (19,09), 117 (14.26), 105 (31.29), 91 (93.58), 77 (73.58). HRMS (EI) calcd. for $C_{10}H_{12}O_2$: 164.0837, Found: 164.0833.

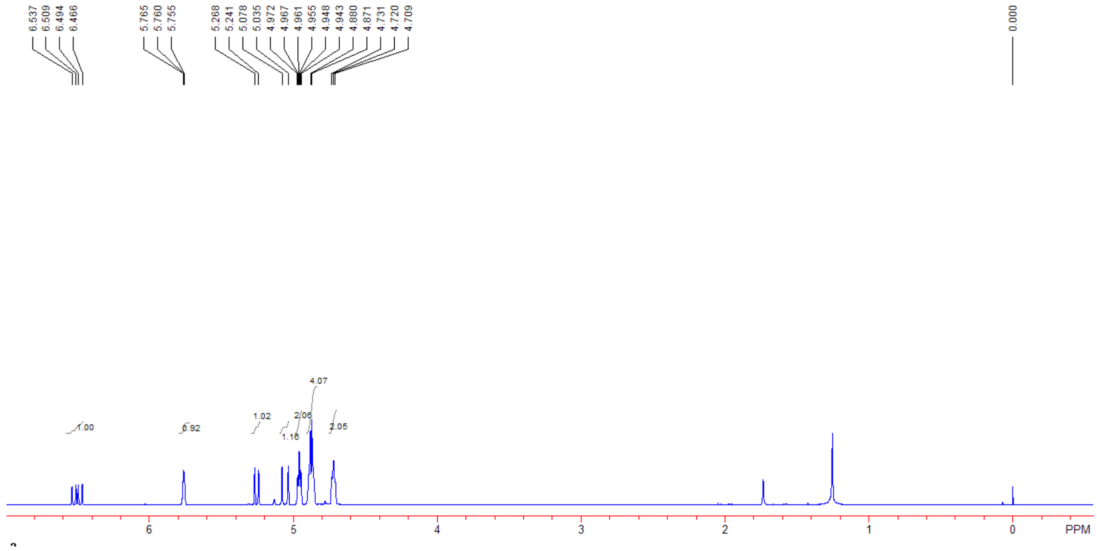

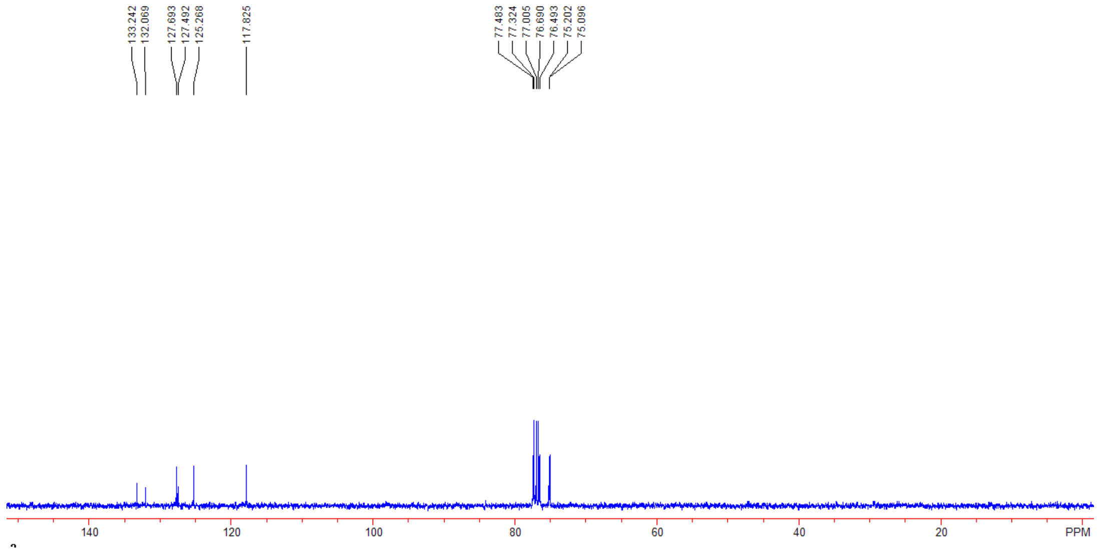

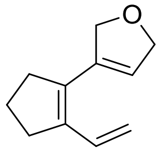

Compound 4f. 44 mg, yield: 67%, light yellow oil. ^1H NMR ($CDCl_3$, 400 MHz, TMS) δ 1.85-1.93 (m, 2H, CH_2), 2.59-2.66 (m, 4H, CH_2), 4.70 (s, 2H, CH_2), 4.93 (td, J_1 = 4.8 Hz, J_2 = 2.0 Hz, 2H, CH_2), 5.15 (d, J = 16.8 Hz, 1H, =CH_2), 5.18 (d, J = 10.8 Hz, 1H, =CH_2), 5.81 (s, 1H, =CH), 6.61 (dd, J_1 = 16.8 Hz, J_2 = 10.8 Hz, 1H, =CH). ^{13}C NMR ($CDCl_3$, 100 MHz, TMS)

δ 21.4, 33.6, 36.3, 74.8, 75.6, 116.0, 124.4, 131.5, 132.4, 136.3, 138.2. IR (CH_2Cl_2) ν 2953, 2925, 2854, 1736, 1717, 1457, 1260, 1080, 1017, 736 cm^{-1}. MS (%) m/e 162 (M^+, 7.69), 160 (22.42), 147 (8.14), 131 (48.53), 117 (42.04), 105 (21.37), 91 (58.22), 49 (100.00). HRMS (EI) calcd. for $C_{11}H_{14}O$: 162.1045, Found: 162.1043.

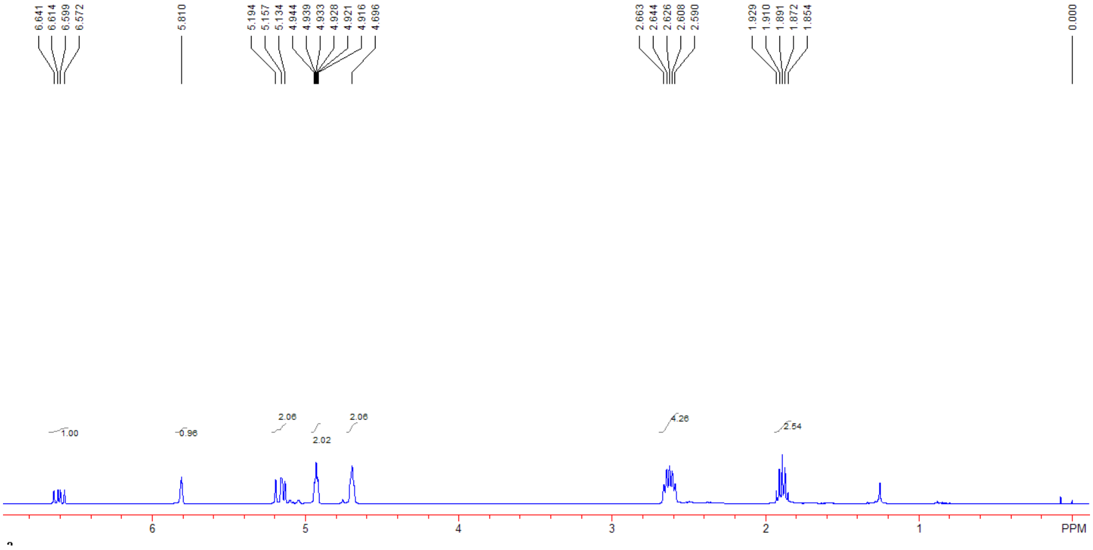

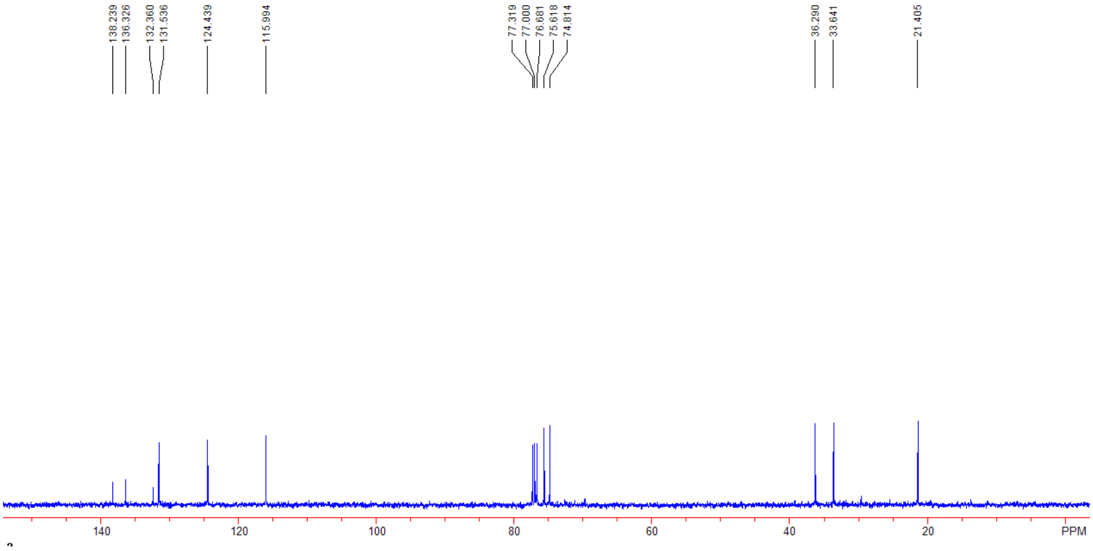

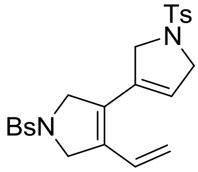

Compound 4g. 65 mg, yield: 61%, white solid, Mp: 166-168 °C. ^{1}H NMR ($CDCl_3$, 400 MHz, TMS) δ 2.43 (s, 3H, CH_3), 4.15 (s, 2H, CH_2), 4.21 (s, 2H, CH_2), 4.28 (s, 2H, CH_2), 4.35 (s, 2H, CH_2), 5.13 (d, J = 17.2 Hz, 1H, =CH_2), 5.31 (d, J = 10.4 Hz, 1H, =CH_2), 5.54 (s, 1H, =CH),

6.46 (dd, J_1 = 17.2 Hz, J_2 = 10.4 Hz, 1H, =CH), 7.33 (d, J = 8.0 Hz, 2H, Ar), 7.67 (d, J = 8.8 Hz, 2H, Ar), 7.70 (d, J = 8.8 Hz, 2H, Ar), 7.72 (d, J = 8.0 Hz, 2H, Ar). ^{13}C NMR ($CDCl_3$, 100 MHz, TMS) δ 21.5, 54.0, 54.1, 55.4, 56.4, 119.5, 125.2, 125.9, 127.4, 127.7, 128.1, 128.9, 129.9, 131.6, 132.5, 132.6, 133.8, 135.7, 143.8. IR (CH_2Cl_2) ν 2960, 2923, 2854, 1597, 1574, 1470, 1388, 1347, 1159, 1101, 667 cm^{-1}. MS (ESI) m/e 535.0 (M^++1). HRMS (ESI) calcd. for $C_{23}H_{23}BrN_2O_4S_2$: 534.0283, Found: 534.0296.

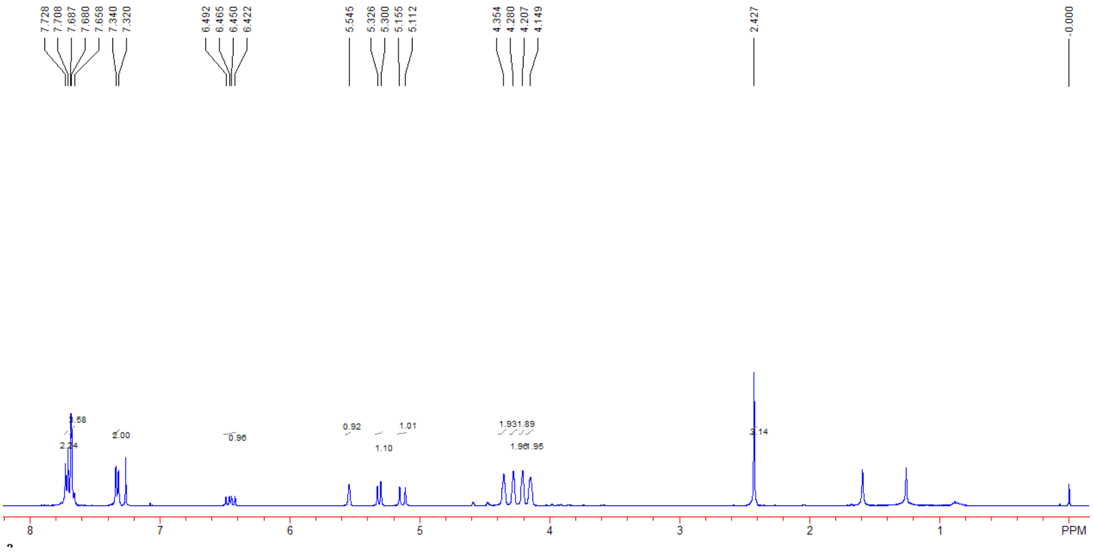

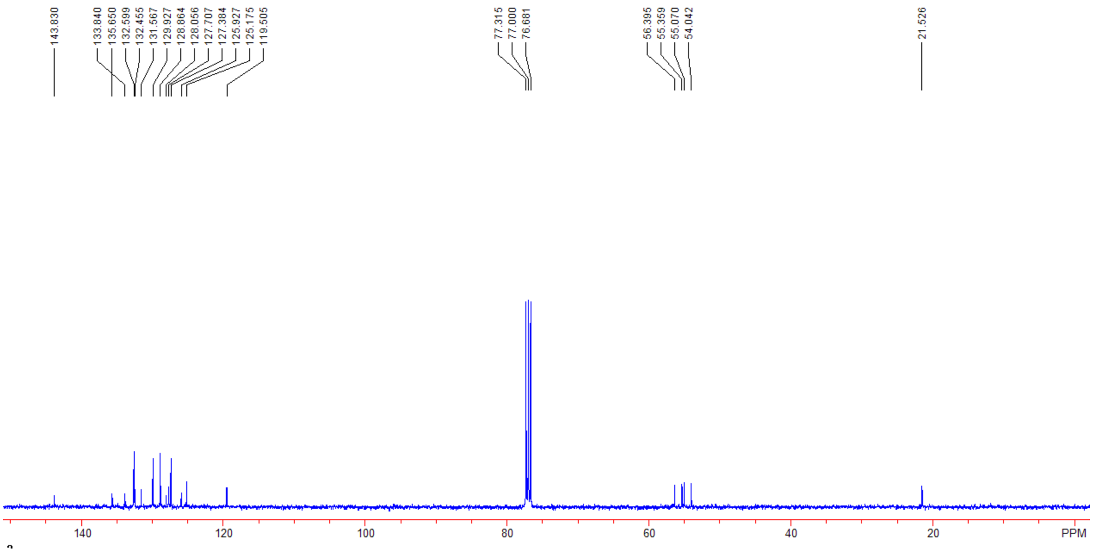

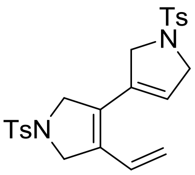

Compound 4h. 52 mg, yield: 55%, white solid, Mp: 145-147 °C. ^{1}H NMR ($CDCl_3$, 400 MHz,

TMS) δ 2.42 (s, 6H, CH_3), 4.14 (s, 2H, CH_2), 4.20 (s, 2H, CH_2), 4.27 (s, 2H, CH_2), 4.34 (s, 2H, CH_2), 5.13 (d, J = 16.8 Hz, 1H, =CH_2), 5.29 (d, J = 10.4 Hz, 1H, =CH_2), 5.54 (s, 1H, =CH), 6.45 (dd, J_1 = 16.8 Hz, J_2 = 10.4 Hz, 1H, =CH), 7.33 (d, J = 8.0 Hz, 2H, Ar), 7.71 (d, J = 8.0 Hz, 2H, Ar). ^{13}C NMR ($CDCl_3$, 100 MHz, TMS) δ 21.5, 21.9, 54.0, 55.0, 55.3, 56.3, 119.3, 124.9, 126.0, 127.3, 127.4, 127.7, 129.9, 131.5, 132.5, 133.4, 133.7, 143.8. IR (CH_2Cl_2) ν 2925, 2856, 1727, 1597, 1455, 1343, 1231, 1161, 1102, 815, 667 cm^{-1}. MS (ESI) m/e 471.1 (M^++1). HRMS (ESI) calcd. for $C_{24}H_{26}N_2O_4S_2$: 470.1334, Found: 470.1325.

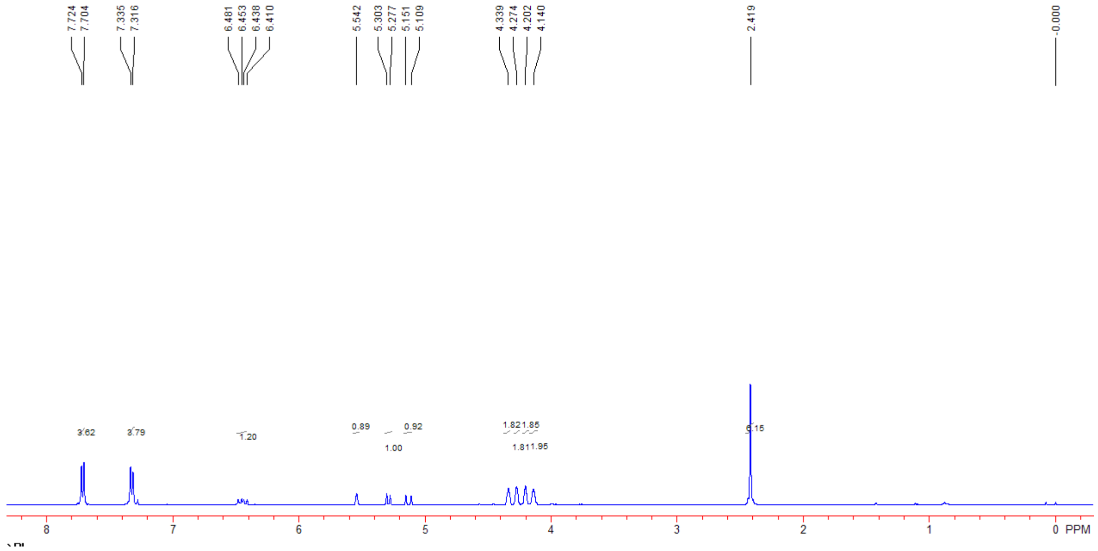

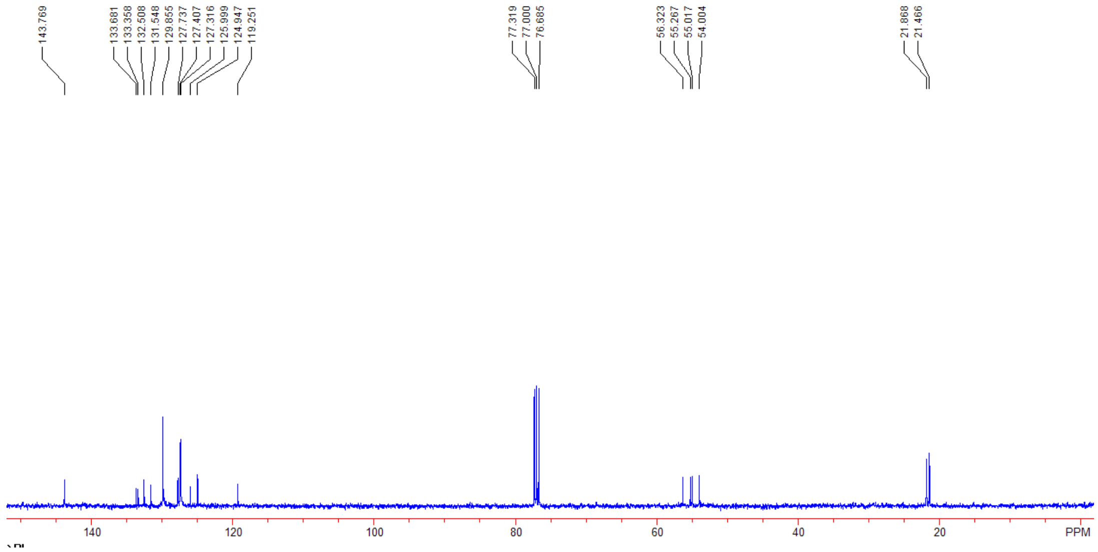

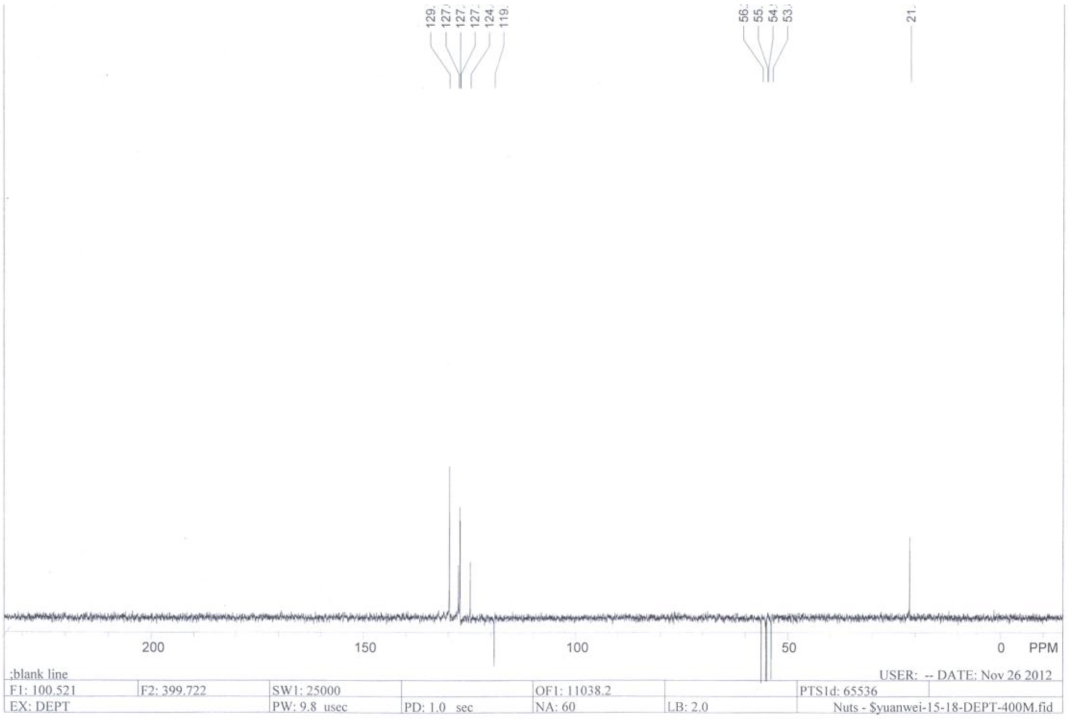
129.
127.
127.
127.
124.
119.
56.
55.
54.
53.
21.
200
150
100
50
0 PPM
;blank line
USER: -- DATE: Nov 26 2012
F1: 100.521
F2: 399.722
SW1: 25000
OF1: 11038.2
PTS1d: 65536
EX: DEPT
PW: 9.8 usec
PD: 1.0 sec
NA: 60
LB: 2.0
Nuts - $yuanwei-15-18-DEPT-400M.fid

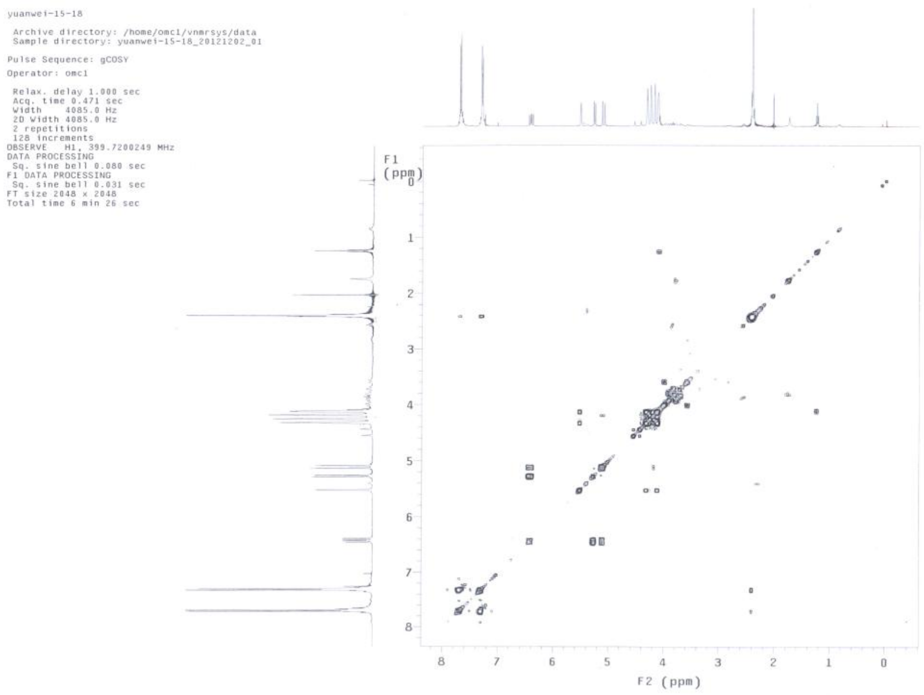
yuanwei-15-18
Archive directory: /home/omc1/vnmrsys/data
Sample directory: yuanwei-15-18_20121202_01
Pulse Sequence: gCOSY
Operator: omc1
Relax. delay 1.000 sec
Acq. time 0.471 sec
Width 4085.0 Hz
2D Width 4085.0 Hz
2 repetitions
128 increments
OBSERVE H1, 399.7200249 MHz
DATA PROCESSING
Sq. sine bell 0.080 sec
F1 DATA PROCESSING
Sq. sine bell 0.031 sec
FT size 2048 x 2048
Total time 6 min 26 sec
F1 (ppm)
F2 (ppm)

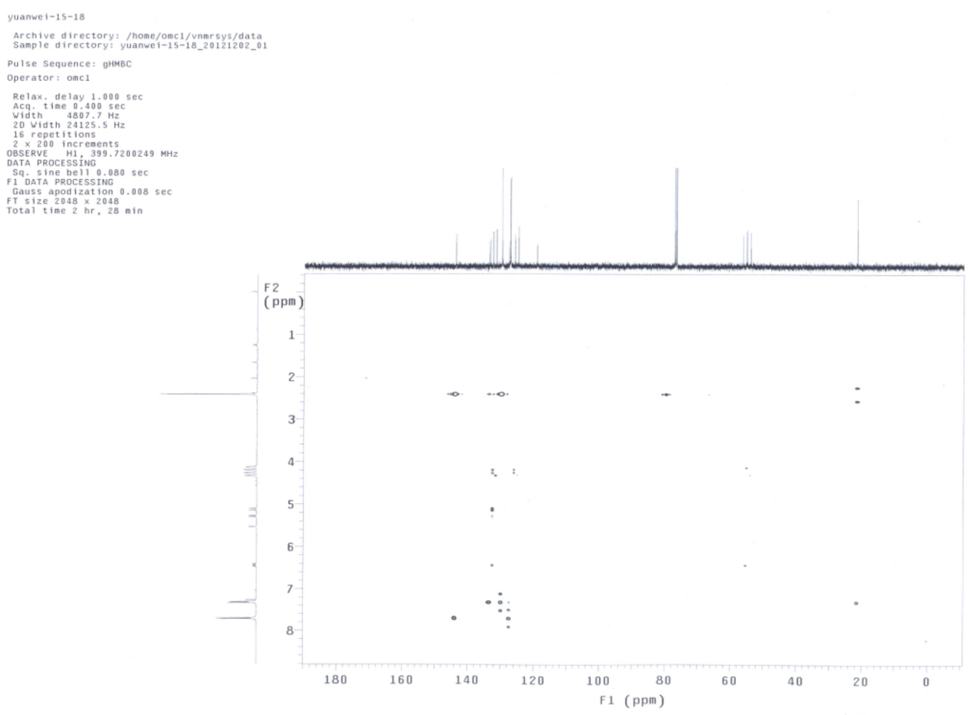
yuanwei-15-18
Archive directory: /home/omc1/vnmrsys/data
Sample directory: yuanwei-15-18_20121202_01
Pulse Sequence: gHMBC
Operator: omc1
Relax. delay 1.000 sec
Acq. time 0.400 sec
Width 4807.7 Hz
2D Width 24125.5 Hz
16 repetitions
2 x 200 increments
OBSERVE H1, 399.7200249 MHz
DATA PROCESSING
Sq. sine bell 0.080 sec
F1 DATA PROCESSING
Gauss apodization 0.008 sec
FT size 2048 x 2048
Total time 2 hr, 28 min
F2 (ppm)
1
2
3
4
5
6
7
8
180
160
140
120
100
80
60
40
20
0
F1 (ppm)

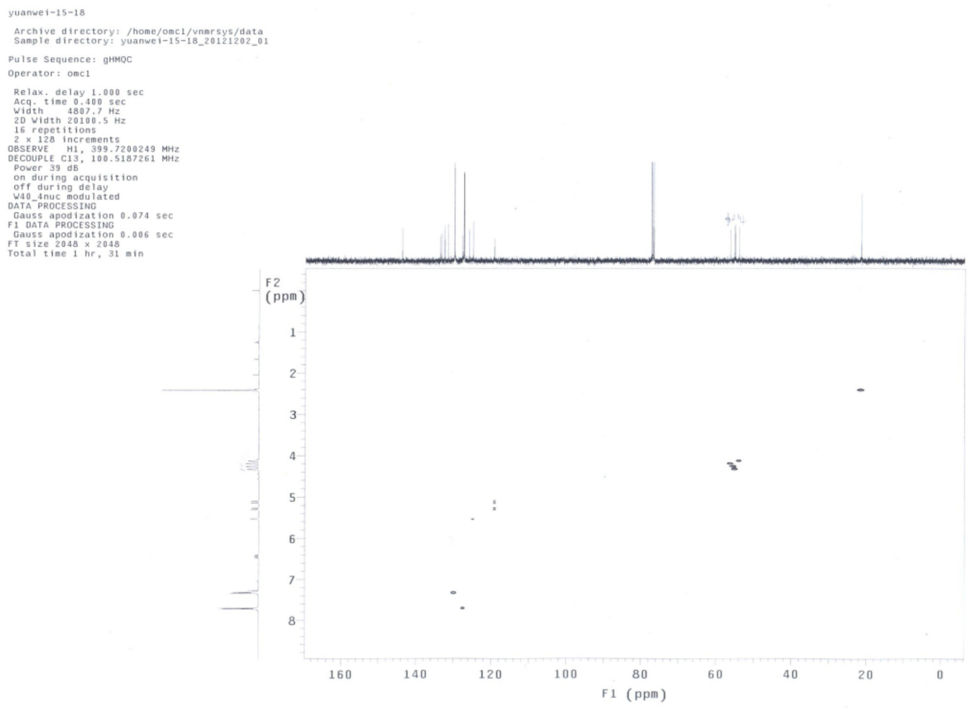
yuanwei-15-18
Archive directory: /home/omc1/vnmrsys/data
Sample directory: yuanwei-15-18_20121202_01
Pulse Sequence: gHMQC
Operator: omc1
Relax. delay 1.000 sec
Acq. time 0.400 sec
Width 4807.7 Hz
2D Width 20100.5 Hz
16 repetitions
2 x 128 increments
OBSERVE H1, 399.7200249 MHz
DECOUPLE C13, 100.5187261 MHz
Power 39 dB
on during acquisition
off during delay
W40_4nuc modulated
DATA PROCESSING
Gauss apodization 0.074 sec
F1 DATA PROCESSING
Gauss apodization 0.006 sec
FT size 2048 x 2048
Total time 1 hr, 31 min
F2 (ppm)
1
2
3
4
5
6
7
8
160
140
120
100
80
60
40
20
0
F1 (ppm)

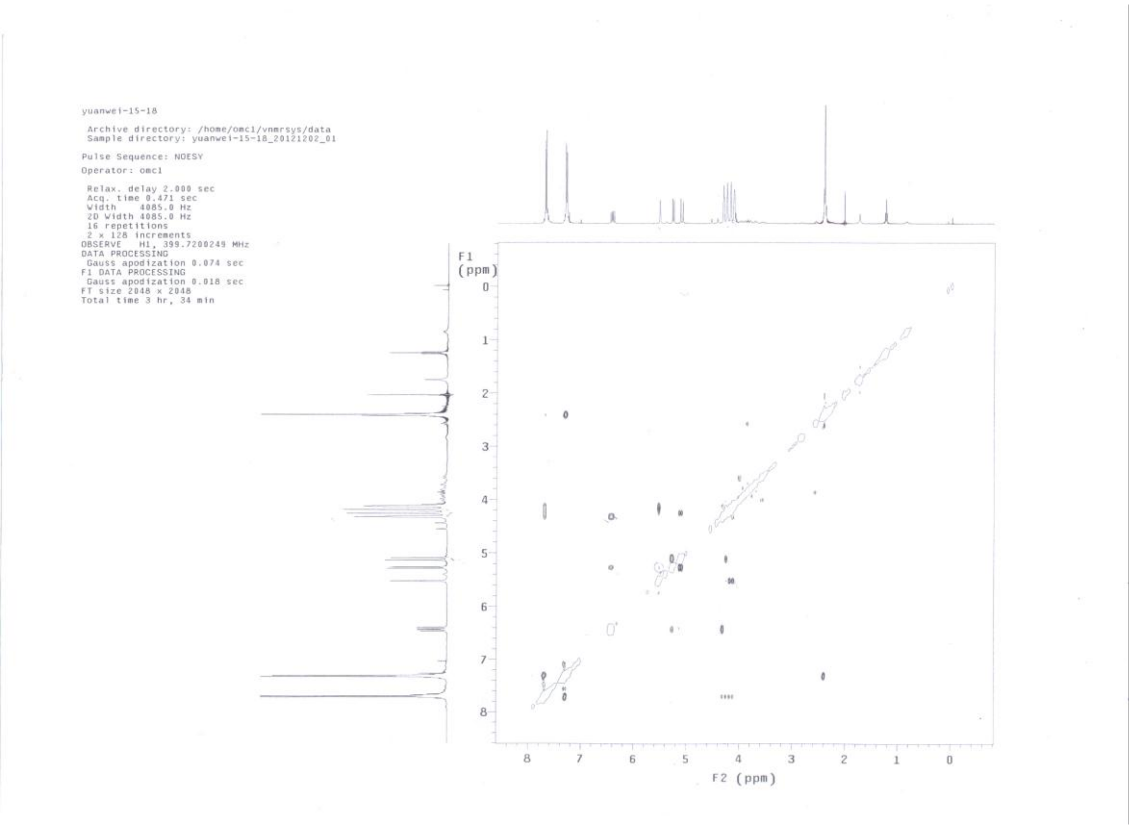

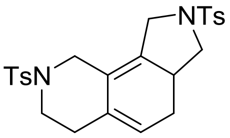

Compound 4k. 65 mg, yield: 67%, white solid, Mp: 218-220 °C. ^{1}H NMR ($CDCl_3$, 400 MHz, TMS) δ 1.64 (t, *J* = 17.6 Hz, 1H, CH_2), 2.11-2.19 (m, 2H, CH_2), 2.31-2.35 (m, 1H, CH), 2.42 (s, 3H, CH_3), 2.44 (s, 3H, CH_3), 2.58-2.68 (m, 2H, CH_2), 2.81 (brs, 1H, CH_2), 3.28 (d, *J* = 14.8 Hz, 1H, CH_2), 3.54-3.59 (m, 1H, CH_2), 3.64 (d, *J* = 15.2 Hz, 1H, CH_2), 3.80 (t, *J* = 8.0 Hz, 1H, CH_2), 3.94 (d, *J* = 14.8 Hz, 1H, CH_2), 4.08 (d, *J* = 15.2 Hz, 1H, CH_2), 5.40 (s, 1H, =CH), 7.29 (d, *J* = 8.0 Hz, 2H, Ar), 7.36 (d, *J* = 8.4 Hz, 2H, Ar), 7.64 (d, *J* = 8.0 Hz, 2H, Ar), 7.73 (d, *J* = 8.0 Hz, 2H, Ar). ^{13}C NMR ($CDCl_3$, 100 MHz, TMS) δ 21.4, 21.5, 26.1, 29.2, 38.0, 44.9, 45.6, 48.9, 54.4, 119.8, 121.2, 127.3, 127.5, 127.7, 129.6, 129.7, 129.9, 130.8, 131.9, 132.4, 133.1, 143.7, 143.8. IR (CH_2Cl_2) ν 2925, 2842, 1597, 1494, 1457, 1343, 1160, 1094, 1038, 700 cm^{-1}. MS (ESI) m/e 502.2 (M^++NH4). HRMS (ESI) calcd. for $C_{25}H_{32}N_3O_4S_2$: 484.1490, Found: 484.1494.

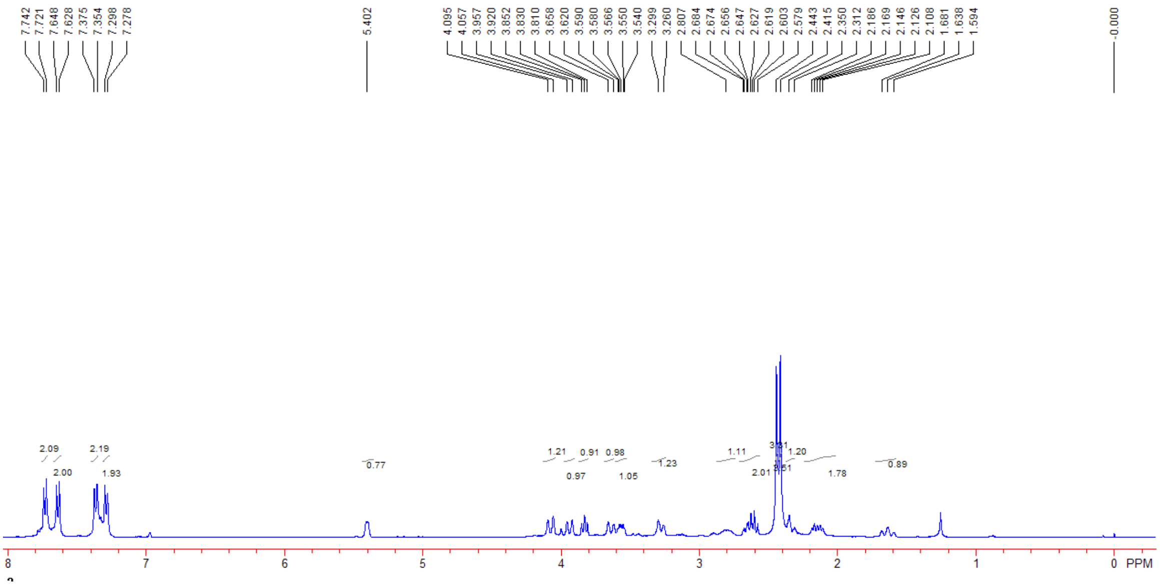

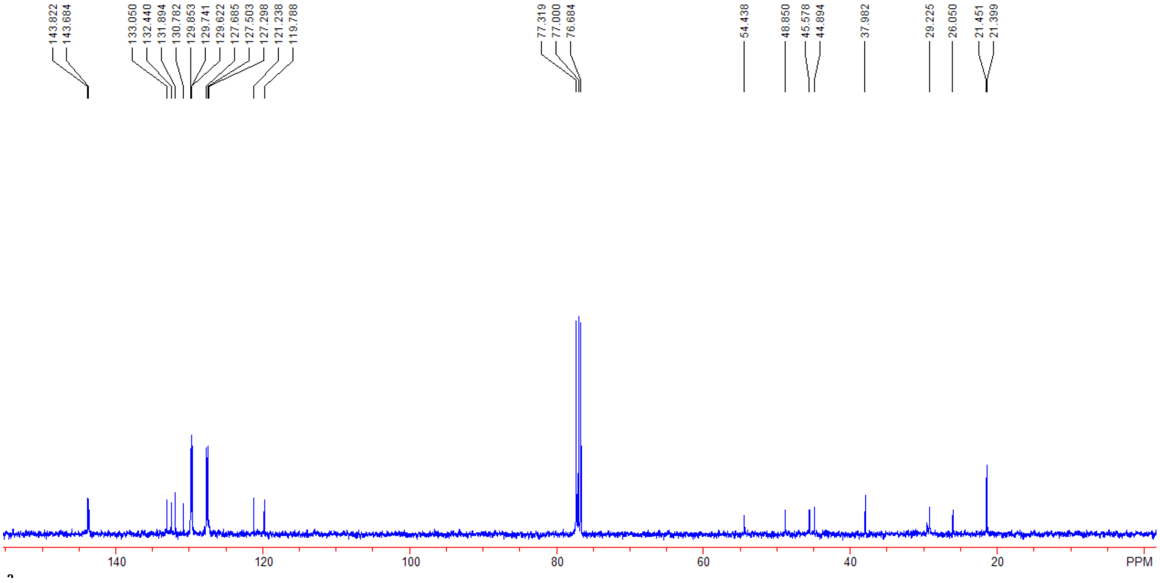

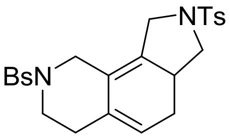

Compound 4l. 82 mg, yield: 75%, white solid, Mp: 220-222 °C. ^{1}H NMR ($CDCl_3$, 400 MHz, TMS) δ 1.63 (t, J = 17.6 Hz, 1H, CH_2), 2.12-2.20 (m, 1H, CH_2), 2.32-2.41 (m, 2H, CH and CH_2), 2.45 (s, 3H, CH_3), 2.60 (t, J = 9.6 Hz, 1H, CH_2), 2.72 (td, J_1 = 11.2 Hz, J_2 = 4.0 Hz, 1H, CH_2), 2.82 (brs, 1H, CH_2), 3.34 (d, J = 14.8 Hz, 1H, CH_2), 3.56-3.60 (m, 1H, CH_2), 3.64 (d, J = 16.0 Hz, 1H, CH_2), 3.84 (t, J = 8.8 Hz, 1H, CH_2), 3.96 (d, J = 14.8 Hz, 1H, CH_2), 4.08 (d, J = 16.0 Hz, 1H, CH_2), 5.41 (s, 1H, =CH), 7.37 (d, J = 8.0 Hz, 2H, Ar), 7.58-7.63 (m, 4H, Ar), 7.74 (d, J = 8.0 Hz, 2H, Ar). ^{13}C NMR ($CDCl_3$, 100 MHz, TMS) δ 21.5, 26.1, 29.1, 38.0, 44.9,

45.4, 48.9, 54.4, 120.0, 121.0, 127.8, 127.9, 128.9, 129.8, 130.6, 132.1, 132.3, 132.5, 135.5, 143.9. IR (CH_2Cl_2) ν 2925, 2856, 1727, 1597, 1455, 1343, 1231, 1161, 1102, 815, 667 cm^{-1}. MS (ESI) m/e 566.1 (M^{+}+NH_4). HRMS (ESI) calcd. for $C_{24}H_{25}BrN_2O_4S_2$: 548.0439, Found: 548.0445.

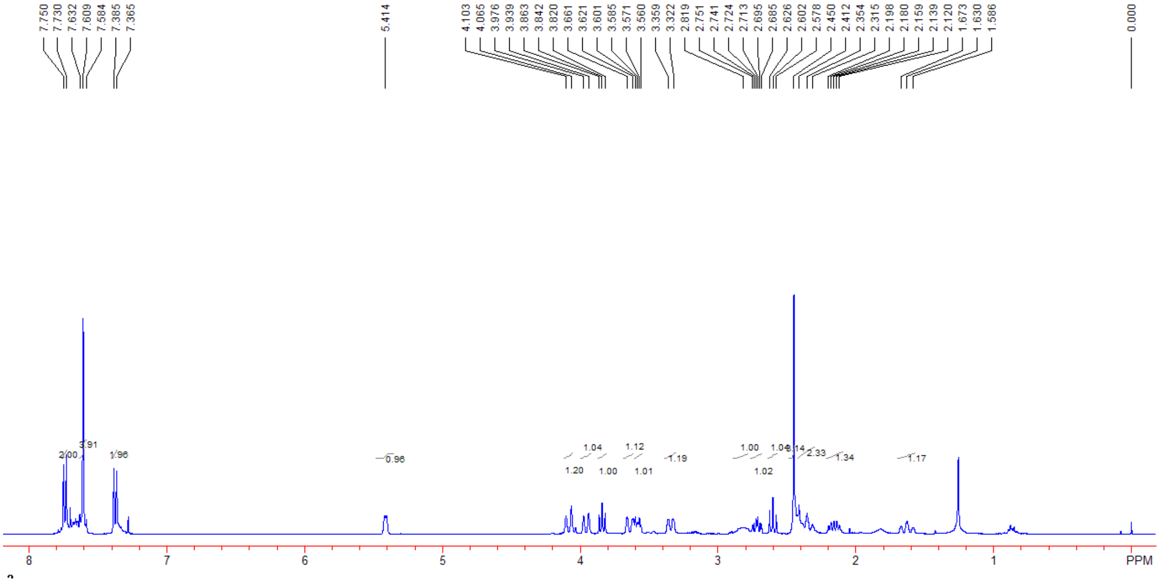

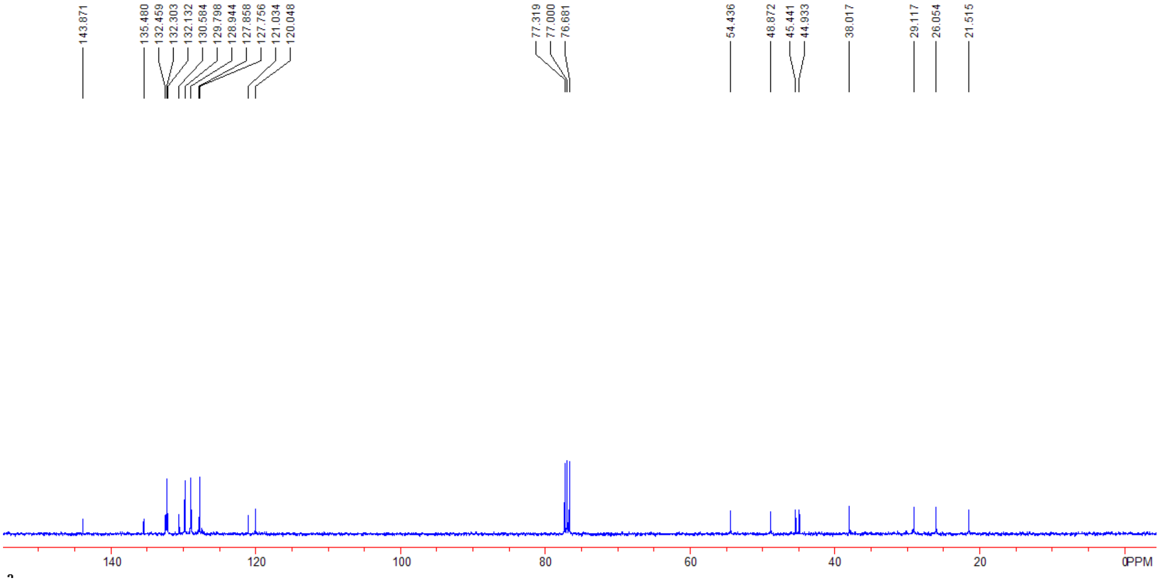

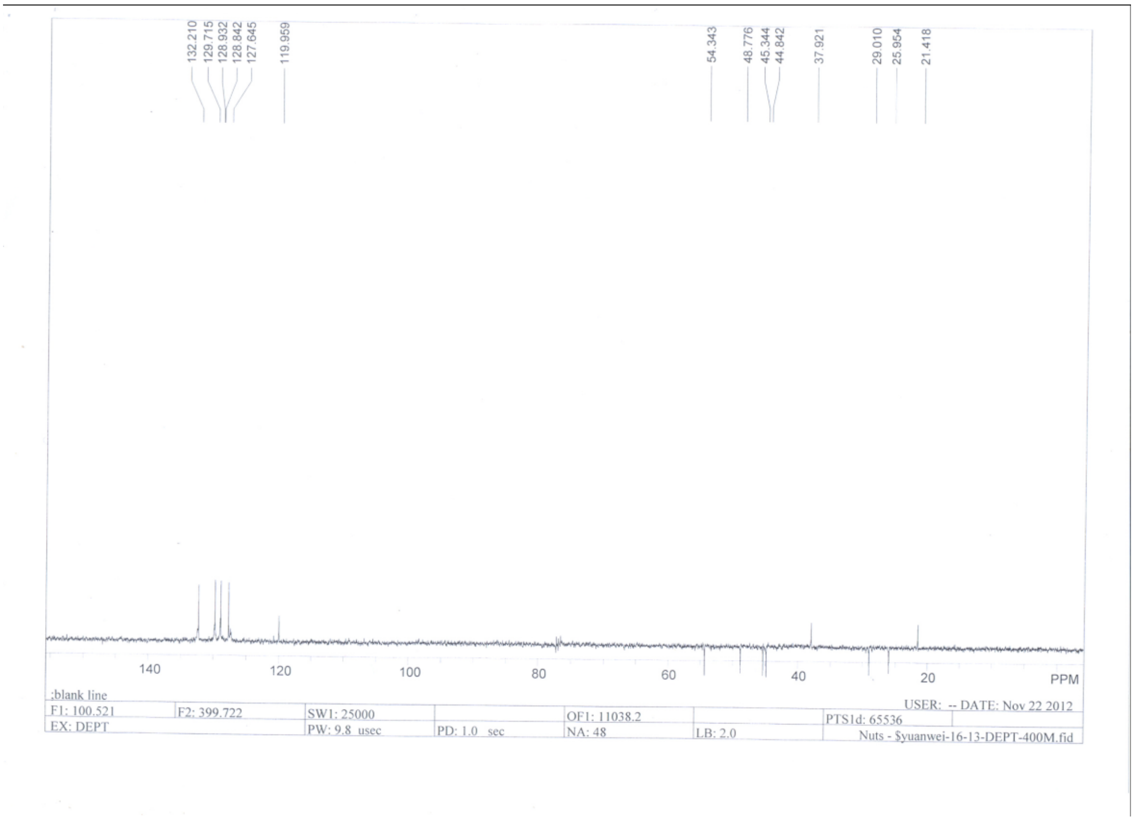
132.210
129.715
128.932
128.842
127.645
119.959
54.343
48.776
45.344
44.842
37.921
29.010
25.954
21.418
140
120
100
80
60
40
20
PPM
;blank line
USER: -- DATE: Nov 22 2012
F1: 100.521
F2: 399.722
SW1: 25000
OF1: 11038.2
PTS1d: 65536
EX: DEPT
PW: 9.8 usec
PD: 1.0 sec
NA: 48
LB: 2.0
Nuts - $yuanwei-16-13-DEPT-400M.fid

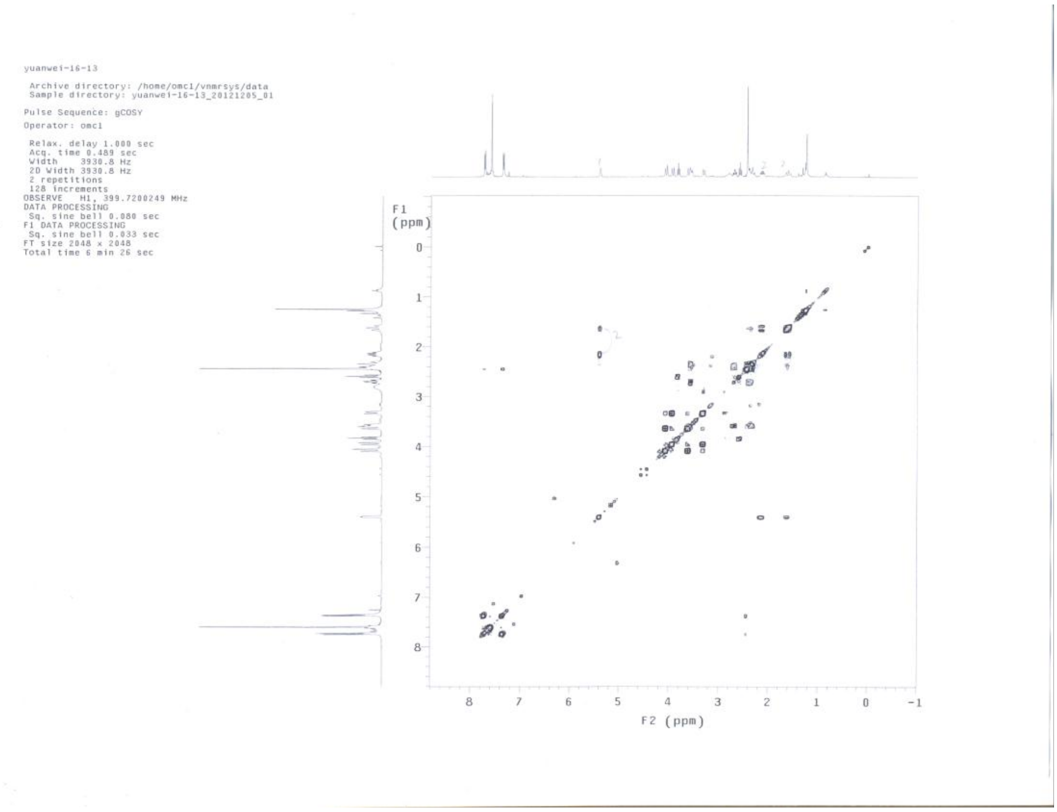
yuanwei-16-13
Archive directory: /home/omcl/vnmrsys/data
Sample directory: yuanwei-16-13_20121205_01
Pulse Sequence: gCOSY
Operator: omcl
Relax. delay 1.000 sec
Acq. time 0.489 sec
Width 3930.8 Hz
2D Width 3930.8 Hz
2 repetitions
128 increments
OBSERVE H1, 399.7200249 MHz
DATA PROCESSING
Sq. sine bell 0.080 sec
F1 DATA PROCESSING
Sq. sine bell 0.033 sec
FT size 2048 x 2048
Total time 6 min 26 sec
F1 (ppm)
F2 (ppm)

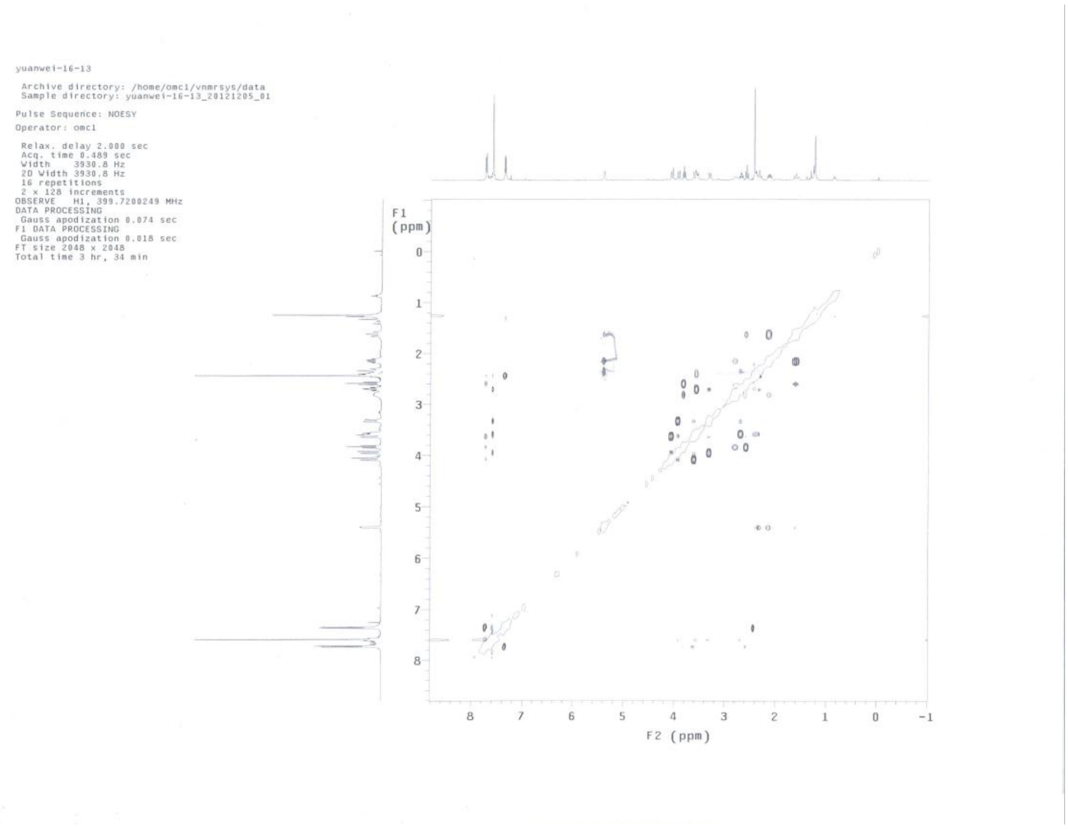
yuanwei-16-13
Archive directory: /home/omc1/vnmrsys/data
Sample directory: yuanwei-16-13_20121205_01
Pulse Sequence: NOESY
Operator: omc1
Relax. delay 2.000 sec
Acq. time 0.489 sec
Width 3930.8 Hz
2D Width 3930.8 Hz
16 repetitions
2 x 128 increments
OBSERVE H1, 399.7280249 MHz
DATA PROCESSING
Gauss apodization 0.074 sec
F1 DATA PROCESSING
Gauss apodization 0.018 sec
FT size 2048 x 2048
Total time 3 hr, 34 min
F1 (ppm)
F2 (ppm)

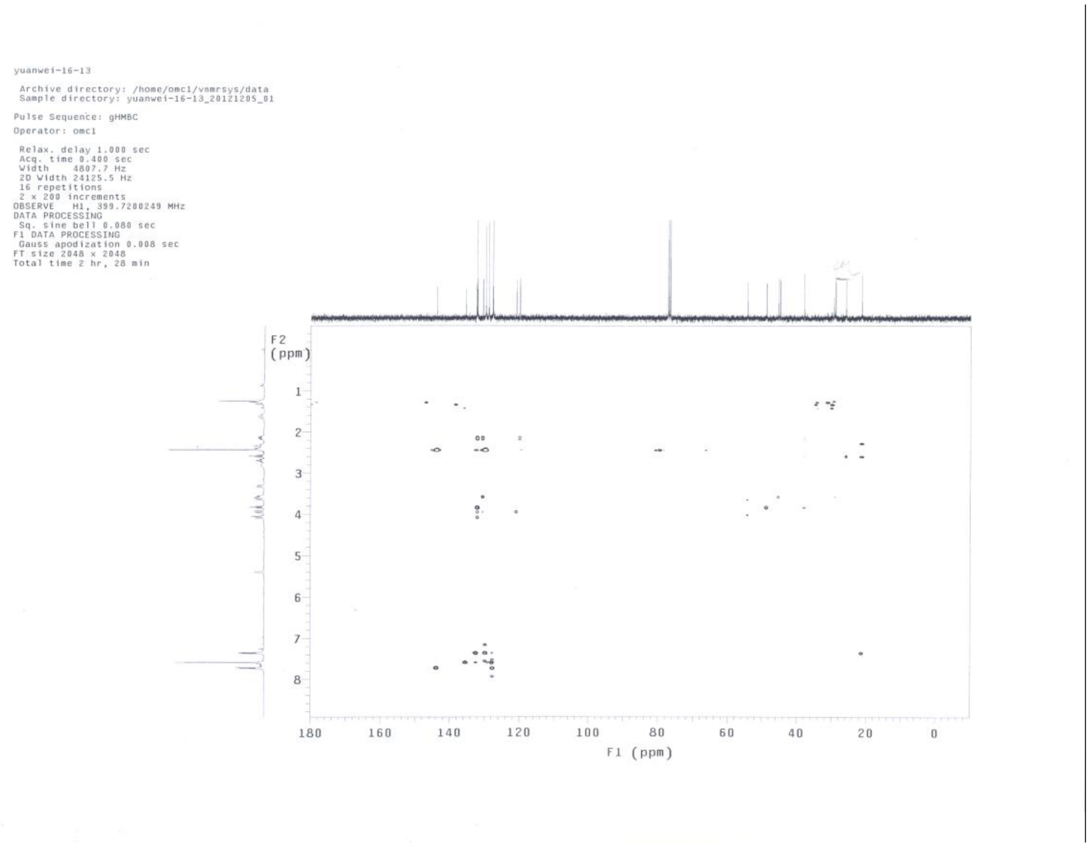
yuanwei-16-13
Archive directory: /home/omc1/vnmrsys/data
Sample directory: yuanwei-16-13_20121205_01
Pulse Sequence: gHMBC
Operator: omc1
Relax. delay 1.000 sec
Acq. time 0.400 sec
Width 4807.7 Hz
2D Width 24125.5 Hz
16 repetitions
2 x 200 increments
OBSERVE H1, 399.7280249 MHz
DATA PROCESSING
Sq. sine bell 0.080 sec
F1 DATA PROCESSING
Gauss apodization 0.008 sec
FT size 2048 x 2048
Total time 2 hr, 28 min
F2 (ppm)
F1 (ppm)

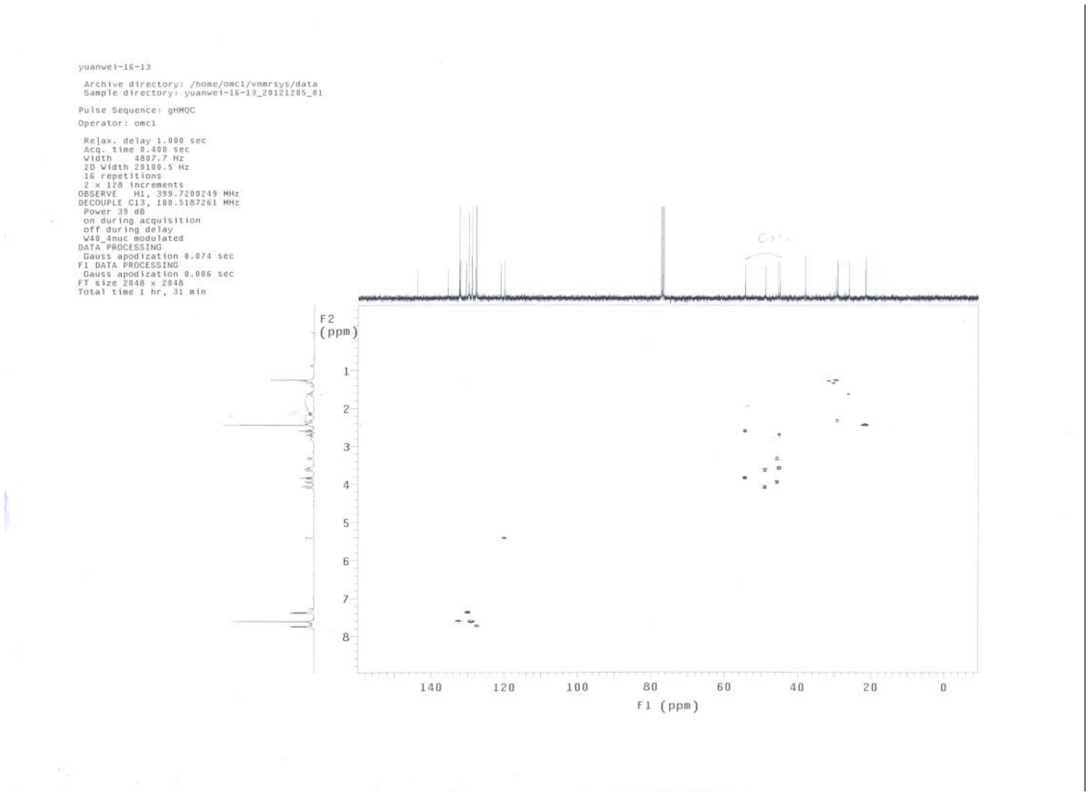
yuanwei-16-13
Archive directory: /home/omcl/vnmrsys/data
Sample directory: yuanwei-16-13_20121205_01
Pulse Sequence: gHMQC
Operator: omcl
Relax. delay 1.000 sec
Acq. time 0.400 sec
Width 4807.7 Hz
2D Width 20100.5 Hz
16 repetitions
2 x 128 increments
OBSERVE H1, 399.7200249 MHz
DECOUPLE C13, 100.5187261 MHz
Power 39 dB
on during acquisition
off during delay
W40_4nuc modulated
DATA PROCESSING
Gauss apodization 0.074 sec
F1 DATA PROCESSING
Gauss apodization 0.006 sec
FT size 2048 x 2048
Total time 1 hr, 31 min
F2 (ppm)
1
2
3
4
5
6
7
8
140
120
100
80
60
40
20
0
F1 (ppm)

Scheme SI-1. Another plausible reaction mechanism for the formation of [D]-**4h**.

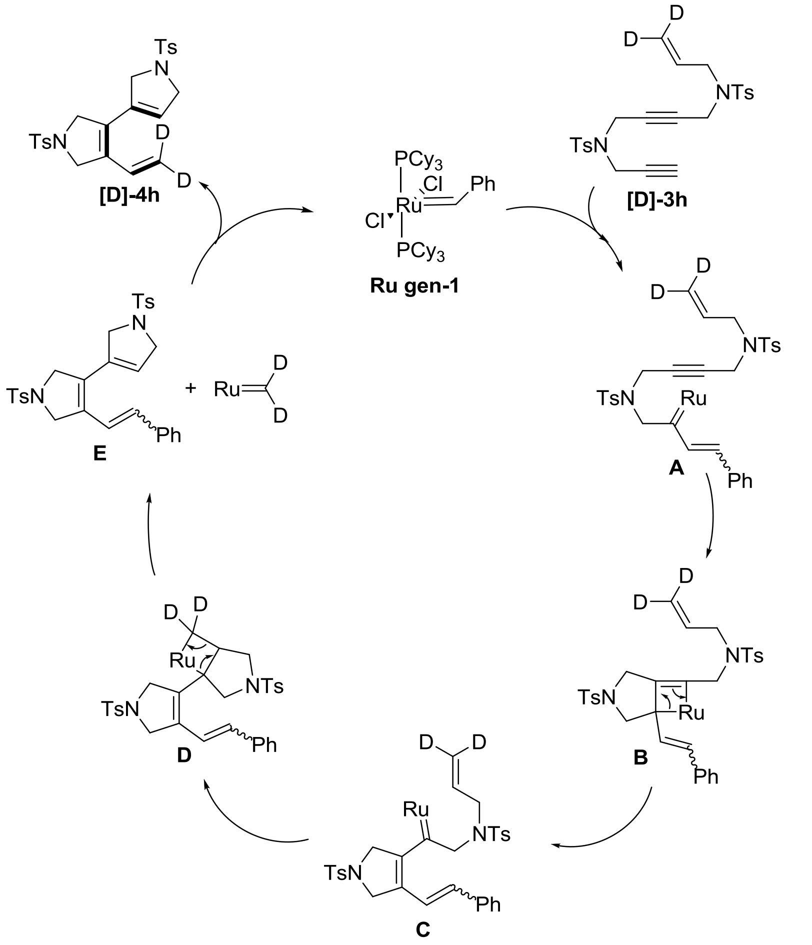

References

1. N. Nicolaus, S. Strauss, J. M. Neudörfl, A. Prokop, H. G. Schmalz, *Org. Lett.* **2009**, *11*, 341-344.

2. a) Y. Yamamoto, T. Arakawa, R. Ogawa, K. Itoh, *J. Am. Chem. Soc*. **2003**, *125*, 12143-12160; b) A. Geny, N. Agenet, L. Iannazzo, M. Malacria, C. Aubert, V. Gandon, *Angew. Chem*. **2009**, *121*, 1842-1845; *Angew. Chem. Int. Ed*. **2009**, *48*, 1810-1813.

3. a) Y.-X. Zhang, L. Guo, Y.-H. Wang, L.-L. Zhu, Z.-L. Chen, *Synthesis and Reactivity in Inorganic, Metal-Organic, and Nano-Metal Chemistry*, **2010**, *40*, 241-245; b) J. Liu, Y. An, H.-Y. Jiang, Z.-L. Chen, *Tetrahedron Lett.* **2008**, *49*, 490-494.
